# Signature of resistance gene evolution and pyrethroid resistance escalation in the major malaria vector *Anopheles funestus* across Kenyan malaria-endemic regions separated by the Rift Valley

**DOI:** 10.1186/s40249-026-01458-1

**Published:** 2026-05-15

**Authors:** David P. Tchouassi, Amine M. Mustapha, Gilbert Rotich, Trizah K. Milugo, Brenda Musimbi, Ambrose Oruni, Carlos S. D. Tagne, Luna Kamau, Mahamat Gadji, Magellan Tchouakui, Baldwyn Torto, Charles S. Wondji

**Affiliations:** 1 International Centre of Insect Physiology and Ecology, PO Box 30772-00100, Nairobi, Kenya; 2 Department of Zoology and Entomology, University of Pretoria, Private Bag X323, Pretoria, 0001 South Africa; 3 Centre for Research in Infectious Diseases, PO Box 13591, Yaoundé, Cameroon; 4 Centre for Biotechnology Research and Development, Kenya Medical Research Institute, PO Box 54840-00200, Nairobi, Kenya; 5 Vector Biology Department, Liverpool School of Tropical Medicine, Liverpool, L3 5QA UK

**Keywords:** Malaria, Transmission, Pyrethroid resistance escalation, *Anopheles funestus*, Rift Valley, Metabolic resistance, Resistance marker, Geographic barrier

## Abstract

**Background:**

Landscape features such as the Rift Valley (RV) can restrict gene flow in malaria vectors and influence resistance patterns. Here, we assessed *Plasmodium* infection rates, resistance alleles and profiles in *Anopheles funestus* s.s. populations across Kenyan malaria-endemic regions separated by the RV.

**Methods:**

*Anopheles funestus* s.s. populations in western, coastal and Kerio Valley (KV, within the RV) were genotyped for key resistance markers using polymerase chain reaction (PCR). *Taq*Man assay combined with nested PCR were used to screen the samples for *Plasmodium* sporozoite infection and association with resistance alleles and genotype frequencies assessed using Fisher’s exact test or Pearson’s chi-square test. Following established WHO guidelines, phenotypic resistance using F1 progeny was also assessed using diagnostic, intensity and piperonyl butoxide (PBO)-synergistic assays.

**Results:**

The 4.3kb-SV (*n* = 336) and *G454A-Cyp9k1* (*n* = 445) alleles were nearly fixed in western Kenya but declined towards the RV and coast, whereas L119F-GSTe2 (*n* = 392) increased across a west-KV-coast gradient with a novel haplotype distinct from known African variants detected at the coast. There were lower odds of *Plasmodium* infection in mosquitoes with L119F-GSTe2-RR than RS genotype (*OR* 0.2, *P* = 0.046). Likewise, mosquitoes harboring the R allele of the 4.3 kb marker had higher *Plasmodium* infection rates than the S allele (*OR* 5.7, *P* = 0.049). *An. funestus* populations exhibited a high degree of pyrethroid resistance with intensity higher in KV compared to western Kenya, a traditional malaria hotspot. Pre-exposure to PBO increased mortality for type II (deltamethrin, alpha-cypermethrin), than I (permethrin) pyrethroids, yet mortality remained lower in KV, suggesting non-P450-mediated resistance. *An. funestus* mosquitoes from the coast showed extreme permethrin resistance (< 10% mortality at 10 × dose). Resistance to dichlorodiphenyltrichloroethane was widespread, while all populations remained fully susceptible to bendiocarb, pirimiphos-methyl, clothianidin, and chlorfenapyr.

**Conclusions:**

Region-specific selection may drive varying resistance profiles in *An. funestus* across Kenyan malaria-endemic regions separated by the Rift Valley, with implications for malaria transmission and insecticide resistance management.

**Supplementary Information:**

The online version contains supplementary material available at 10.1186/s40249-026-01458-1.

## Background

Malaria remains a major vector-borne disease of significant medical and public health importance across much of sub-Saharan Africa (SSA). In 2024, the region accounted for 88% of the 282 million malaria cases reported [1]. Despite the widespread implementation of integrated control measures, including the use of insecticide-treated nets (ITNs), clinical case management such as proper diagnosis and treatment using artemisinin-based combination therapies (ACTs) [2], malaria incidence remains high in most African countries [1]. In Kenya alone, over four million cases were reported in 2024, representing a increase of 27% from the 3.29 million cases recorded in 2023 [1]. This persistently high burden highlights gaps in the understanding of the drivers of sustained transmission. Changes in vector behavior and ecological heterogeneity may be contributing to ongoing transmission dynamics and undermining the effectiveness of current control measures.

Humans become infected with malaria parasites through the bites of parasite-infected female *Anopheles* mosquitoes, which vary in their vectorial capacity—potential to transmit pathogens. Among the major malaria vectors in SSA, is *Anopheles funestus* s.s. (referred herein as *An. funestus*), a species characterised by high susceptibility to *Plasmodium* parasites, a strong anthropophilic (human-biting) preference and prolonged adult longevity [3, 4]. In the wake of declining *An. gambiae* populations likely due to up-scale of ITNs, *An. funestus* has emerged as a dominant malaria vector across much of East Africa, including several regions in Kenya [3–5]. Furthermore, *An. funestus* has the tendency to alter behaviour, highly adaptive—breeding throughout the year and can rapidly develop resistance to insecticides [6–8].

Insecticide resistance poses a major challenge to the long-term effectiveness of current vector control tools and malaria control strategies [9]. Across much of Africa, *An. funestus* populations have developed resistance to pyrethroids, the primary class of insecticides used in public health [10–12]. Moreover, there is growing evidence of resistance extending to other insecticide classes recommended by the World Health Organization (WHO) [13]. The worsening situation of insecticide resistance is currently further exacerbated by the growing threat of resistance escalation—characterized by the ability of mosquito populations to survive very high doses of insecticides, thereby reducing the efficacy of control interventions [10–12]. In *An. funestus,* resistance is predominantly mediated by overexpression of key metabolic genes, particularly those encoding cytochrome P450s enzymes (CYPs), including *CYP6P9a/b*, C*YP6P4a/b*, and *CYP9K*1, as well as glutathione-transferase epsilon 2 (*GSTe*2) [14–16]. Moreover, genetic variants within key metabolic genes and structural variants (SVs) such as the 6.5kb-SV and 4.3kb-SV have also been implicated [17, 18]. Additionally, recent studies have identified target site mutations, conferring localized knockdown resistance (*kdr)* [19] and non-coding RNAs [20] in resistance mechanisms.

In Kenya, reduced susceptibility of *An. funestus* to pyrethroids have been documented in malaria endemic regions, such as coastal and western regions [5, 21, 22]. However, the underlying molecular mechanisms remain poorly understood, underscoring the need for comprehensive investigations into the evolution and spread of insecticide resistance *An. funestus* populations across diverse ecological settings.

In Africa, *Anopheles funestus* populations are widely distributed and exhibit significant local genetic variability, which may underlie their high adaptive traits [23]. Environmental pressures—both climatic and anthropogenic, including the widespread use of insecticides, are likely contributors to this adaptability, as has been documented in *An. gambiae* s.l. [24]. Moreover, geographical and ecological factors that can affect gene flow, such as physical barriers and spatial distance may limit mosquito dispersal, leading to distinct population structure and hence, response to vector control interventions.

Previous studies have proposed that the East African Rift Valley may function as a geographic barrier influencing gene flow and genetic differentiation in malaria vectors such as *An. gambiae* [25] and *An. funestus* [26, 27]. This landscape feature may similarly affect the spatial dynamics of resistance alleles, potentially restricting their spread across regions [28]. Consequently, such barriers could influence the efficacy of insecticide-based interventions, local malaria transmission patterns, and overall control outcomes.

This study investigated whether the Rift Valley—as a known geographic barrier—impacts gene flow and contributes to variation in resistance genotypes and phenotypes among *An. funestus* populations in Kenya. Specifically, we characterised the bionomics and genetic variants associated with known metabolic resistance genes in *An. funestus* populations spanning major malaria endemic areas across the Rift Valley.

## Methods

### Study sites

**Fig. 1:**
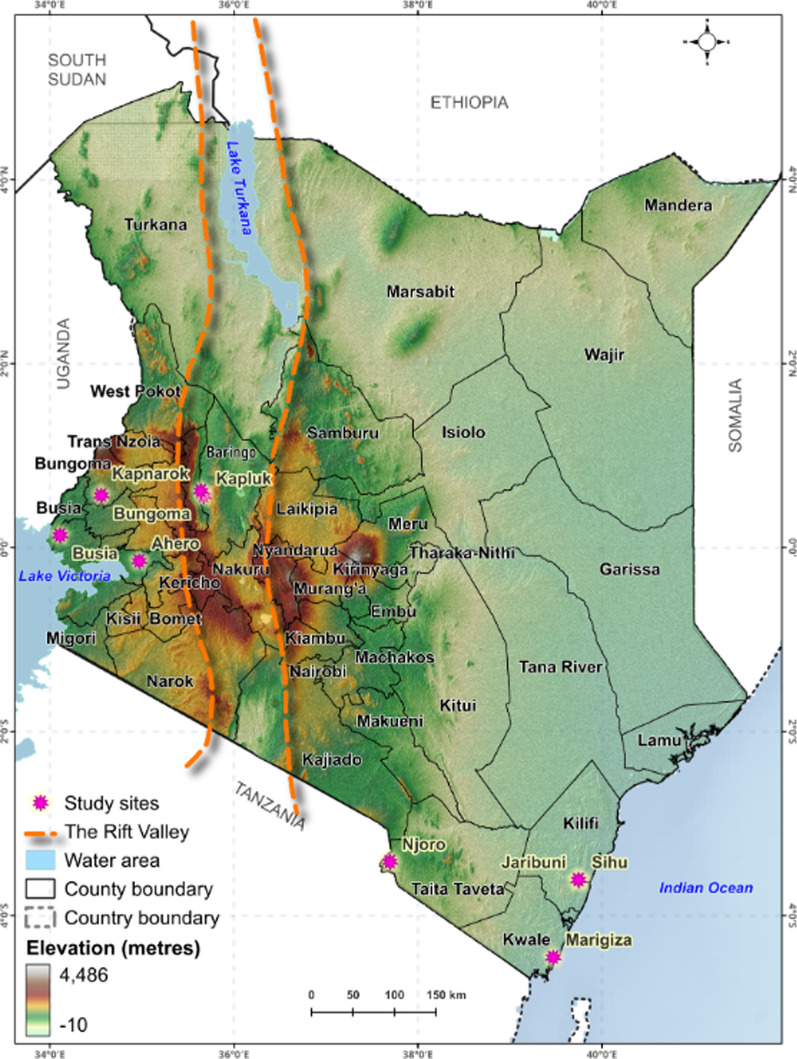
Kenyan map indicating the study sites along the Rift Valley divide. Map designed by Juliet Onditi@*icipe*, Nairobi

Adult anophelines were collected in selected sites within the Rift Valley (RV): Kerio Valley (KV) comprising Kapluk and Kapnarok in Baringo County; west of the RV: Ahero (Kisumu County), Busia (Busia County), Bungoma (Bungoma County) in western Kenya; and east of the RV in the coastal sites of Taveta, Marigiza (Kwale County) and Sihu/Jaribuni (Kilifi County). These sites encompass the major malaria risk zones in Kenya (Fig. 1). Western Kenya in the Lake malaria-endemic zone has some of the highest prevalence of malaria followed by the coastal sites in the Coast malaria-endemic zone [2]. For instance, Busia, Bungoma, and Kisumu in western Kenya are among the highest malaria burden counties in terms of malaria endemicity (10–30% prevalence) and only moderately in Kwale county (< 5% prevalence). Both western and coastal Kenya experience year-round malaria transmission with peaks linked to the short (October–December) and long (March–May) rainy seasons. In contrast, Baringo County categorized as seasonal malaria-epidemic zones are generally considered low risk with intense transmission in the rainy season [29]. Up to 16% malaria prevalence has been reported in parts of the county as in the KV largely attributed to *Plasmodium falciparum* [30].

The main malaria vectors in Kenya include members in the *Anopheles gambiae* s.l. and *An. funestus* species complexes although the relative importance of the species in malaria transmission varies by epidemiologic risk zones, sites and seasons [31]. *An. funestus* are among primary malaria vectors in the study sites encompassing the major malaria risk zones in Kenya [3, 29].

### Mosquito collections

In each of the sites, surveillance of night-active host-seeking adult anophelines included simultaneous trapping inside and outside of randomly consenting households using CDC light traps (model 512, John W. Hock Co, Gainesville, Florida, USA). Important household features considered were house design, roof type (grass thatched or corrugated iron sheets) and presence of eaves. In each household, one was placed indoors and another outdoors between February 2021 and September 2022. The outdoor traps were additionally baited with dry ice. In each site, ten light traps were set daily between 18:00–06:00 during each session for 35 consecutive nights, targeting different households. Inside each household, the trap was set near a bed (foot side of an occupant). The collected mosquitoes were transported in liquid N_2_ to the laboratory at the *icipe* Duduville Campus, Nairobi, and later − 80 °C freezer until processing.

Subsequently, indoor resting, blood-fed female *An*. *funestus* were collected on walls and roofs of selected houses using battery-powered prokopack aspirators in sites representative of the broad ecological areas: western (Busia), Coast (Kilifi and Kwale), and KV (Kapnarok/Kapluk). The collection was carried out between 3:00 and 6:00, for 3 consecutive days, following verbal consent from the chief of the district and the household owners. The mosquito collection was conducted between June and October 2024. Aspirated mosquitoes were kept in cages and then morphologically identified using keys by [32] and separated into *An. funestus s.l*, from other anophelines or culicines with provision of 10% sucrose solution and kept for 3–5 days until gravid.

### Mosquito rearing

Eggs obtained using the forced-egg laying method [i.e., placed individually in 1.5 ml Microcentrifuge tubes (Biologix, Shandong, China)] were pooled into rearing trays (per site). The hatched larvae fed on Tetramin (Tetramin1, Melle, Germany) ad libitum were reared until F_1_ adult generation at *icipe* Duduville Campus in Nairobi and used for insecticide exposure assays. All mosquitoes were reared under standard insectary conditions at a temperature of between 26 ± 2 °C with 65–85% relative humidity and under a 12:12 photoperiod of natural light. Larval water (mineral water, Mount Kenya Ltd) was changed every three days until pupation. Emerged adults were kept in Bugdorm cages (MegaView Science Co., Ltd, Taichung, Taiwan, China) while being given 10% sugar solution before bioassays.

### Insecticide susceptibility tests

To fully characterise the resistance of *An. funestus* populations, insecticide susceptibility assays were carried out using 2–5-day-old non-blood-fed *An. funestus* s.s. F_1_ adults (WHO tube or bottle assays protocol) [32] to a range of insecticides (Additional file 1: Table S1). This included type I (permethrin) and II pyrethroids (deltamethrin and alpha-cypermethrin), the carbamate bendiocarb, the organochlorine dichlorodiphenyltrichloroethane (DDT), the organophosphates fenitrothion and pirimiphos-methyl, and the newly approved WHO neonicotinoid clothianidin and pyrrole chlorfenapyr. Insecticide resistance was examined not only against standard diagnostic concentrations, but also intensity assays for pyrethroid insecticides only. Two to five replicates of around 15–25 mosquitoes per tube were exposed to insecticide impregnated filter papers for 1 h and then transferred to a clean holding tube supplied with 10% sugar. Mortalities were determined 24 h after exposure.

Additionally, cytochrome P450 genes involvement in metabolic resistance was assessed using PBO (piperonyl butoxide), an inhibitor of P450 activity. Mosquitoes exposed to non-impregnated papers were included as controls. These bioassays were conducted at 26 ± 2 °C and 70 ± 10% relative humidity.

### Mosquito identification

A sample of the field-derived host-seeking mosquitoes were sorted and *Anopheles funestus* s.l. morphologically identified using keys [33]. Genomic DNA (gDNA) was extracted from head/thorax and abdomen separately in each individually processed *An. funestus* s.l. mosquitoes using the Livak protocol [34]. Extracted gDNA from the abdomen was used to identify the sibling species of *An. funestus* group via species-specific polymerase chain reaction (PCR) [35]. Individual oviposited *An. funestus* s.l. females from resting collections were similarly identified.

### *Plasmodium* infection

gDNA from the head/thorax (F_0_ *An. funestus* only) was processed to detect the presence of *Plasmodium* infections via real-time *Taq*Man PCR assay [36], with further confirmation of positive specimens using the nested PCR based approach by Snounou et al. [37], and protocol described in Fonkou et al. [38]. Positive mosquitoes were assumed to be positive for sporozoites.

### Genotyping of resistance markers and sequencing the* GSTe2* gene in *An. funestus* s.s.

Individual first generation (F_0_) *An. funestus* s.s. specimens (*n* = 139–445) identified from trap collections were genotyped for four selected validated markers cytochrome P450 *CYP6P9a* gene (*Cyp6P9a*), mutation L119F in the glutathione S-transferase epsilon 2 (*GSTe*2) gene (*L119F-GSTe*2)*,* 4.3kb transposon-containing structural variant (4.3kb-SV), and the mutation G454A in the cytochrome P450 *CYP9K1* gene (*G454A-Cyp9K1*). This was achieved by allele-specific PCR (AS-PCR) method and/or restriction fragment length polymorphism as described [14, 16, 18, 35]. The PCR products of L119F-*GSTe*2 only, corresponding to different genotypes, were purified using ExoSAP-IT (Thermo Fisher Scientific Inc, Carlsbad, USA) and Sanger sequenced using the forward primer only. The obtained sequences (*GSTe-*2) were cleaned and aligned using MEGA v7 [39]. The sequences were compared with reference *GSTe*2 sequences [14] deposited in GenBank. Maximum likelihood trees were inferred using the best-fit model of sequence evolution, with nodal support for different groupings evaluated through 1000 bootstrap replications.

### Polymorphism analysis of *GSTe*2 gene in *An. funestus* across Kenya

We compared the *GSTe*2 alleles obtained in Kenya with those from other African regions, incorporating published sequences of the *GSTe*2 gene [14] in the GenBank (accession numbers KC800340–KC800421). The sequences are included in Additional file 2: Table S2. These sequences were used for Africa-wide comparative genetic analysis. Genetic polymorphisms were determined through manual examination of *GSTe2* coding sequences using BioEdit version 7.2.3.0 [40] and sequence differences in multiple alignments using ClustalW sequence analyser in the BioEdit software with default parameters. Construction of a phylogenetic maximum likelihood tree was done using MEGA v7 [39]. A best-fit substitution model was tested based on Bayesian information criteria using Tamura-2 parameter which best described the sequence dataset. The model was then used with 1000 bootstrap replicates and a maximum likelihood tree generated. Haplotype network analysis was plotted using the Templeton Crandall Singleton (TCS) and TCS beautifier to beautify the generated haplotype network [41].

### Data analysis

Data was entered into an excel sheet to plot counts, proportions and frequencies. The distribution of mutations for each marker was assessed by determining allelic and genotype frequencies. *Plasmodium* infection rates among the genotypes/alleles for each marker were compared using the Fisher’s Exact Test/Pearson’s Chi-square tests. GraphPad Prism (version 10.6.1; GraphPad Inc., La Jolla, CA, USA) and/or R v 4.5.1 software (The R Foundation for Statistical Computing) was used for data analysis at 95% confidence limit.

## Results

### Species distribution

**Fig. 2:**
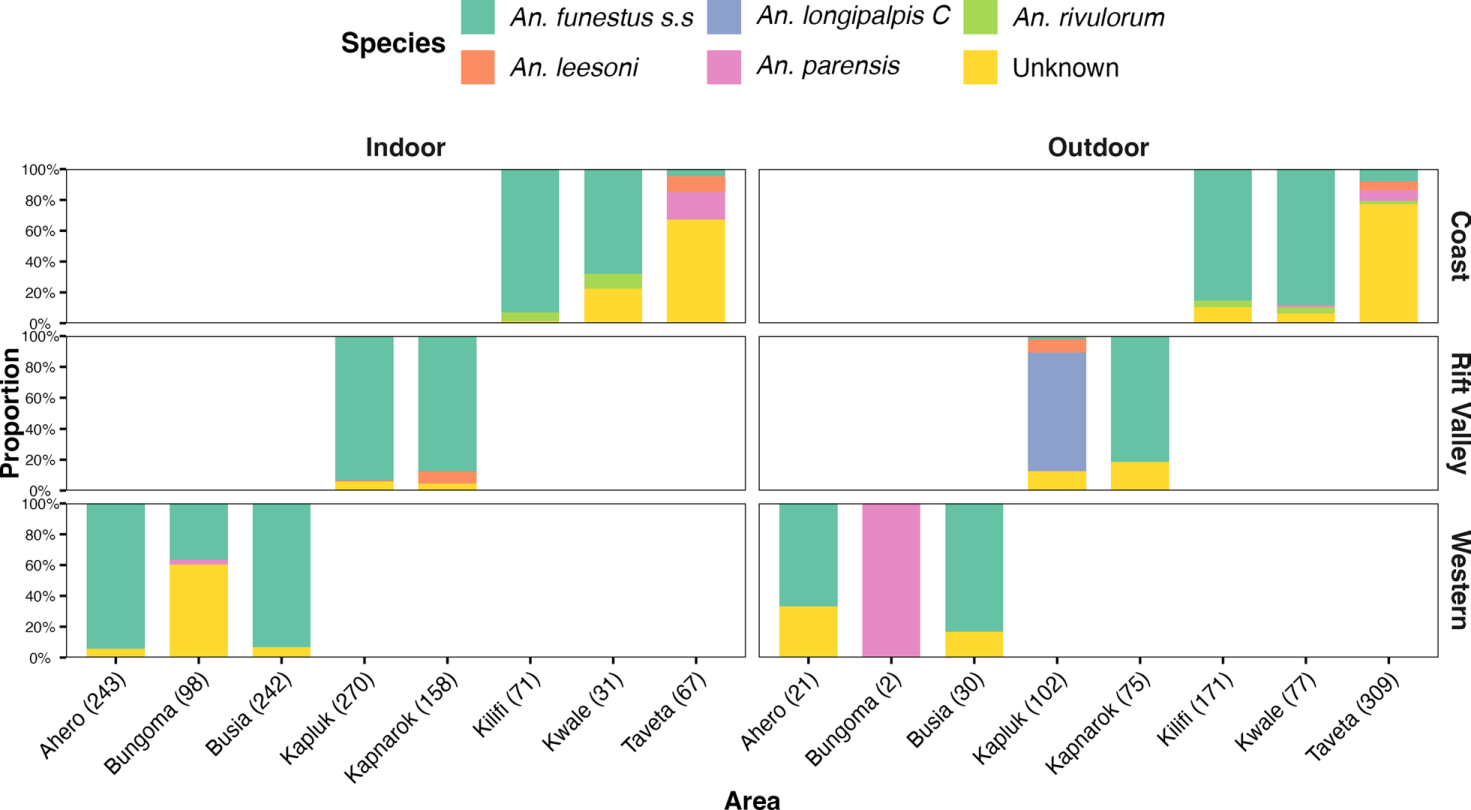
Relative abundance of *Anopheles funestus* to other sibling species and distribution indoors and outdoors

A total of 1967 *An. funestus* s.l. specimens were processed by PCR, with *An. funestus* emerging as the predominant sibling species, accounting for 66.6% (1310/1967) of the total. In contrast, captures of *An. rivulorum*, *An. leesoni*, *An. parensis*, and *An. longipalpis* C were comparatively low. Notably, *An. longipalpis C* was detected exclusively at Kapluk. A considerable proportion (23.7%) of mosquitoes captured in Taveta (coastal site) failed to amplify during molecular analysis. Interestingly, *An. funestus* was the dominant species captured indoors across western sites (Ahero, Busia, and Bungoma) and Kerio Valley locations (Kapluk and Kapnarok) (Fig. 2). The species was predominant outdoors in Kwale, Kilifi and Taveta at the coast (Fig. 2).

### *Plasmodium* parasite infection rates and association with key resistance markers

A subset of *An. funestus* (*n* = 463) was analysed for infection with *Plasmodium* sporozoite and genotyped for metabolic resistance gene markers; *L119F-GSTe*2 and *G454A-Cyp9K1* and the structural variant 4.3kb-SV. Of these, 8.2% (range 3.3–44.4%) tested positive for *Plasmodium* sporozoite infection (95% *P. falciparum;* 5% *P. ovale*) with variation among sites (Table 1). Cumulative *Plasmodium* infection rates were highest among locations in western (12.7%; 21/165; range 5.7–44.4%), followed by KV (7.1%; 7/99; range 5.5–9.1) and then coast (5.1%; 10/196; range 3.3–9.5%).

**Table 1:** *Plasmodium* sporozoite infection rates in *Anopheles funestus* collected in selected sites spanning major malaria epidemiological risk zones of western, coast and Rift Valley regions of Kenya

Region	Site	*Plasmodium* infection rates (proportion)
Western	Busia	10.6 (10/94)
	Bungoma	44.4 (8/18)
	Ahero	5.7 (3/53)
Rift Valley (Kerio Valley)	Kapnarok	5.5 (3/55)
	Kapluk	9.1 (4/44)
Coast	Marigiza-Kwale	7.1 (4/56)
	Taveta	9.5 (2/21)
	Jaribuni-Kilifi	3.3 (4/122)
	Total	**8.2 (38/463)**

**Fig. 3:**
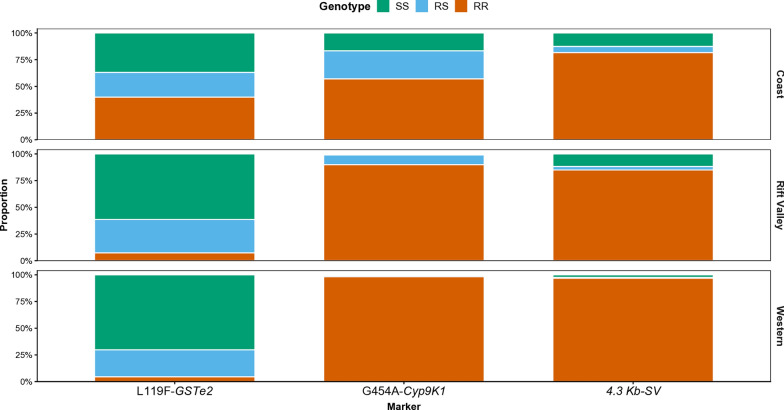
Frequency of genotypes of key resistance markers detected in *Anopheles funestus* collected from different regions spanning major malaria epidemiological risk zones in Kenya. The genotypes homozygous susceptible, SS; heterozygous resistant, RS and homozygous resistant, RR

Genotype frequencies for three key metabolic resistance markers; *L119F-GSTe*2, *G454A-Cyp9K1*, and 4.3kb-SV transposon insertion, were assessed in *An. funestus* populations from western, Rift Valley and coastal regions (Fig. 3) and the results revealed striking regional contrasts. The *L119F-GSTe*2, successfully assessed in 392 specimens, yielded an overall allele frequency of 0.33 (range 0.16–0.74). The resistant *119F-GSTe*2 allele was most prevalent at the coast (55/138; 39.9%), at lower frequency in KV (7/95; 7.4%), and least frequent in western Kenya (7/159; 4.4%) (Fig. 3). By contrast, genotyping of the *G454A-Cyp9K1 (n* = 445) and 4.3kb-SV (*n* = 336) revealed much higher frequencies of the RR genotype, approaching fixation in western Kenya (~ 98.8%), followed by the Rift Valley (~ 91%), and the coastal sites of Kwale and Kilifi (~ 82%) (Fig. 3). For the *CYP6P9a* marker (*n* = 139), the SS genotype was detected in varying proportions in KV (24/139), western region (85/129) and the coast (30/139).

**Fig. 4:**
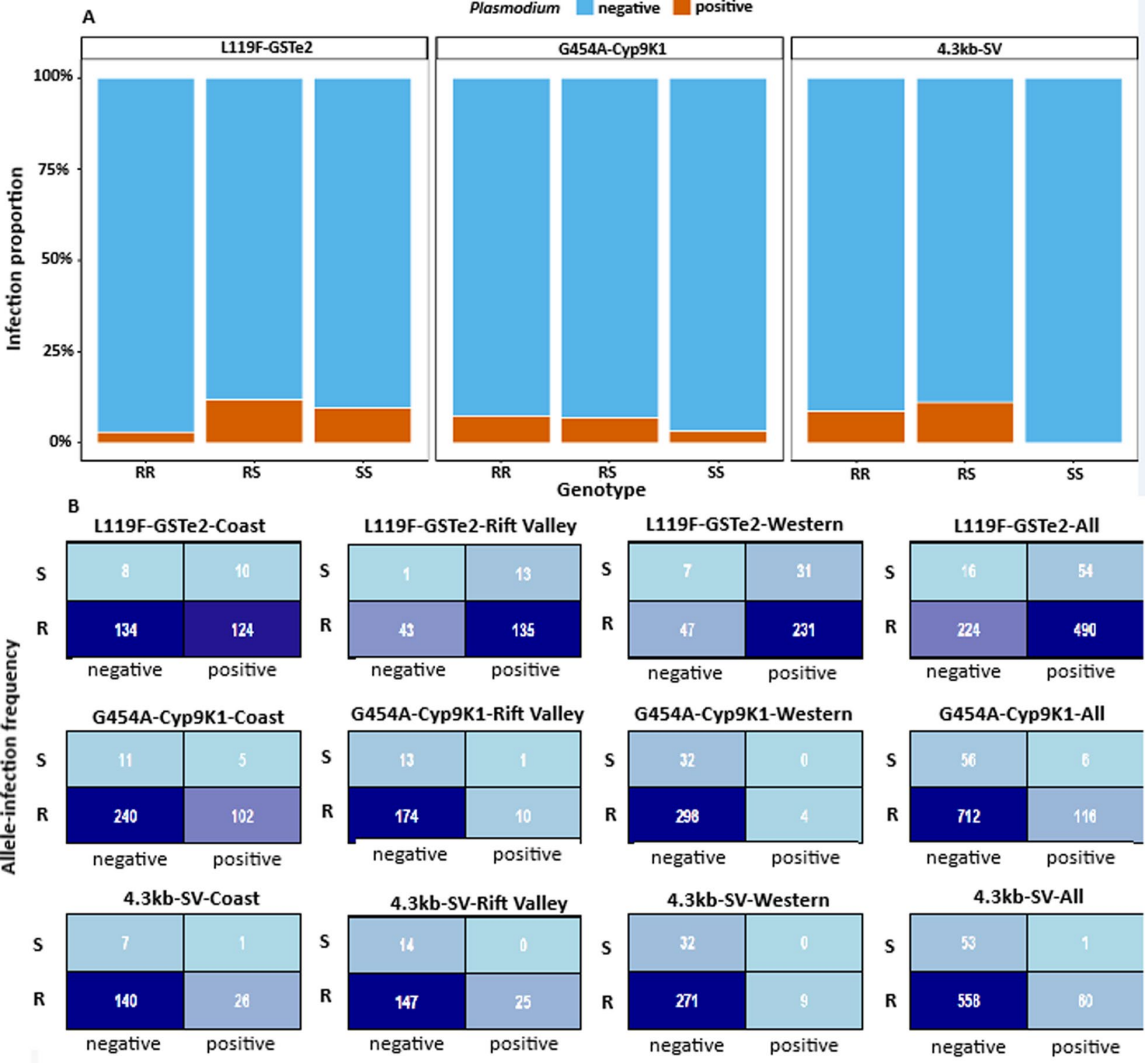
Association between resistant markers and *Plasmodium* sporozoite infection. **A** shows the distribution of negative and positive *Plasmodium* sporozoite results among the genotypes for each marker; RR, homozygous resistant; RS, heterozygote; SS, homozygous susceptible. **B** shows the 2 × 2 contingency table to evaluate the association between the resistant alleles R (resistant) and S (susceptible) with counted positive and negative *Plasmodium* sporozoite samples with the lower table showing calculated p-values for each the association between each marker resistant allele and *Plasmodium* infection by region and then combined across all samples

We next compared the distribution of genotypes between *Plasmodium*-infected and non-infected specimens across the detected resistance markers. For both *G454A-Cyp9K1* and 4.3kb-SV, infection was more frequently observed among individuals carrying the RR genotype (Fig. 4A; Additional file 3: Fig. S1). The breakdown by marker was as follows: *G454A-Cyp9K1* (RR = 26/355; RS = 4/58; SS = 1/32) and 4.3kb-SV (RR = 26/301; RS = 1/9; SS = 0/26). A test of association found no significant difference in infection rates between the genotypes for 4.3kb-SV (*χ*^*2*^ = 2.53, *df* = 2, *P* = 0.28) and *G454A-Cyp9K1* (χ^2^ = 0.80*, df* = *2, P* = 0.67) (Fig. 4B). Further analysis at the allelic level revealed that by combining all samples from different regions hence increasing sample size for phenotype-genotype association, only the R individuals with the 4.3kb-SV significantly carried higher infection than S (OR 5.7, *P* = 0.049) (Additional file 4: Fig. S2, Additional file 5: Fig. S3). In contrast, for the *L119F-GSTe*2 marker, revealed an opposite trend, with a higher proportion of infections occurring in individuals carrying the SS (21/221) or RS (12/102) than RR (2/69) genotype (Fig. 4A). There was a fivefold significant likelihood of parasite infection in mosquitoes carrying the RS than RR genotype for *L119F-GSTe*2 (Additional file 3: Fig. S1). Nonetheless, there was no significant difference in infection prevalence between the genotypes (*χ*^*2*^ = 4.18, *df* = 2, *P* = 0.12) and alleles (*P* = 0.18), for this marker (Fig. 4B, Additional file 3: Fig. S1, Additional file 4: Fig. S2, Additional file 5: Fig. S3). Although not statistically significant (*P* = 0.18), the observed trend suggests a potential negative association between the resistant *119F-GSte2* allele and parasite infection.

### Sequence analysis of the *GSTe2* gene

**Fig. 5:**
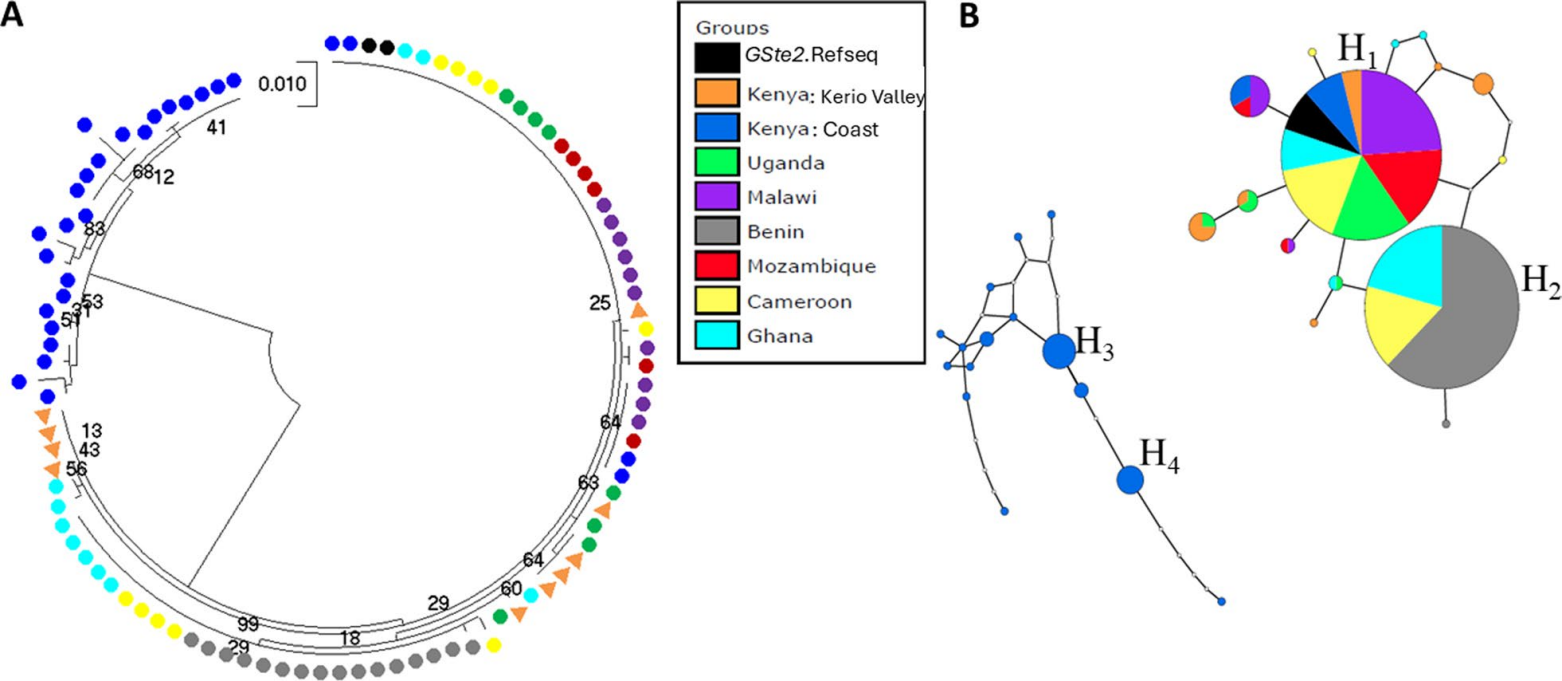
Africa-wide genetic analysis of the coding region of glutathione S-transferase epsilon 2 (*GSTe*2) gene. **A** Maximum likelihood phylogenetic tree. **B** Haplotype network. The Kenyan haplotypes generated in this study have been deposited in NCBI GenBank (accession numbers: PX686378-PX686415)

A segment of the *GSTe2* gene (666 bp) was sequenced among selected Kenyan genotypes to infer the evolutionary relationship with reference sequences generated across Africa. A pronounced genetic differentiation was evident between the Coastal Kenyan populations and rest of other African regions (Fig. 5). The analysis identified two major clades, a primary clade that included sequences from several African countries, including Benin, Uganda, Malawi, Mozambique, Ghana, Cameroon, and sequences from Rift Valley of Kenya (Fig. 5A). A second clade contained sequences exclusively from Coastal site of Kenya. Further, genetic differentiation was demonstrated by the haplotype network analysis which revealed two distinct networks, one containing two dominant haplotypes (H_1_ and H_2_) comprising sequences from various geographical regions in Africa, including West Africa (Ghana and Benin), southern Africa (Malawi and Mozambique), Central Africa (Cameroon), and eastern Africa (Uganda and Rift Valley Kenya) (Fig. 5B). Conversely, the second haplotype network included sequences exclusively from Coastal Kenya, featuring two major haplotypes (H_3_ and H_4_) specific to this Kenyan region. Overall, the analyses revealed high genetic divergence in *GSTe2* sequences between most *An. funestus* populations from Coastal Kenya and those from the rest of Kenya.

### Nucleotide polymorphism analysis within the *GSTe*2 gene *in An. funestus* across Kenya

**Fig. 6:**
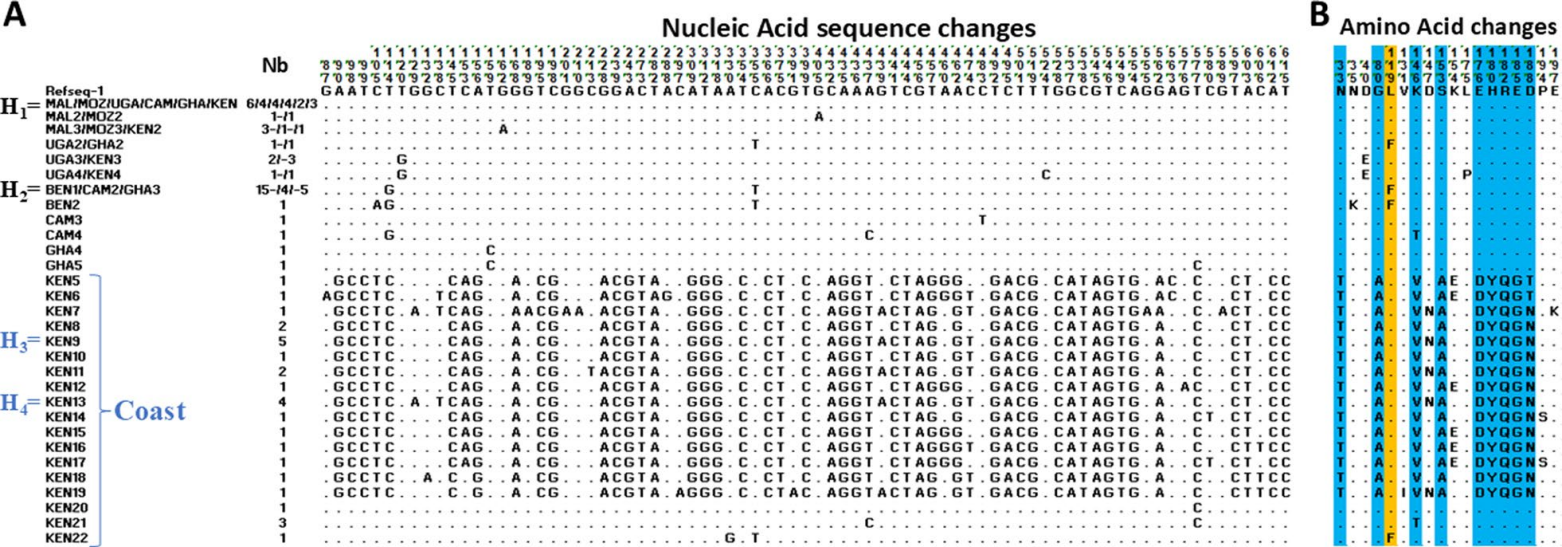
Nucleotide diversity analysis representation of Africa-wide *GSTe*2 coding sequence. **A** Nucleic acid sequences. **B** Amino acid sequences, with the *L119F-GSTe*2 marker highlighted in yellow and changes in Kenyan Coastal samples highlighted in blue. Kenya (KEN) haplotypes with numbers are indicated similar for Benin (BEN), Uganda (UGA), Cameroon (CAM), Malawi (MAL) and Mozambique (MOZ)

Nucleotide polymorphism analysis was performed to investigate the nucleotide changes encompassing the different haplotypes of the *GSTe*2 gene across Africa. The analysis shows that primary dominant haplotype (H_1_) common to all African samples (Fig. 6A, B) is characterized by the absence of single nucleotide polymorphism (SNP) across the *GSTe*2 open reading frame (ORF). Twenty-three (23) out of 98 sequences examined harboured this haplotype. The secondary dominant haplotype (H_2_) found at high frequency in Benin (15 sequences), moderate frequencies in Cameroon (4 sequences) and Ghana (5 sequences) is characterized by a single nucleotide change: a cytosine (C) to thymine nucleotide transition at position 355, resulting in an amino acid change from leucine (L) to phenylalanine (F) on codon 119. The third and fourth haplotype (H_3_ and H_4_), which are exclusively found in Coastal Kenya, have multiple SNPs that produce a unique protein sequence. This protein sequence is marked by 10 amino acid changes in linkage disequilibrium: asparagine (N) to thymine (T) at codon 33, a glycine (G) to alanine (A) at codon 80, lysine (K) changed to valine (V) at codon 146, aspartate (D) to asparagine (N) at codon 147, serine (S) to alanine (A) at codon 153, glutamate (E) to aspartate (D) at codon 176, histidine (H) to tyrosine (Y) at codon 180, arginine (R) to glutamine (Q) at codon 182, glutamate (E) to glycine (G) at codon 185 and aspartate (D) to asparagine (N) at codon 188 (T^33^ A^80^ V^146^ N^147^ A^153^ D^176^ Y^180^ Q^182^ G^185^ N^188^). This Coastal Kenyan haplotype is present at a moderate frequency, with 10 out of 30 sequences harbouring this specific haplotype.

### Insecticide resistance profile

**Fig. 7:**
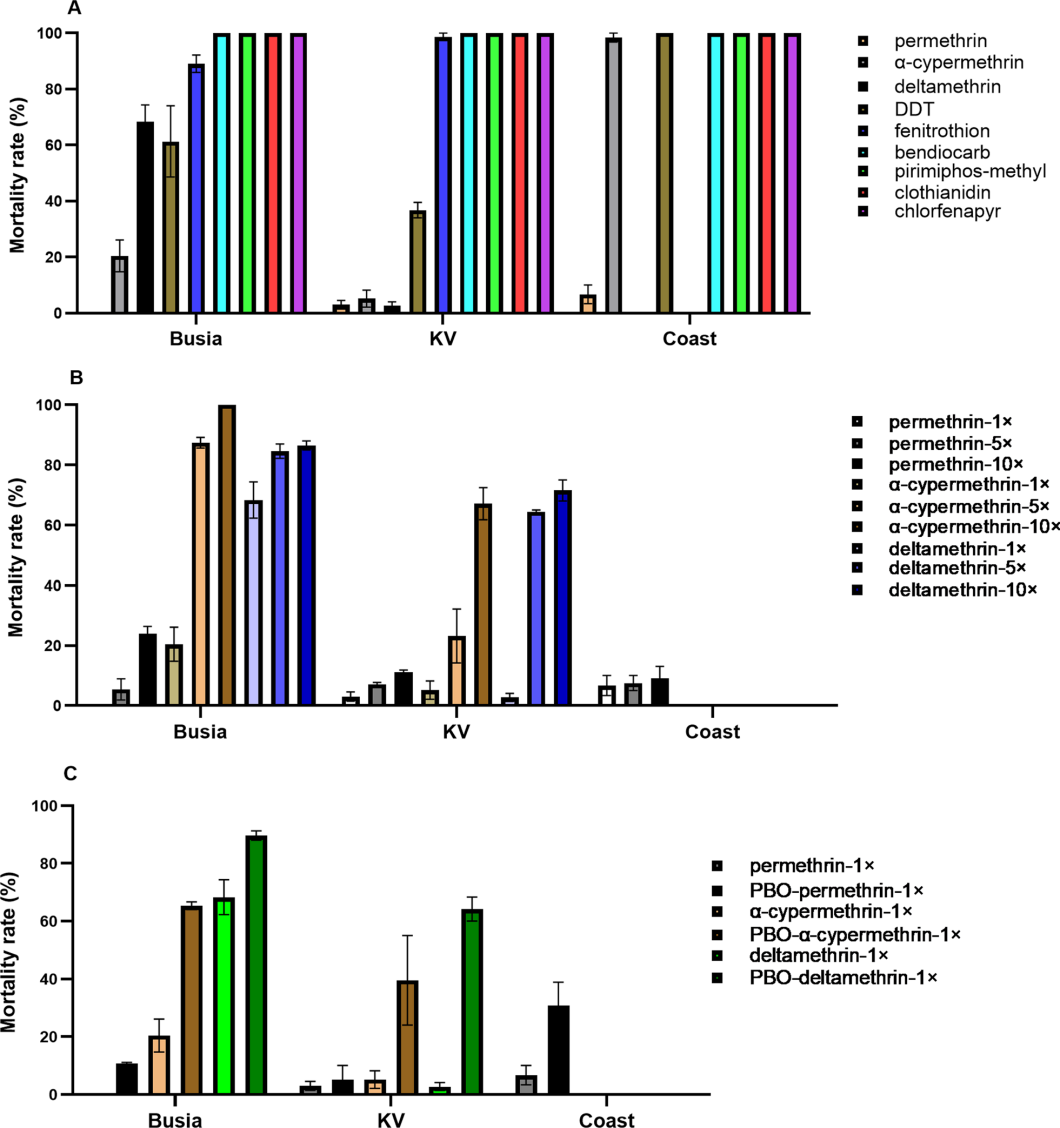
Insecticide resistance patterns of *Anopheles funestus* populations from Busia, Kerio Valley, and Coast to discriminatory insecticide doses (**a**), intensity assays (**b**) and synergist assays with PBO to pyrethroids (**c**), respectively. Per, permethrin; α-cyp, alpha-cypermethrin; delta, deltamethrin; PBO, piperonyl butoxide; *An. funestus* mosquitoes tested in Busia and KV comprised *An. funestus* s.s. and those from Coast may not be exclusively *An. funestus* s.s

Insecticide resistance and intensity were higher for type 1 (permethrin; overall mean mortality = 3.2% across sites) than type II (deltamethrin, alpha-cypermethrin, with respective mean mortalities across sites being 35.5% and 41.3%) pyrethroids and was more pronounced for *An. funestus* in RV than western Kenya, a traditional malaria hotspot (Fig. 7A, B). Increasing dosage only had a modest effect on mortality with permethrin in KV (average mortality: 1 × = 3%, 5 × = 7.1% and 10 × = 11.2%) and Busia (average mortality: 1 × = 0%, 5 × = 5.4% and 10 × = 24%). Mortality increased with an increasing dosage of α-cypermethrin in Busia: (average mortality; 1 × = 20.4%, 5 × = 87.4.1% and 10 × = 100%) but not so much in KV (average mortality; KV: 1 × = 3.2%, 5 × = 5.2% and 10 × = 23.2%). With increasing concentration, the mortality greatly increased with deltamethrin in Busia (average mortality: 1 × = 68.3%, 5 × = 84.6% and 10 × = 86.5%) and KV: average mortality; 1 × = 2.7%, 5 × = 64.5% and 10 × = 71.5%) (Fig. 7a–c).

Pre-exposure to PBO notably increased the mortality with alpha-cypermethrin (mean mortality = 39.5% and 65.3% in KV and Busia, respectively) and deltamethrin (mean mortality = 64.2% and 89.6% in KV and Busia, respectively). However, only a modest increase in mortality with PBO was observed with permethrin in KV (mean mortality = 5%), lower than in Busia (mean mortality = 10.8%). Coast population exhibited intense resistance to permethrin only (1 × = 6.7%, 5 × = 7.5% and 10 × = 9.1%) and similar low mortality in PBO synergist assay with this insecticide (mean mortality = 30.8%). Both Busia and KV populations were resistant to DDT (mean mortality; KV: 36.8%; Busia = 68.3%) except coast that was susceptible (mean mortality = 98.4%). By contrast, all three populations were fully susceptible (mortality = 100%) to bendiocarb, pirimiphos-methyl, clothianidin and chlorfenapyr.

Oviposited *F*_*0*_* An. funestus* s.l. mosquitoes (from which F1 progeny used for resistance exposure was derived) identified by cocktail PCR revealed 99% (129/130) and 99% (125/126) were *An. funestus* from KV and Busia, respectively. In contrast, only a minor proportion of *An. funestus* s.l. from the coast was *An. funestus* (5%), with the majority being *An. rivulorum* (80%, 72/90).

## Discussion

Investigating the spread of resistance markers is critical for insecticide resistance monitoring in malaria vectors, gauging insight into associated mechanisms and informing control strategies. In this study, vector bionomics, distribution of key metabolic resistance markers and resistance profiles to several insecticides were investigated among *An. funestus* populations along the Rift Valley divide spanning major malaria risk zones of Kenya. Spatial distribution analysis revealed two distinct patterns; that in both KV and western sites, *An. funestus* is the predominant sibling species in the Funestus group and mostly encountered indoors. The coast, was also dominated by *An. funestus* in some sites which were mostly encountered outdoors, consistent with previous results [3, 43, 44]. The result might indicate divergence in the biting and resting behavior of this mosquito at the coast from western/KV areas. High outdoor biting behavior of malaria vectors could be associated with low exposure to ITNs consequently affecting expression of insecticide resistance differently among the vector populations. The divergence could be ascribed to a combination of factors, including vector adaptation to human behavior, climate, and even genetics [43]. Previous studies found genetic divergence of coastal *An. funestus* populations from other areas in Kenya [27, 45] but genetic heterogeneities that underpin indoor versus outdoor habits and perhaps biting times should be investigated further.

The study also revealed a high *Plasmodium* sporozoite infection rate in *An. funestus*, comparable or even higher than values observed for this species in Central [46] and East Africa [3, 10, 12, 47, 48]. Notably, study sites in western Kenya displayed the overall highest *Plasmodium* infection rates, followed by KV and then coast. The results suggest underlying difference in competence of vector populations or levels of usage or protection provided by ITNs and/or indoor residual spraying (IRS). Evidence of sustained high Plasmodial transmission and infection resurgence in western Kenya despite intensified malaria control interventions since 2006 [49], indicate the need to investigate this trend further. The vector infection results confirm the active role this mosquito species plays in contemporary and persistent malaria transmission [3, 4]. The other sibling species or unknown species were most prevalent at the coast. Because these other sibling species, including cryptic species [3, 29, 44, 50, 51] are equally important in transmission, characterization of their resistance profiles remains primordial. Further longitudinal surveys will address seasonal dynamics of these species and role in malaria transmission.

Our study reveals distinct spatial gradients in metabolic resistance marker allele frequencies among Kenyan *An. funestus* populations, with *G454A-Cyp9K1* and the 4.3kb-SV increasing from the western regions through the Rift Valley to the coast, while *L199F-GSTe*2 declines, suggesting region-specific selection pressures. The *CYP6P9a* mutation was rare, restricted to homozygous susceptible genotypes in western and Rift Valley sites, consistent with the limited geographic spread of the mutant variants of the southern Africa *CYP6P9* markers into eastern Africa [15, 52] except central west and south to northeastern Tanzania [19]. Although the *GSTe*2 RR genotype was predominant at the coast, east of the Rift Valley, mosquitoes carrying the susceptible *GSTe*2 SS genotype were more frequently infected with *Plasmodium*, contrasting with Central African studies reporting higher parasite prevalence among resistant genotypes [38, 42]. The discovery of a novel *GSTe*2 haplotype unique to coastal populations perhaps further explains this contrast and further supports differential local selection pressures that could impact vector-parasite interaction. Interestingly, we observed a strong positive association between the 4.3kb-SV mutant alleles and parasite infection, opposite to prior reports where infection was lower in mosquitoes having this variant [18]. This discrepancy may reflect regional differences of the impact of this marker on gene expression, vector competence, environmental adaptation, or insecticide-driven selection that could modulate parasite susceptibility. The G454A mutation in *Cyp9K1*, a major driver of type II pyrethroid resistance in East and Central Africa [16], approached fixation alongside the 4.3kb-SV, consistent with observations in eastern Uganda [10, 12]. Presence of multi-locus combinations of *L119F-GSTe*2, *G454A-Cyp9K1*, and 4.3kb-SV alleles did not correlate with infection status. Incorporating additional parasite detection methods that can improve estimates of *Plasmodium* sporozoite infection or methodologies that correct for potential overestimation [53] may allow for better inferences on genotype-phenotype association. Known antagonistic interactions between *CYP6P9a/b* and the *4.3-kb SV* [18] and the cumulative impact of multiple resistance factors on extreme pyrethroid resistance (> 1000-fold) highlight the complex adaptive landscape. Collectively, these findings reveal heterogeneous selection acting on key resistance genes across Kenyan populations, shaped by ecological variation and local insecticide exposure, and underscore the need for genomic studies to clarify adaptive signatures, potential fitness costs, and their implications for malaria transmission.

This study provides the first evidence of pyrethroid resistance escalation in Kenyan *Anopheles funestus*, using 5 × and 10 × discriminating concentrations. Resistance intensity varied markedly across regions, with coastal populations exhibiting higher mortality than those from the Kerio Valley (Rift Valley) and Busia (western Kenya). Species composition likely contributes to this pattern: coastal samples were dominated by *An. rivulorum* (< 5% *An. funestus*), whereas KV and Busia populations were > 99% *An. funestus*. Whether these sibling species differ in resistance mechanisms remains unclear, but this may partly explain the lower resistance observed at the coast. The low mortality to permethrin at 10 × concentration is highly concerning and aligns with recent reports of pyrethroid resistance in coastal *An. funestus* [54]. Notably, resistance intensity in KV was higher than in Busia, possibly reflecting intensified insecticide selection from agricultural practices such as cotton cultivation, which relies heavily on pyrethroids, compared with public health interventions like ITNs and IRS that dominate the coast and western Kenya [55].

Synergist assays revealed low mortality following pre-exposure to PBO, particularly with permethrin in KV, suggesting that cytochrome P450-mediated detoxification is an important but not exclusive mechanism driving resistance. The persistence of high resistance despite PBO exposure indicates that other metabolic pathways, cuticular modifications, or additional genetic factors may also contribute. Moreover, the presence of multilocus resistance genotypes, including combinations of *Cyp9K1*, 4.3kb-SV, and *GSTe*2, suggests complex interactions that may influence both insecticide tolerance and vectorial capacity. These findings highlight the need for molecular and genomic studies to unravel the adaptive signatures and functional mechanisms underlying resistance escalation in these populations [56].

Encouragingly, full susceptibility was observed to bendiocarb in all populations, in contrast to previous and recent reports in Uganda [10, 57], indicating that this carbamate remains a viable alternative for IRS in these localities. Full susceptibility to organophosphates and newer chemistries, including pyrimiphos-methyl, clothianidin, and chlorfenapyr, further expands the portfolio of tools for pyrethroid resistance management [10, 58, 59]. The escalation of pyrethroid resistance documented here has clear implications for vector control efficacy, particularly for ITN and IRS strategies [11] and similar trends have been observed in the neighboring country, Uganda [10, 12]. The observed heterogeneity in resistance across species, regions, and selection pressures emphasizes the necessity for locally tailored interventions and sustained resistance monitoring to anticipate shifts in adaptive dynamics and inform effective malaria control programs.

One limitation of this study was the geographic scope of the sites used for phenotypic resistance which we assumed were representative of each malaria risk zone. Insecticide resistance can vary seasonally, geography and environmental conditions [60, 61]; our cross-sectional design may not provide a stable pattern of the resistance profile underscoring the need to examine temporal consistency through additional longitudinal assessments. Further, establishing how our resistance results relate to insecticide use in agriculture, households including bed net use could shed light on their relative contributions to resistance development and underlying drivers. Nonetheless, our study represents a significant advancement characterizing resistance profile of multiple populations of this species in Kenya, notoriously difficult to colonize. Establishing further associations between the genetic resistance markers and phenotypic resistance could benefit from additional assessment of their relative distribution among dead and alive specimens as well as gene transcripts among surviving cohorts [10, 11, 61]. Transcriptomic analysis of the samples is currently being pursued and should provide greater insights into underlying molecular mechanisms potentially driving resistance in the mosquito populations. Essential data through long term nationwide surveillance characterizing frequency of resistance markers combined with phenotypic resistance and malaria transmission indices should guide interventions by the national malaria control program in the context of evolving resistance.

## Conclusions

The study confirms high malaria parasite infection in *An. funestus* populations exhibiting pyrethroid resistance escalation and different evolutionary histories in resistance genes (at least in *GSTe*2) along a west-RV-coast gradient in Kenya. The role of the Rift Valley should be considered in insecticide resistance spread and effective resistance management in Kenya. Further work is needed to elucidate the underlying molecular basis of resistance escalation and the significance of the observed resistance mutations on malaria transmission.

## Supplementary Information

Additional file 1: Table S1. List of insecticides tested

Additional file 2: Table S2. Sequences of *GSTe*2 haplotypes analysed from *Anopheles funestus* in Kenya and other African countries.

Additional file 3: Fig. S1. Odds ratio versus genotype comparisons among *Anopheles funestus* mosquitoes infected with *Plasmodium* sporozoites.

Additional file 4: Fig. S2. Odds ratio versus allele comparisons among *Anopheles funestus* mosquitoes infected with *Plasmodium* sporozoites.

Additional file 5: Fig. S3. Summary statistics of allele-infection associations for the different markers in *Anopheles funestus* populations from Kenya.

## Data Availability

All data generated or analyzed during this study are included in this published article and its additional files.

## Abbreviations

ACTs: Artemisinin-based combination therapies
CYPs: Cytochrome P450s enzymes
CDC: Centers for Disease Control and Prevention
DNA: Deoxyribonucleic acid
DDT: Dichlorodiphenyltrichloroethane
GST: Glutathione S-Transferase
IRS: Indoor residual spraying
ITNs: Insecticide-treated nets
kdr: Knock-down resistance
KV: Kerio Valley
*OR*: Odds ratio
ORF: Open reading frame
PBO: Piperonyl butoxide
PCR: Polymerase chain reaction
RV: Rift Valley
s.l.: Sensu lato
s.s.: Sensu stricto
SV: Structural variant
WHO: World Health Organization
SSA: Sub-Saharan Africa
