# Supplementary figures and images for: Signature of resistance gene evolution and pyrethroid resistance escalation in the major malaria vector Anopheles funestus across Kenyan malaria-endemic regions separated by the Rift Valley
Source: Infect Dis Poverty. 2026 May 15;15:57. doi: 10.1186/s40249-026-01458-1 (PMC13179624; doi:10.1186/s40249-026-01458-1)

**L119F-GSTe2**

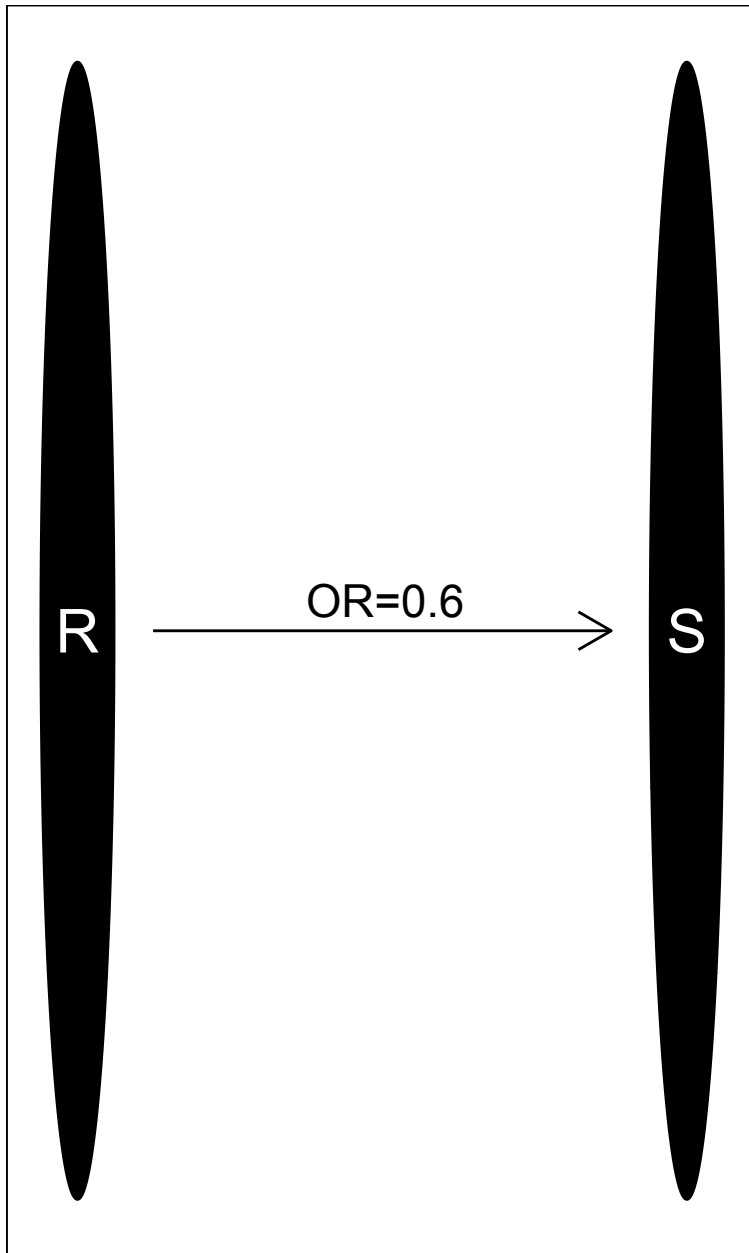

**G454A-Cyp9K1**

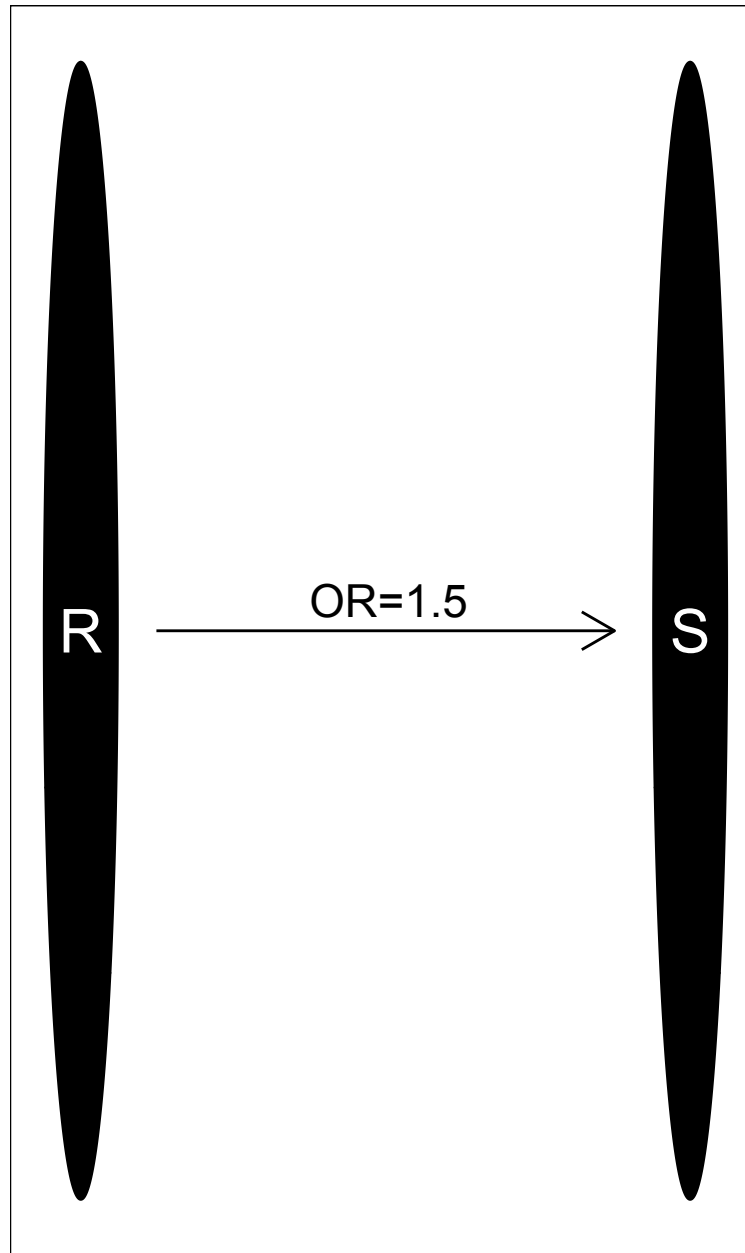

**4.3 Kb-SV**

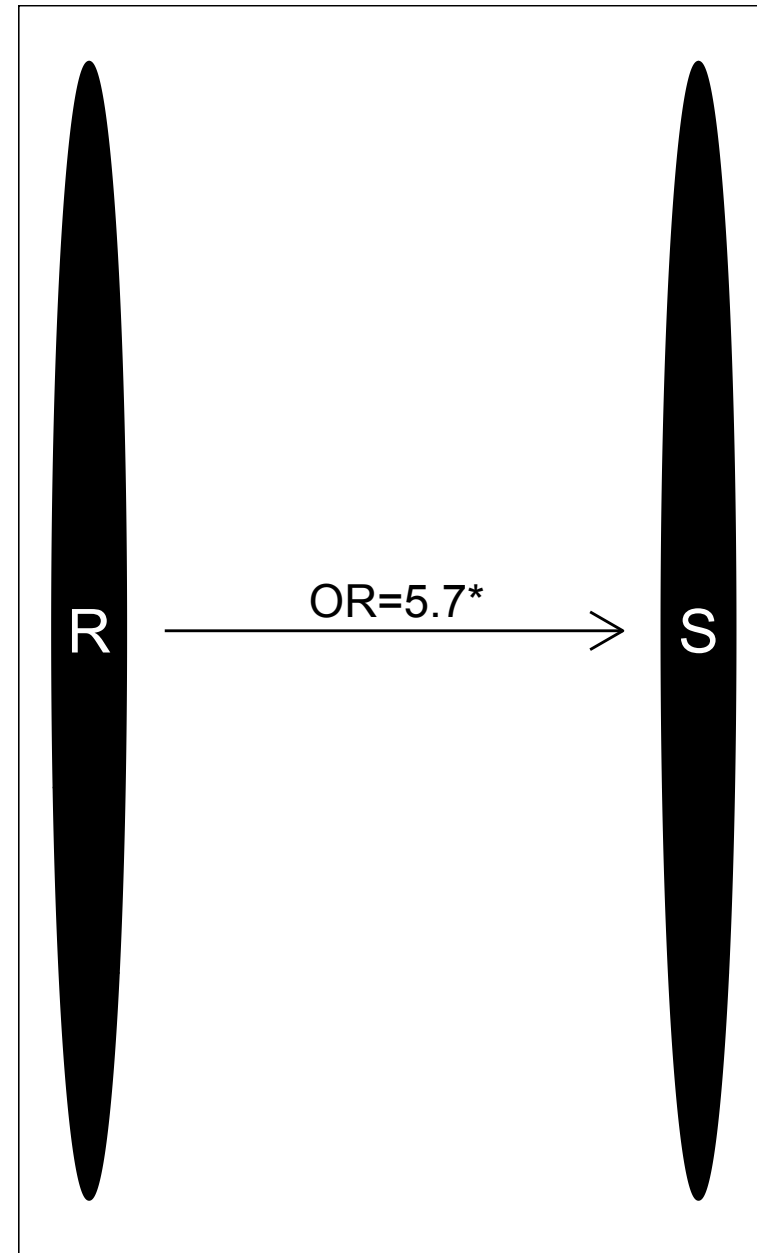

Supplement: Supplementary file 1 — Additional file 1: Table S1. List of insecticides tested [file 40249_2026_1458_MOESM1_ESM.pdf]

G454A-Cyp9K1

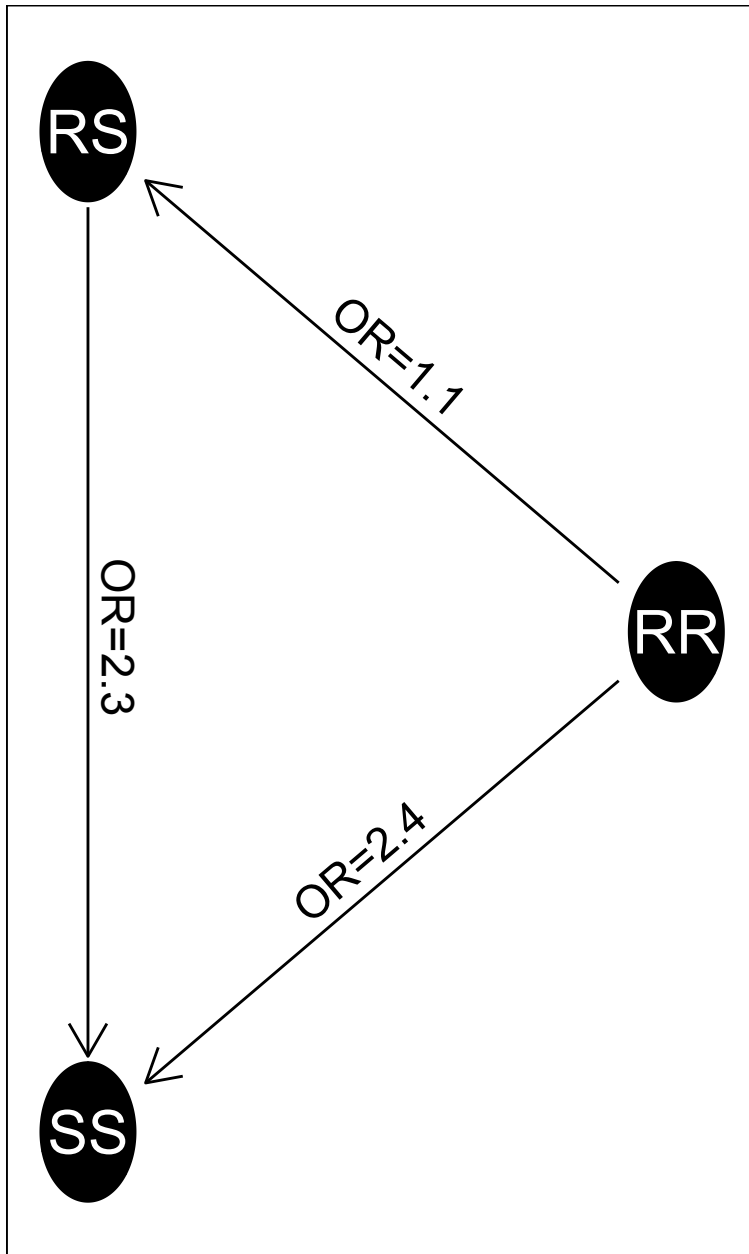

4.3 Kb-SV

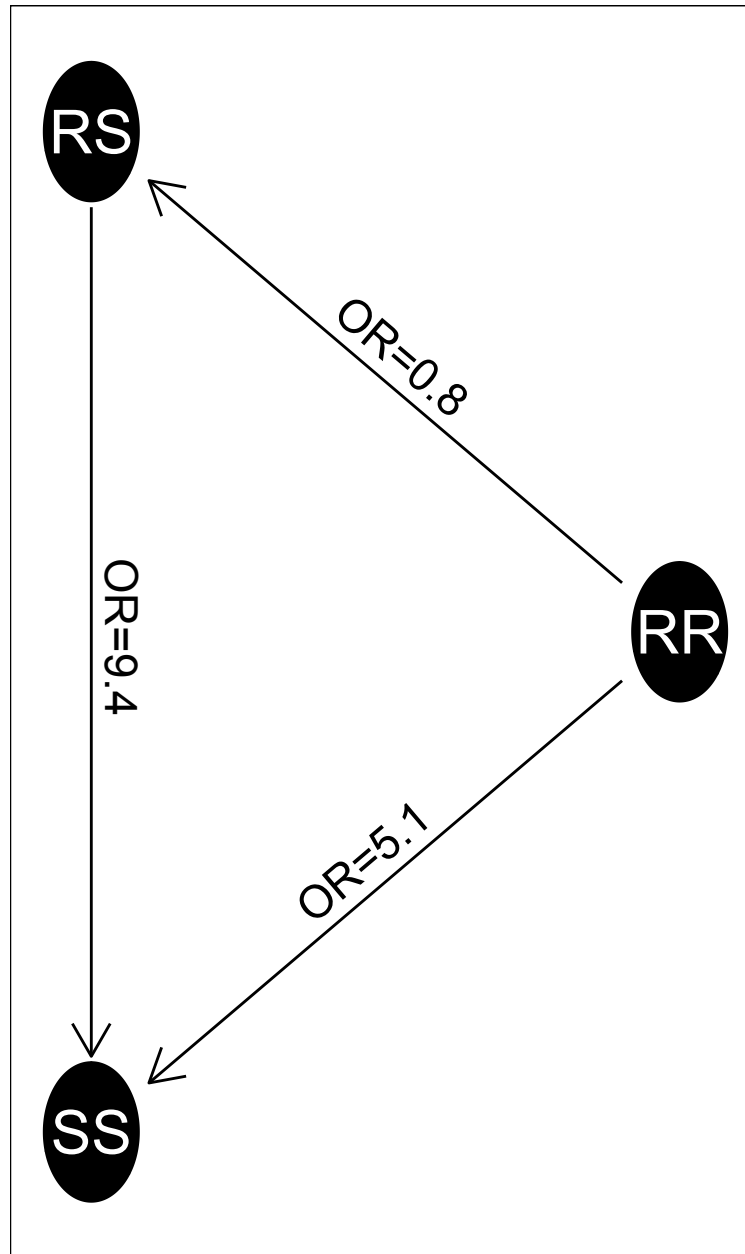

L119F-GSTe2

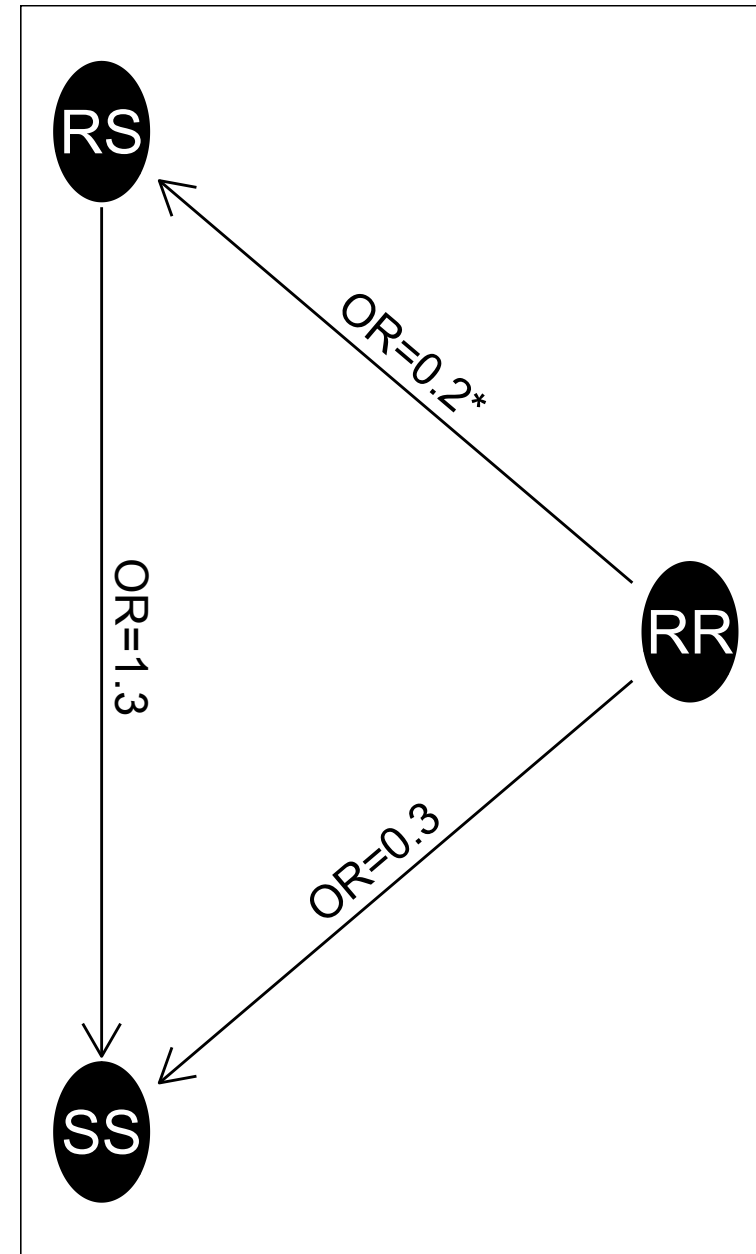

Supplement: Supplementary file 2 — Additional file 2: Table S2. Sequences of GSTe2 haplotypes analysed from Anopheles funestus in Kenya and other African countries. [file 40249_2026_1458_MOESM2_ESM.pdf]

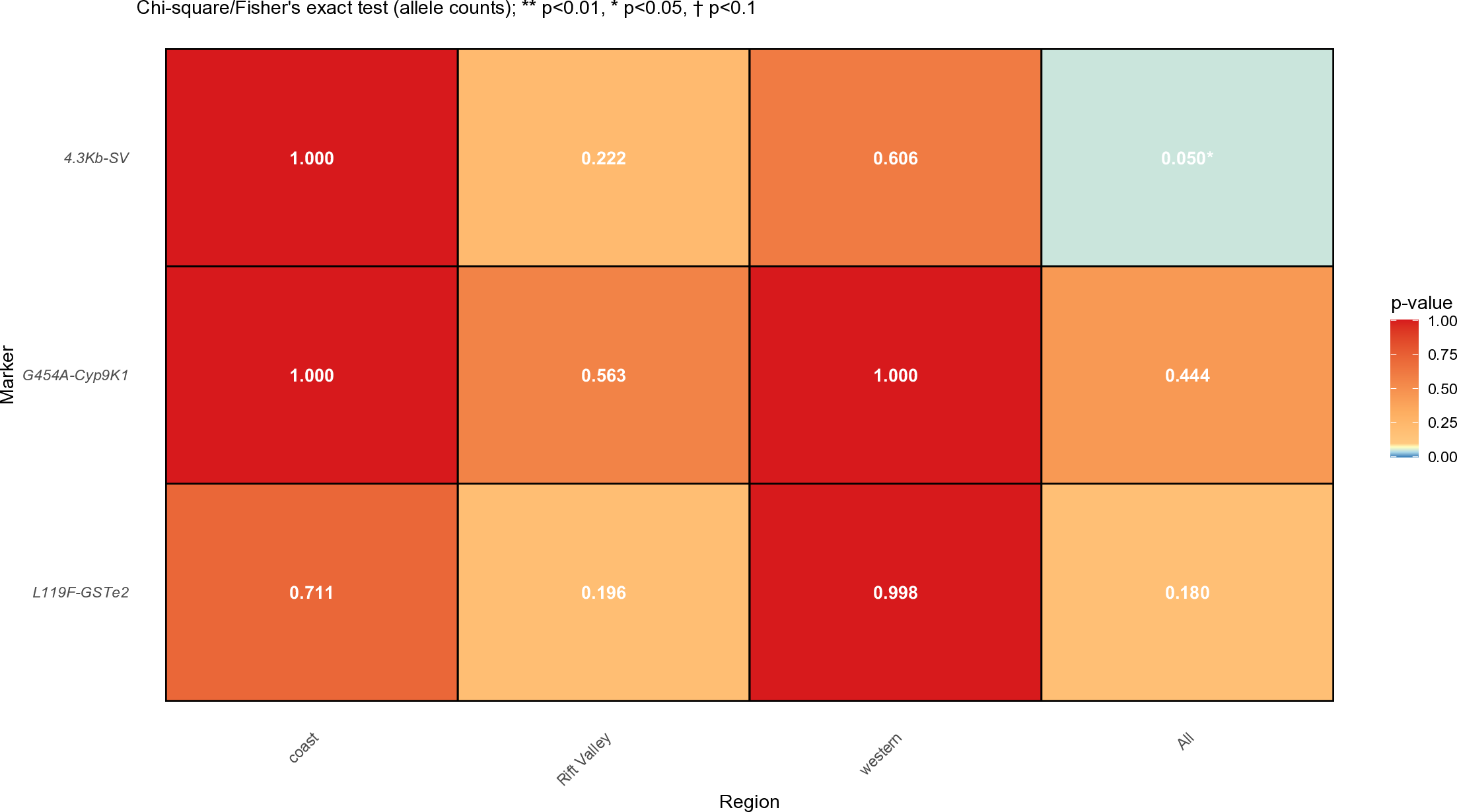

Supplement: Supplementary file 3 — Additional file 3: Fig. S1. Odds ratio versus genotype comparisons among Anopheles funestus mosquitoes infected with Plasmodium sporozoites. [file 40249_2026_1458_MOESM3_ESM.tif]
