# Supplementary material for: Signature of resistance gene evolution and pyrethroid resistance escalation in the major malaria vector Anopheles funestus across Kenyan malaria-endemic regions separated by the Rift Valley
Source: Infect Dis Poverty. 2026 May 15;15:57. doi: 10.1186/s40249-026-01458-1 (PMC13179624; doi:10.1186/s40249-026-01458-1)
Supplement: Supplementary file 5 — Additional file 5: Fig. S3. Summary statistics of allele-infection associations for the different markers in Anopheles funestus populations from Kenya. [file 40249_2026_1458_MOESM5_ESM.docx]

>MAL10-1

ATGACCAAGCTAGTTCTGTACACGCTACACCTAAGCCCACCATGCCGGGCCGTCGAACTG

ACAGCCAAAGCGTTGGGATTGGAACTGGAACAGAAGAATATTAACCTTCTGGCTGGTGAT

CATTTGACGCCGGAGTTCATGAAGTTAAACCCCCAACATACGATCCCGGTGCTGGATGAT

GATGGTACGATCATTACCGAGAGTCATGCGATCATGATCTATCTGGTGACGAAGTATGGC

AAAGATGACACCCTGTACCCAAAAGATCCAGTCCAGCAGGCTCGCGTAAATGCTGCCCTA

CACTTTGAATCTGGTGTACTGTTTGCACGAATGCGTTTCATTTTTGAGCGTATTCTTTTC

TACGGAAAATCGGACATTCCCGAAGATCGAGTCGAGTATGTGCAGAAATCGTACCGCTTG

CTGGAGGACACCCTAAAGGATGACTTTGTTGCTGGGTCGAAAATGACAATTGCCGACTTT

AGCTGCATTTCTACCATCTCTAGCATTATGGGCGTTGTTCCGCTGGAGCAATCGGAGCAT

CCACGTATCTATGAGTGGATCGATCGTTTGAAGCAGTTGCCATACTACGAGGAAGCTAAT

GGAGGCGGTGGAACTGACCTGGGCAAGTTTGTACTTGCCAAAAAGGAGGAAAATGCTAAA

GCTTGA

>MAL10-2

ATGACCAAGCTAGTTCTGTACACGCTACACCTAAGCCCACCATGCCGGGCCGTCGAACTG

ACAGCCAAAGCGTTGGGATTGGAACTGGAACAGAAGAATATTAACCTTCTGGCTGGTGAT

CATTTGACGCCGGAGTTCATGAAGTTAAACCCCCAACATACGATCCCGGTGCTGGATGAT

GATGGTACGATCATTACCGAGAGTCATGCGATCATGATCTATCTGGTGACGAAGTATGGC

AAAGATGACACCCTGTACCCAAAAGATCCAGTCCAGCAGGCTCGCGTAAATGCTGCCCTA

CACTTTGAATCTGGTGTACTGTTTGCACGAATGCGTTTCATTTTTGAGCGTATTCTTTTC

TACGGAAAATCGGACATTCCCGAAGATCGAGTCGAGTATGTGCAGAAATCGTACCGCTTG

CTGGAGGACACCCTAAAGGATGACTTTGTTGCTGGGTCGAAAATGACAATTGCCGACTTT

AGCTGCATTTCTACCATCTCTAGCATTATGGGCGTTGTTCCGCTGGAGCAATCGGAGCAT

CCACGTATCTATGAGTGGATCGATCGTTTGAAGCAGTTGCCATACTACGAGGAAGCTAAT

GGAGGCGGTGGAACTGACCTGGGCAAGTTTGTACTTGCCAAAAAGGAGGAAAATGCTAAA

GCTTGA

>MAL14-1

ATGACCAAGCTAGTTCTGTACACGCTACACCTAAGCCCACCATGCCGGGCCGTCGAACTG

ACAGCCAAAGCGTTGGGATTGGAACTGGAACAGAAGAATATTAACCTTCTGGCTGGTGAT

CATTTGACGCCGGAGTTCATGAAGTTAAACCCCCAACATACGATCCCGGTGCTGGATGAT

GATGGTACGATCATTACCGAGAGTCATGCGATCATGATCTATCTGGTGACGAAGTATGGC

AAAGATGACACCCTGTACCCAAAAGATCCAGTCCAGCAGGCTCGCGTAAATGCTGCCCTA

CACTTTGAATCTGGTGTACTGTTTGCACGAATGCGTTTCATTTTTGAGCGTATTCTTTTC

TACGGAAAATCGGACATTCCCGAAGATCGAGTCGAGTATGTGCAGAAATCGTACCGCTTG

CTGGAGGACACCCTAAAGGATGACTTTGTTGCTGGGTCGAAAATGACAATTGCCGACTTT

AGCTGCATTTCTACCATCTCTAGCATTATGGGCGTTGTTCCGCTGGAGCAATCGGAGCAT

CCACGTATCTATGAGTGGATCGATCGTTTGAAGCAGTTGCCATACTACGAGGAAGCTAAT

GGAGGCGGTGGAACTGACCTGGGCAAGTTTGTACTTGCCAAAAAGGAGGAAAATGCTAAA

GCTTGA

>MAL14-2

ATGACCAAGCTAGTTCTGTACACGCTACACCTAAGCCCACCATGCCGGGCCGTCGAACTG

ACAGCCAAAGCGTTGGGATTGGAACTGGAACAGAAGAATATTAACCTTCTGGCTGGTGAT

CATTTGACGCCGGAGTTCATGAAGTTAAACCCCCAACATACGATCCCGGTGCTGGATGAT

GATGGTACGATCATTACCGAGAGTCATGCGATCATGATCTATCTGGTGACGAAGTATGGC

AAAGATGACACCCTGTACCCAAAAGATCCAGTCCAGCAGGCTCGCGTAAATGCTGCCCTA

CACTTTGAATCTGGTGTACTGTTTGCACGAATGCGTTTCATTTTTGAGCGTATTCTTTTC

TACGGAAAATCGGACATTCCCGAAGATCGAGTCGAGTATGTGCAGAAATCGTACCGCTTG

CTGGAGGACACCCTAAAGGATGACTTTGTTGCTGGGTCGAAAATGACAATTGCCGACTTT

AGCTGCATTTCTACCATCTCTAGCATTATGGGCGTTGTTCCGCTGGAGCAATCGGAGCAT

CCACGTATCTATGAGTGGATCGATCGTTTGAAGCAGTTGCCATACTACGAGGAAGCTAAT

GGAGGCGGTGGAACTGACCTGGGCAAGTTTGTACTTGCCAAAAAGGAGGAAAATGCTAAA

GCTTGA

>MAL28-1

ATGACCAAGCTAGTTCTGTACACGCTACACCTAAGCCCACCATGCCGGGCCGTCGAACTG

ACAGCCAAAGCGTTGGGATTGGAACTGGAACAGAAGAATATTAACCTTCTGGCTGGTGAT

CATTTGACGCCGGAGTTCATGAAGTTAAACCCCCAACATACGATCCCGGTGCTGGATGAT

GATGGTACGATCATTACCGAGAGTCATGCGATCATGATCTATCTGGTGACGAAGTATGGC

AAAGATGACACCCTGTACCCAAAAGATCCAGTCCAGCAGGCTCGCGTAAATGCTGCCCTA

CACTTTGAATCTGGTGTACTGTTTGCACGAATGCGTTTCATTTTTGAGCGTATTCTTTTC

TACGGAAAATCGGACATTCCCGAAGATCGAGTCGAGTATGTGCAGAAATCGTACCGCTTG

CTGGAGGACACCCTAAAGGATGACTTTGTTGCTGGGTCGAAAATGACAATTGCCGACTTT

AGCTGCATTTCTACCATCTCTAGCATTATGGGCGTTGTTCCGCTGGAGCAATCGGAGCAT

CCACGTATCTATGAGTGGATCGATCGTTTGAAGCAGTTGCCATACTACGAGGAAGCTAAT

GGAGGCGGTGGAACTGACCTGGGCAAGTTTGTACTTGCCAAAAAGGAGGAAAATGCTAAA

GCTTGA

>MAL28-2

ATGACCAAGCTAGTTCTGTACACGCTACACCTAAGCCCACCATGCCGGGCCGTCGAACTG

ACAGCCAAAGCGTTGGGATTGGAACTGGAACAGAAGAATATTAACCTTCTGGCTGGTGAT

CATTTGACGCCGGAGTTCATGAAGTTAAACCCCCAACATACGATCCCGGTGCTGGATGAT

GATGGTACGATCATTACCGAGAGTCATGCGATCATGATCTATCTGGTGACGAAGTATGGC

AAAGATGACACCCTGTACCCAAAAGATCCAGTCCAGCAGGCTCGCGTAAATGCTGCCCTA

CACTTTGAATCTGGTGTACTGTTTGCACGAATGCGTTTCATTTTTGAGCGTATTCTTTTC

TACGGAAAATCGGACATTCCCGAAGATCGAGTCGAGTATGTGCAGAAATCGTACCGCTTG

CTGGAGGACACCCTAAAGGATGACTTTGTTGCTGGGTCGAAAATGACAATTGCCGACTTT

AGCTGCATTTCTACCATCTCTAGCATTATGGGCGTTGTTCCGCTGGAGCAATCGGAGCAT

CCACGTATCTATGAGTGGATCGATCGTTTGAAGCAGTTGCCATACTACGAGGAAGCTAAT

GGAGGCGGTGGAACTGACCTGGGCAAGTTTGTACTTGCCAAAAAGGAGGAAAATGCTAAA

GCTTGA

>MAL30-1

ATGACCAAGCTAGTTCTGTACACGCTACACCTAAGCCCACCATGCCGGGCCGTCGAACTG

ACAGCCAAAGCGTTGGGATTGGAACTGGAACAGAAGAATATTAACCTTCTGGCTGGTGAT

CATTTGACGCCGGAGTTCATGAAGTTAAACCCCCAACATACGATCCCGGTGCTGGATGAT

GATGGTACGATCATTACCGAGAGTCATGCGATCATGATCTATCTGGTGACGAAGTATGGC

AAAGATGACACCCTGTACCCAAAAGATCCAGTCCAGCAGGCTCGCGTAAATGCTGCCCTA

CACTTTGAATCTGGTGTACTGTTTGCACGAATGCGTTTCATTTTTGAGCGTATTCTTTTC

TACGGAAAATCGGACATTCCCGAAGATCGAGTCGAGTATGTGCAAAAATCGTACCGCTTG

CTGGAGGACACCCTAAAGGATGACTTTGTTGCTGGGTCGAAAATGACAATTGCCGACTTT

AGCTGCATTTCTACCATCTCTAGCATTATGGGCGTTGTTCCGCTGGAGCAATCGGAGCAT

CCACGTATCTATGAGTGGATCGATCGTTTGAAGCAGTTGCCATACTACGAGGAAGCTAAT

GGAGGCGGTGGAACTGACCTGGGCAAGTTTGTACTTGCCAAAAAGGAGGAAAATGCTAAA

GCTTGA

>MAL30-2

ATGACCAAGCTAGTTCTGTACACGCTACACCTAAGCCCACCATGCCGGGCCGTCGAACTG

ACAGCCAAAGCGTTGGGATTGGAACTGGAACAGAAGAATATTAACCTTCTGGCTGGTGAT

CATTTGACGCCGGAGTTCATGAAGTTAAACCCCCAACATACAATCCCGGTGCTGGATGAT

GATGGTACGATCATTACCGAGAGTCATGCGATCATGATCTATCTGGTGACGAAGTATGGC

AAAGATGACACCCTGTACCCAAAAGATCCAGTCCAGCAGGCTCGCGTAAATGCTGCCCTA

CACTTTGAATCTGGTGTACTGTTTGCACGAATGCGTTTCATTTTTGAGCGTATTCTTTTC

TACGGAAAATCGGACATTCCCGAAGATCGAGTCGAGTATGTGCAGAAATCGTACCGCTTG

CTGGAGGACACCCTAAAGGATGACTTTGTTGCTGGGTCGAAAATGACAATTGCCGACTTT

AGCTGCATTTCTACCATCTCTAGCATTATGGGCGTTGTTCCGCTGGAGCAATCGGAGCAT

CCACGTATCTATGAGTGGATCGATCGTTTGAAGCAGTTGCCATACTACGAGGAAGCTAAT

GGAGGCGGTGGAACTGACCTGGGCAAGTTTGTACTTGCCAAAAAGGAGGAAAATGCTAAA

GCTTGA

>MAL41-1

ATGACCAAGCTAGTTCTGTACACGCTACACCTAAGCCCACCATGCCGGGCCGTCGAACTG

ACAGCCAAAGCGTTGGGATTGGAACTGGAACAGAAGAATATTAACCTTCTGGCTGGTGAT

CATTTGACGCCGGAGTTCATGAAGTTAAACCCCCAACATACAATCCCGGTGCTGGATGAT

GATGGTACGATCATTACCGAGAGTCATGCGATCATGATCTATCTGGTGACGAAGTATGGC

AAAGATGACACCCTGTACCCAAAAGATCCAGTCCAGCAGGCTCGCGTAAATGCTGCCCTA

CACTTTGAATCTGGTGTACTGTTTGCACGAATGCGTTTCATTTTTGAGCGTATTCTTTTC

TACGGAAAATCGGACATTCCCGAAGATCGAGTCGAGTATGTGCAGAAATCGTACCGCTTG

CTGGAGGACACCCTAAAGGATGACTTTGTTGCTGGGTCGAAAATGACAATTGCCGACTTT

AGCTGCATTTCTACCATCTCTAGCATTATGGGCGTTGTTCCGCTGGAGCAATCGGAGCAT

CCACGTATCTATGAGTGGATCGATCGTTTGAAGCAGTTGCCATACTACGAGGAAGCTAAT

GGAGGCGGTGGAACTGACCTGGGCAAGTTTGTACTTGCCAAAAAGGAGGAAAATGCTAAA

GCTTGA

>MAL41-2

ATGACCAAGCTAGTTCTGTACACGCTACACCTAAGCCCACCATGCCGGGCCGTCGAACTG

ACAGCCAAAGCGTTGGGATTGGAACTGGAACAGAAGAATATTAACCTTCTGGCTGGTGAT

CATTTGACGCCGGAGTTCATGAAGTTAAACCCCCAACATACAATCCCGGTGCTGGATGAT

GATGGTACGATCATTACCGAGAGTCATGCGATCATGATCTATCTGGTGACGAAGTATGGC

AAAGATGACACCCTGTACCCAAAAGATCCAGTCCAGCAGGCTCGCGTAAATGCTGCCCTA

CACTTTGAATCTGGTGTACTGTTTGCACGAATGCGTTTCATTTTTGAGCGTATTCTTTTC

TACGGAAAATCGGACATTCCCGAAGATCGAGTCGAGTATGTGCAGAAATCGTACCGCTTG

CTGGAGGACACCCTAAAGGATGACTTTGTTGCTGGGTCGAAAATGACAATTGCCGACTTT

AGCTGCATTTCTACCATCTCTAGCATTATGGGCGTTGTTCCGCTGGAGCAATCGGAGCAT

CCACGTATCTATGAGTGGATCGATCGTTTGAAGCAGTTGCCATACTACGAGGAAGCTAAT

GGAGGCGGTGGAACTGACCTGGGCAAGTTTGTACTTGCCAAAAAGGAGGAAAATGCTAAA

GCTTGA

>MOZ9-1

ATGACCAAGCTAGTTCTGTACACGCTACACCTAAGCCCACCATGCCGGGCCGTCGAACTG

ACAGCCAAAGCGTTGGGATTGGAACTGGAACAGAAGAATATTAACCTTCTGGCTGGTGAT

CATTTGACGCCGGAGTTCATGAAGTTAAACCCCCAACATACGATCCCGGTGCTGGATGAT

GATGGTACGATCATTACCGAGAGTCATGCGATCATGATCTATCTGGTGACGAAGTATGGC

AAAGATGACACCCTGTACCCAAAAGATCCAGTCCAGCAGGCTCGCGTAAATGCTGCCCTA

CACTTTGAATCTGGTGTACTGTTTGCACGAATGCGTTTCATTTTTGAGCGTATTCTTTTC

TACGGAAAATCGGACATTCCCGAAGATCGAGTCGAGTATGTGCAGAAATCGTACCGCTTG

CTGGAGGACACCCTAAAGGATGACTTTGTTGCTGGGTCGAAAATGACAATTGCCGACTTT

AGCTGCATTTCTACCATCTCTAGCATTATGGGCGTTGTTCCGCTGGAGCAATCGGAGCAT

CCACGTATCTATGAGTGGATCGATCGTTTGAAGCAGTTGCCATACTACGAGGAAGCTAAT

GGAGGCGGTGGAACTGACCTGGGCAAGTTTGTACTTGCCAAAAAGGAGGAAAATGCTAAA

GCTTGA

>MOZ9-2

ATGACCAAGCTAGTTCTGTACACGCTACACCTAAGCCCACCATGCCGGGCCGTCGAACTG

ACAGCCAAAGCGTTGGGATTGGAACTGGAACAGAAGAATATTAACCTTCTGGCTGGTGAT

CATTTGACGCCGGAGTTCATGAAGTTAAACCCCCAACATACGATCCCGGTGCTGGATGAT

GATGGTACGATCATTACCGAGAGTCATGCGATCATGATCTATCTGGTGACGAAGTATGGC

AAAGATGACACCCTGTACCCAAAAGATCCAGTCCAGCAGGCTCGCGTAAATGCTGCCCTA

CACTTTGAATCTGGTGTACTGTTTGCACGAATGCGTTTCATTTTTGAGCGTATTCTTTTC

TACGGAAAATCGGACATTCCCGAAGATCGAGTCGAGTATGTGCAAAAATCGTACCGCTTG

CTGGAGGACACCCTAAAGGATGACTTTGTTGCTGGGTCGAAAATGACAATTGCCGACTTT

AGCTGCATTTCTACCATCTCTAGCATTATGGGCGTTGTTCCGCTGGAGCAATCGGAGCAT

CCACGTATCTATGAGTGGATCGATCGTTTGAAGCAGTTGCCATACTACGAGGAAGCTAAT

GGAGGCGGTGGAACTGACCTGGGCAAGTTTGTACTTGCCAAAAAGGAGGAAAATGCTAAA

GCTTGA

>MOZ15-1

ATGACCAAGCTAGTTCTGTACACGCTACACCTAAGCCCACCATGCCGGGCCGTCGAACTG

ACAGCCAAAGCGTTGGGATTGGAACTGGAACAGAAGAATATTAACCTTCTGGCTGGTGAT

CATTTGACGCCGGAGTTCATGAAGTTAAACCCCCAACATACGATCCCGGTGCTGGATGAT

GATGGTACGATCATTACCGAGAGTCATGCGATCATGATCTATCTGGTGACGAAGTATGGC

AAAGATGACACCCTGTACCCAAAAGATCCAGTCCAGCAGGCTCGCGTAAATGCTGCCCTA

CACTTTGAATCTGGTGTACTGTTTGCACGAATGCGTTTCATTTTTGAGCGTATTCTTTTC

TACGGAAAATCGGACATTCCCGAAGATCGAGTCGAGTATGTGCAGAAATCGTACCGCTTG

CTGGAGGACACCCTAAAGGATGACTTTGTTGCTGGGTCGAAAATGACAATTGCCGACTTT

AGCTGCATTTCTACCATCTCTAGCATTATGGGCGTTGTTCCGCTGGAGCAATCGGAGCAT

CCACGTATCTATGAGTGGATCGATCGTTTGAAGCAGTTGCCATACTACGAGGAAGCTAAT

GGAGGCGGTGGAACTGACCTGGGCAAGTTTGTACTTGCCAAAAAGGAGGAAAATGCTAAA

GCTTGA

>MOZ15-2

ATGACCAAGCTAGTTCTGTACACGCTACACCTAAGCCCACCATGCCGGGCCGTCGAACTG

ACAGCCAAAGCGTTGGGATTGGAACTGGAACAGAAGAATATTAACCTTCTGGCTGGTGAT

CATTTGACGCCGGAGTTCATGAAGTTAAACCCCCAACATACAATCCCGGTGCTGGATGAT

GATGGTACGATCATTACCGAGAGTCATGCGATCATGATCTATCTGGTGACGAAGTATGGC

AAAGATGACACCCTGTACCCAAAAGATCCAGTCCAGCAGGCTCGCGTAAATGCTGCCCTA

CACTTTGAATCTGGTGTACTGTTTGCACGAATGCGTTTCATTTTTGAGCGTATTCTTTTC

TACGGAAAATCGGACATTCCCGAAGATCGAGTCGAGTATGTGCAGAAATCGTACCGCTTG

CTGGAGGACACCCTAAAGGATGACTTTGTTGCTGGGTCGAAAATGACAATTGCCGACTTT

AGCTGCATTTCTACCATCTCTAGCATTATGGGCGTTGTTCCGCTGGAGCAATCGGAGCAT

CCACGTATCTATGAGTGGATCGATCGTTTGAAGCAGTTGCCATACTACGAGGAAGCTAAT

GGAGGCGGTGGAACTGACCTGGGCAAGTTTGTACTTGCCAAAAAGGAGGAAAATGCTAAA

GCTTGA

>MOZ25-1

ATGACCAAGCTAGTTCTGTACACGCTACACCTAAGCCCACCATGCCGGGCCGTCGAACTG

ACAGCCAAAGCGTTGGGATTGGAACTGGAACAGAAGAATATTAACCTTCTGGCTGGTGAT

CATTTGACGCCGGAGTTCATGAAGTTAAACCCCCAACATACGATCCCGGTGCTGGATGAT

GATGGTACGATCATTACCGAGAGTCATGCGATCATGATCTATCTGGTGACGAAGTATGGC

AAAGATGACACCCTGTACCCAAAAGATCCAGTCCAGCAGGCTCGCGTAAATGCTGCCCTA

CACTTTGAATCTGGTGTACTGTTTGCACGAATGCGTTTCATTTTTGAGCGTATTCTTTTC

TACGGAAAATCGGACATTCCCGAAGATCGAGTCGAGTATGTGCAGAAATCGTACCGCTTG

CTGGAGGACACCCTAAAGGATGACTTTGTTGCTGGGTCGAAAATGACAATTGCCGACTTT

AGCTGCATTTCTACCATCTCTAGCATTATGGGCGTTGTTCCGCTGGAGCAATCGGAGCAT

CCACGTATCTATGAGTGGATCGATCGTTTGAAGCAGTTGCCATACTACGAGGAAGCTAAT

GGAGGCGGTGGAACTGACCTGGGCAAGTTTGTACTTGCCAAAAAGGAGGAAAATGCTAAA

GCTTGA

>MOZ25-2

ATGACCAAGCTAGTTCTGTACACGCTACACCTAAGCCCACCATGCCGGGCCGTCGAACTG

ACAGCCAAAGCGTTGGGATTGGAACTGGAACAGAAGAATATTAACCTTCTGGCTGGTGAT

CATTTGACGCCGGAGTTCATGAAGTTAAACCCCCAACATACGATCCCGGTGCTGGATGAT

GATGGTACGATCATTACCGAGAGTCATGCGATCATGATCTATCTGGTGACGAAGTATGGC

AAAGATGACACCCTGTACCCAAAAGATCCAGTCCAGCAGGCTCGCGTAAATGCTGCCCTA

CACTTTGAATCTGGTGTACTGTTTGCACGAATGCGTTTCATTTTTGAGCGTATTCTTTTC

TACGGAAAATCGGACATTCCCGAAGATCGAGTCGAGTATGTGCAGAAATCGTACCGCTTG

CTGGAGGACACCCTAAAGGATGACTTTGTTGCTGGGTCGAAAATGACAATTGCCGACTTT

AGCTGCATTTCTACCATCTCTAGCATTATGGGCGTTGTTCCGCTGGAGCAATCGGAGCAT

CCACGTATCTATGAGTGGATCGATCGTTTGAAGCAGTTGCCATACTACGAGGAAGCTAAT

GGAGGCGGTGGAACTGACCTGGGCAAGTTTGTACTTGCCAAAAAGGAGGAAAATGCTAAA

GCTTGA

>UG10-1

ATGACCAAGCTAGTTCTGTACACGCTACACCTAAGCCCACCATGCCGGGCCGTCGAACTG

ACAGCCAAAGCGTTGGGATTGGAACTGGAACAGAAGAATATTAACCTTCTGGCTGGTGAT

CATTTGACGCCGGAGTTCATGAAGTTAAACCCCCAACATACGATCCCGGTGCTGGATGAT

GATGGTACGATCATTACCGAGAGTCATGCGATCATGATCTATCTGGTGACGAAGTATGGC

AAAGATGACACCCTGTACCCAAAAGATCCAGTCCAGCAGGCTCGCGTAAATGCTGCCCTA

CACTTTGAATCTGGTGTACTGTTTGCACGAATGCGTTTCATTTTTGAGCGTATTCTTTTC

TACGGAAAATCGGACATTCCCGAAGATCGAGTCGAGTATGTGCAGAAATCGTACCGCTTG

CTGGAGGACACCCTAAAGGATGACTTTGTTGCTGGGTCGAAAATGACAATTGCCGACTTT

AGCTGCATTTCTACCATCTCTAGCATTATGGGCGTTGTTCCGCTGGAGCAATCGGAGCAT

CCACGTATCTATGAGTGGATCGATCGTTTGAAGCAGTTGCCATACTACGAGGAAGCTAAT

GGAGGCGGTGGAACTGACCTGGGCAAGTTTGTACTTGCCAAAAAGGAGGAAAATGCTAAA

GCTTGA

>UG10-2

ATGACCAAGCTAGTTCTGTACACGCTACACCTAAGCCCACCATGCCGGGCCGTCGAACTG

ACAGCCAAAGCGTTGGGATTGGAACTGGAACAGAAGAATATTAACCTTCTGGCTGGTGAT

CATTTGACGCCGGAGTTCATGAAGTTAAACCCCCAACATACGATCCCGGTGCTGGATGAT

GATGGTACGATCATTACCGAGAGTCATGCGATCATGATCTATCTGGTGACGAAGTATGGC

AAAGATGACACCCTGTACCCAAAAGATCCAGTCCAGCAGGCTCGCGTAAATGCTGCCCTA

CACTTTGAATCTGGTGTACTGTTTGCACGAATGCGTTTCATTTTTGAGCGTATTTTTTTC

TACGGAAAATCGGACATTCCCGAAGATCGAGTCGAGTATGTGCAGAAATCGTACCGCTTG

CTGGAGGACACCCTAAAGGATGACTTTGTTGCTGGGTCGAAAATGACAATTGCCGACTTT

AGCTGCATTTCTACCATCTCTAGCATTATGGGCGTTGTTCCGCTGGAGCAATCGGAGCAT

CCACGTATCTATGAGTGGATCGATCGTTTGAAGCAGTTGCCATACTACGAGGAAGCTAAT

GGAGGCGGTGGAACTGACCTGGGCAAGTTTGTACTTGCCAAAAAGGAGGAAAATGCTAAA

GCTTGA

>UG28-1

ATGACCAAGCTAGTTCTGTACACGCTACACCTAAGCCCACCATGCCGGGCCGTCGAACTG

ACAGCCAAAGCGTTGGGATTGGAACTGGAACAGAAGAATATTAACCTTCTGGCTGGTGAG

CATTTGACGCCGGAGTTCATGAAGTTAAACCCCCAACATACGATCCCGGTGCTGGATGAT

GATGGTACGATCATTACCGAGAGTCATGCGATCATGATCTATCTGGTGACGAAGTATGGC

AAAGATGACACCCTGTACCCAAAAGATCCAGTCCAGCAGGCTCGCGTAAATGCTGCCCTA

CACTTTGAATCTGGTGTACTGTTTGCACGAATGCGTTTCATTTTTGAGCGTATTCTTTTC

TACGGAAAATCGGACATTCCCGAAGATCGAGTCGAGTATGTGCAGAAATCGTACCGCTTG

CTGGAGGACACCCTAAAGGATGACTTTGTTGCTGGGTCGAAAATGACAATTGCCGACTTT

AGCTGCATTTCTACCATCTCTAGCATTATGGGCGTTGTTCCGCTGGAGCAATCGGAGCAT

CCACGTATCTATGAGTGGATCGATCGTTTGAAGCAGTTGCCATACTACGAGGAAGCTAAT

GGAGGCGGTGGAACTGACCTGGGCAAGTTTGTACTTGCCAAAAAGGAGGAAAATGCTAAA

GCTTGA

>UG28-2

ATGACCAAGCTAGTTCTGTACACGCTACACCTAAGCCCACCATGCCGGGCCGTCGAACTG

ACAGCCAAAGCGTTGGGATTGGAACTGGAACAGAAGAATATTAACCTTCTGGCTGGTGAG

CATTTGACGCCGGAGTTCATGAAGTTAAACCCCCAACATACGATCCCGGTGCTGGATGAT

GATGGTACGATCATTACCGAGAGTCATGCGATCATGATCTATCTGGTGACGAAGTATGGC

AAAGATGACACCCTGTACCCAAAAGATCCAGTCCAGCAGGCTCGCGTAAATGCTGCCCTA

CACTTTGAATCTGGTGTACTGTTTGCACGAATGCGTTTCATTTTTGAGCGTATTCTTTTC

TACGGAAAATCGGACATTCCCGAAGATCGAGTCGAGTATGTGCAGAAATCGTACCGCTTG

CTGGAGGACACCCTAAAGGATGACTTTGTTGCTGGGTCGAAAATGACAATTGCCGACTTT

AGCTGCATTTCTACCATCTCTAGCATTATGGGCGTTGTTCCGCCGGAGCAATCGGAGCAT

CCACGTATCTATGAGTGGATCGATCGTTTGAAGCAGTTGCCATACTACGAGGAAGCTAAT

GGAGGCGGTGGAACTGACCTGGGCAAGTTTGTACTTGCCAAAAAGGAGGAAAATGCTAAA

GCTTGA

>UG32-1

ATGACCAAGCTAGTTCTGTACACGCTACACCTAAGCCCACCATGCCGGGCCGTCGAACTG

ACAGCCAAAGCGTTGGGATTGGAACTGGAACAGAAGAATATTAACCTTCTGGCTGGTGAT

CATTTGACGCCGGAGTTCATGAAGTTAAACCCCCAACATACGATCCCGGTGCTGGATGAT

GATGGTACGATCATTACCGAGAGTCATGCGATCATGATCTATCTGGTGACGAAGTATGGC

AAAGATGACACCCTGTACCCAAAAGATCCAGTCCAGCAGGCTCGCGTAAATGCTGCCCTA

CACTTTGAATCTGGTGTACTGTTTGCACGAATGCGTTTCATTTTTGAGCGTATTCTTTTC

TACGGAAAATCGGACATTCCCGAAGATCGAGTCGAGTATGTGCAGAAATCGTACCGCTTG

CTGGAGGACACCCTAAAGGATGACTTTGTTGCTGGGTCGAAAATGACAATTGCCGACTTT

AGCTGCATTTCTACCATCTCTAGCATTATGGGCGTTGTTCCGCTGGAGCAATCGGAGCAT

CCACGTATCTATGAGTGGATCGATCGTTTGAAGCAGTTGCCATACTACGAGGAAGCTAAT

GGAGGCGGTGGAACTGACCTGGGCAAGTTTGTACTTGCCAAAAAGGAGGAAAATGCTAAA

GCTTGA

>UG32-2

ATGACCAAGCTAGTTCTGTACACGCTACACCTAAGCCCACCATGCCGGGCCGTCGAACTG

ACAGCCAAAGCGTTGGGATTGGAACTGGAACAGAAGAATATTAACCTTCTGGCTGGTGAG

CATTTGACGCCGGAGTTCATGAAGTTAAACCCCCAACATACGATCCCGGTGCTGGATGAT

GATGGTACGATCATTACCGAGAGTCATGCGATCATGATCTATCTGGTGACGAAGTATGGC

AAAGATGACACCCTGTACCCAAAAGATCCAGTCCAGCAGGCTCGCGTAAATGCTGCCCTA

CACTTTGAATCTGGTGTACTGTTTGCACGAATGCGTTTCATTTTTGAGCGTATTCTTTTC

TACGGAAAATCGGACATTCCCGAAGATCGAGTCGAGTATGTGCAGAAATCGTACCGCTTG

CTGGAGGACACCCTAAAGGATGACTTTGTTGCTGGGTCGAAAATGACAATTGCCGACTTT

AGCTGCATTTCTACCATCTCTAGCATTATGGGCGTTGTTCCGCTGGAGCAATCGGAGCAT

CCACGTATCTATGAGTGGATCGATCGTTTGAAGCAGTTGCCATACTACGAGGAAGCTAAT

GGAGGCGGTGGAACTGACCTGGGCAAGTTTGTACTTGCCAAAAAGGAGGAAAATGCTAAA

GCTTGA

>UG33-1

ATGACCAAGCTAGTTCTGTACACGCTACACCTAAGCCCACCATGCCGGGCCGTCGAACTG

ACAGCCAAAGCGTTGGGATTGGAACTGGAACAGAAGAATATTAACCTTCTGGCTGGTGAT

CATTTGACGCCGGAGTTCATGAAGTTAAACCCCCAACATACGATCCCGGTGCTGGATGAT

GATGGTACGATCATTACCGAGAGTCATGCGATCATGATCTATCTGGTGACGAAGTATGGC

AAAGATGACACCCTGTACCCAAAAGATCCAGTCCAGCAGGCTCGCGTAAATGCTGCCCTA

CACTTTGAATCTGGTGTACTGTTTGCACGAATGCGTTTCATTTTTGAGCGTATTCTTTTC

TACGGAAAATCGGACATTCCCGAAGATCGAGTCGAGTATGTGCAGAAATCGTACCGCTTG

CTGGAGGACACCCTAAAGGATGACTTTGTTGCTGGGTCGAAAATGACAATTGCCGACTTT

AGCTGCATTTCTACCATCTCTAGCATTATGGGCGTTGTTCCGCTGGAGCAATCGGAGCAT

CCACGTATCTATGAGTGGATCGATCGTTTGAAGCAGTTGCCATACTACGAGGAAGCTAAT

GGAGGCGGTGGAACTGACCTGGGCAAGTTTGTACTTGCCAAAAAGGAGGAAAATGCTAAA

GCTTGA

>UG33-2

ATGACCAAGCTAGTTCTGTACACGCTACACCTAAGCCCACCATGCCGGGCCGTCGAACTG

ACAGCCAAAGCGTTGGGATTGGAACTGGAACAGAAGAATATTAACCTTCTGGCTGGTGAT

CATTTGACGCCGGAGTTCATGAAGTTAAACCCCCAACATACGATCCCGGTGCTGGATGAT

GATGGTACGATCATTACCGAGAGTCATGCGATCATGATCTATCTGGTGACGAAGTATGGC

AAAGATGACACCCTGTACCCAAAAGATCCAGTCCAGCAGGCTCGCGTAAATGCTGCCCTA

CACTTTGAATCTGGTGTACTGTTTGCACGAATGCGTTTCATTTTTGAGCGTATTCTTTTC

TACGGAAAATCGGACATTCCCGAAGATCGAGTCGAGTATGTGCAGAAATCGTACCGCTTG

CTGGAGGACACCCTAAAGGATGACTTTGTTGCTGGGTCGAAAATGACAATTGCCGACTTT

AGCTGCATTTCTACCATCTCTAGCATTATGGGCGTTGTTCCGCTGGAGCAATCGGAGCAT

CCACGTATCTATGAGTGGATCGATCGTTTGAAGCAGTTGCCATACTACGAGGAAGCTAAT

GGAGGCGGTGGAACTGACCTGGGCAAGTTTGTACTTGCCAAAAAGGAGGAAAATGCTAAA

GCTTGA

>BN27-1

ATGACCAAGCTAGTTCTGTACACGCTACACCTAAGCCCACCATGCCGGGCCGTCGAACTG

ACAGCCAAAGCGTTGGGATTGGAACTGGAACAGAAGAATATTAACCTTCTGGCGGGTGAT

CATTTGACGCCGGAGTTCATGAAGTTAAACCCCCAACATACGATCCCGGTGCTGGATGAT

GATGGTACGATCATTACCGAGAGTCATGCGATCATGATCTATCTGGTGACGAAGTATGGC

AAAGATGACACCCTGTACCCAAAAGATCCAGTCCAGCAGGCTCGCGTAAATGCTGCCCTA

CACTTTGAATCTGGTGTACTGTTTGCACGAATGCGTTTCATTTTTGAGCGTATTTTTTTC

TACGGAAAATCGGACATTCCCGAAGATCGAGTCGAGTATGTGCAGAAATCGTACCGCTTG

CTGGAGGACACCCTAAAGGATGACTTTGTTGCTGGGTCGAAAATGACAATTGCCGACTTT

AGCTGCATTTCTACCATCTCTAGCATTATGGGCGTTGTTCCGCTGGAGCAATCGGAGCAT

CCACGTATCTATGAGTGGATCGATCGTTTGAAGCAGTTGCCATACTACGAGGAAGCTAAT

GGAGGCGGTGGAACTGACCTGGGCAAGTTTGTACTTGCCAAAAAGGAGGAAAATGCTAAA

GCTTGA

>BN27-2

ATGACCAAGCTAGTTCTGTACACGCTACACCTAAGCCCACCATGCCGGGCCGTCGAACTG

ACAGCCAAAGCGTTGGGATTGGAACTGGAACAGAAGAATATTAACCTTCTGGCGGGTGAT

CATTTGACGCCGGAGTTCATGAAGTTAAACCCCCAACATACGATCCCGGTGCTGGATGAT

GATGGTACGATCATTACCGAGAGTCATGCGATCATGATCTATCTGGTGACGAAGTATGGC

AAAGATGACACCCTGTACCCAAAAGATCCAGTCCAGCAGGCTCGCGTAAATGCTGCCCTA

CACTTTGAATCTGGTGTACTGTTTGCACGAATGCGTTTCATTTTTGAGCGTATTTTTTTC

TACGGAAAATCGGACATTCCCGAAGATCGAGTCGAGTATGTGCAGAAATCGTACCGCTTG

CTGGAGGACACCCTAAAGGATGACTTTGTTGCTGGGTCGAAAATGACAATTGCCGACTTT

AGCTGCATTTCTACCATCTCTAGCATTATGGGCGTTGTTCCGCTGGAGCAATCGGAGCAT

CCACGTATCTATGAGTGGATCGATCGTTTGAAGCAGTTGCCATACTACGAGGAAGCTAAT

GGAGGCGGTGGAACTGACCTGGGCAAGTTTGTACTTGCCAAAAAGGAGGAAAATGCTAAA

GCTTGA

>BN10-1

ATGACCAAGCTAGTTCTGTACACGCTACACCTAAGCCCACCATGCCGGGCCGTCGAACTG

ACAGCCAAAGCGTTGGGATTGGAACTGGAACAGAAGAATATTAACCTTCTGGCGGGTGAT

CATTTGACGCCGGAGTTCATGAAGTTAAACCCCCAACATACGATCCCGGTGCTGGATGAT

GATGGTACGATCATTACCGAGAGTCATGCGATCATGATCTATCTGGTGACGAAGTATGGC

AAAGATGACACCCTGTACCCAAAAGATCCAGTCCAGCAGGCTCGCGTAAATGCTGCCCTA

CACTTTGAATCTGGTGTACTGTTTGCACGAATGCGTTTCATTTTTGAGCGTATTTTTTTC

TACGGAAAATCGGACATTCCCGAAGATCGAGTCGAGTATGTGCAGAAATCGTACCGCTTG

CTGGAGGACACCCTAAAGGATGACTTTGTTGCTGGGTCGAAAATGACAATTGCCGACTTT

AGCTGCATTTCTACCATCTCTAGCATTATGGGCGTTGTTCCGCTGGAGCAATCGGAGCAT

CCACGTATCTATGAGTGGATCGATCGTTTGAAGCAGTTGCCATACTACGAGGAAGCTAAT

GGAGGCGGTGGAACTGACCTGGGCAAGTTTGTACTTGCCAAAAAGGAGGAAAATGCTAAA

GCTTGA

>BN10-2

ATGACCAAGCTAGTTCTGTACACGCTACACCTAAGCCCACCATGCCGGGCCGTCGAACTG

ACAGCCAAAGCGTTGGGATTGGAACTGGAACAGAAGAATATTAACCTTCTGGCGGGTGAT

CATTTGACGCCGGAGTTCATGAAGTTAAACCCCCAACATACGATCCCGGTGCTGGATGAT

GATGGTACGATCATTACCGAGAGTCATGCGATCATGATCTATCTGGTGACGAAGTATGGC

AAAGATGACACCCTGTACCCAAAAGATCCAGTCCAGCAGGCTCGCGTAAATGCTGCCCTA

CACTTTGAATCTGGTGTACTGTTTGCACGAATGCGTTTCATTTTTGAGCGTATTTTTTTC

TACGGAAAATCGGACATTCCCGAAGATCGAGTCGAGTATGTGCAGAAATCGTACCGCTTG

CTGGAGGACACCCTAAAGGATGACTTTGTTGCTGGGTCGAAAATGACAATTGCCGACTTT

AGCTGCATTTCTACCATCTCTAGCATTATGGGCGTTGTTCCGCTGGAGCAATCGGAGCAT

CCACGTATCTATGAGTGGATCGATCGTTTGAAGCAGTTGCCATACTACGAGGAAGCTAAT

GGAGGCGGTGGAACTGACCTGGGCAAGTTTGTACTTGCCAAAAAGGAGGAAAATGCTAAA

GCTTGA

>BN11-1

ATGACCAAGCTAGTTCTGTACACGCTACACCTAAGCCCACCATGCCGGGCCGTCGAACTG

ACAGCCAAAGCGTTGGGATTGGAACTGGAACAGAAGAATATTAACCTTCTGGCGGGTGAT

CATTTGACGCCGGAGTTCATGAAGTTAAACCCCCAACATACGATCCCGGTGCTGGATGAT

GATGGTACGATCATTACCGAGAGTCATGCGATCATGATCTATCTGGTGACGAAGTATGGC

AAAGATGACACCCTGTACCCAAAAGATCCAGTCCAGCAGGCTCGCGTAAATGCTGCCCTA

CACTTTGAATCTGGTGTACTGTTTGCACGAATGCGTTTCATTTTTGAGCGTATTTTTTTC

TACGGAAAATCGGACATTCCCGAAGATCGAGTCGAGTATGTGCAGAAATCGTACCGCTTG

CTGGAGGACACCCTAAAGGATGACTTTGTTGCTGGGTCGAAAATGACAATTGCCGACTTT

AGCTGCATTTCTACCATCTCTAGCATTATGGGCGTTGTTCCGCTGGAGCAATCGGAGCAT

CCACGTATCTATGAGTGGATCGATCGTTTGAAGCAGTTGCCATACTACGAGGAAGCTAAT

GGAGGCGGTGGAACTGACCTGGGCAAGTTTGTACTTGCCAAAAAGGAGGAAAATGCTAAA

GCTTGA

>BN11-2

ATGACCAAGCTAGTTCTGTACACGCTACACCTAAGCCCACCATGCCGGGCCGTCGAACTG

ACAGCCAAAGCGTTGGGATTGGAACTGGAACAGAAGAATATTAACCTTCTGGCGGGTGAT

CATTTGACGCCGGAGTTCATGAAGTTAAACCCCCAACATACGATCCCGGTGCTGGATGAT

GATGGTACGATCATTACCGAGAGTCATGCGATCATGATCTATCTGGTGACGAAGTATGGC

AAAGATGACACCCTGTACCCAAAAGATCCAGTCCAGCAGGCTCGCGTAAATGCTGCCCTA

CACTTTGAATCTGGTGTACTGTTTGCACGAATGCGTTTCATTTTTGAGCGTATTTTTTTC

TACGGAAAATCGGACATTCCCGAAGATCGAGTCGAGTATGTGCAGAAATCGTACCGCTTG

CTGGAGGACACCCTAAAGGATGACTTTGTTGCTGGGTCGAAAATGACAATTGCCGACTTT

AGCTGCATTTCTACCATCTCTAGCATTATGGGCGTTGTTCCGCTGGAGCAATCGGAGCAT

CCACGTATCTATGAGTGGATCGATCGTTTGAAGCAGTTGCCATACTACGAGGAAGCTAAT

GGAGGCGGTGGAACTGACCTGGGCAAGTTTGTACTTGCCAAAAAGGAGGAAAATGCTAAA

GCTTGA

>BN14-1

ATGACCAAGCTAGTTCTGTACACGCTACACCTAAGCCCACCATGCCGGGCCGTCGAACTG

ACAGCCAAAGCGTTGGGATTGGAACTGGAACAGAAGAATATTAACCTTCTGGCGGGTGAT

CATTTGACGCCGGAGTTCATGAAGTTAAACCCCCAACATACGATCCCGGTGCTGGATGAT

GATGGTACGATCATTACCGAGAGTCATGCGATCATGATCTATCTGGTGACGAAGTATGGC

AAAGATGACACCCTGTACCCAAAAGATCCAGTCCAGCAGGCTCGCGTAAATGCTGCCCTA

CACTTTGAATCTGGTGTACTGTTTGCACGAATGCGTTTCATTTTTGAGCGTATTTTTTTC

TACGGAAAATCGGACATTCCCGAAGATCGAGTCGAGTATGTGCAGAAATCGTACCGCTTG

CTGGAGGACACCCTAAAGGATGACTTTGTTGCTGGGTCGAAAATGACAATTGCCGACTTT

AGCTGCATTTCTACCATCTCTAGCATTATGGGCGTTGTTCCGCTGGAGCAATCGGAGCAT

CCACGTATCTATGAGTGGATCGATCGTTTGAAGCAGTTGCCATACTACGAGGAAGCTAAT

GGAGGCGGTGGAACTGACCTGGGCAAGTTTGTACTTGCCAAAAAGGAGGAAAATGCTAAA

GCTTGA

>BN14-2

ATGACCAAGCTAGTTCTGTACACGCTACACCTAAGCCCACCATGCCGGGCCGTCGAACTG

ACAGCCAAAGCGTTGGGATTGGAACTGGAACAGAAGAATATTAACCTTCTGGCGGGTGAT

CATTTGACGCCGGAGTTCATGAAGTTAAACCCCCAACATACGATCCCGGTGCTGGATGAT

GATGGTACGATCATTACCGAGAGTCATGCGATCATGATCTATCTGGTGACGAAGTATGGC

AAAGATGACACCCTGTACCCAAAAGATCCAGTCCAGCAGGCTCGCGTAAATGCTGCCCTA

CACTTTGAATCTGGTGTACTGTTTGCACGAATGCGTTTCATTTTTGAGCGTATTTTTTTC

TACGGAAAATCGGACATTCCCGAAGATCGAGTCGAGTATGTGCAGAAATCGTACCGCTTG

CTGGAGGACACCCTAAAGGATGACTTTGTTGCTGGGTCGAAAATGACAATTGCCGACTTT

AGCTGCATTTCTACCATCTCTAGCATTATGGGCGTTGTTCCGCTGGAGCAATCGGAGCAT

CCACGTATCTATGAGTGGATCGATCGTTTGAAGCAGTTGCCATACTACGAGGAAGCTAAT

GGAGGCGGTGGAACTGACCTGGGCAAGTTTGTACTTGCCAAAAAGGAGGAAAATGCTAAA

GCTTGA

>BN21-1

ATGACCAAGCTAGTTCTGTACACGCTACACCTAAGCCCACCATGCCGGGCCGTCGAACTG

ACAGCCAAAGCGTTGGGATTGGAACTGGAACAGAAGAATATTAACCTTCTGGCGGGTGAT

CATTTGACGCCGGAGTTCATGAAGTTAAACCCCCAACATACGATCCCGGTGCTGGATGAT

GATGGTACGATCATTACCGAGAGTCATGCGATCATGATCTATCTGGTGACGAAGTATGGC

AAAGATGACACCCTGTACCCAAAAGATCCAGTCCAGCAGGCTCGCGTAAATGCTGCCCTA

CACTTTGAATCTGGTGTACTGTTTGCACGAATGCGTTTCATTTTTGAGCGTATTTTTTTC

TACGGAAAATCGGACATTCCCGAAGATCGAGTCGAGTATGTGCAGAAATCGTACCGCTTG

CTGGAGGACACCCTAAAGGATGACTTTGTTGCTGGGTCGAAAATGACAATTGCCGACTTT

AGCTGCATTTCTACCATCTCTAGCATTATGGGCGTTGTTCCGCTGGAGCAATCGGAGCAT

CCACGTATCTATGAGTGGATCGATCGTTTGAAGCAGTTGCCATACTACGAGGAAGCTAAT

GGAGGCGGTGGAACTGACCTGGGCAAGTTTGTACTTGCCAAAAAGGAGGAAAATGCTAAA

GCTTGA

>BN21-2

ATGACCAAGCTAGTTCTGTACACGCTACACCTAAGCCCACCATGCCGGGCCGTCGAACTG

ACAGCCAAAGCGTTGGGATTGGAACTGGAACAGAAGAATATTAACCTTCTGGCGGGTGAT

CATTTGACGCCGGAGTTCATGAAGTTAAACCCCCAACATACGATCCCGGTGCTGGATGAT

GATGGTACGATCATTACCGAGAGTCATGCGATCATGATCTATCTGGTGACGAAGTATGGC

AAAGATGACACCCTGTACCCAAAAGATCCAGTCCAGCAGGCTCGCGTAAATGCTGCCCTA

CACTTTGAATCTGGTGTACTGTTTGCACGAATGCGTTTCATTTTTGAGCGTATTTTTTTC

TACGGAAAATCGGACATTCCCGAAGATCGAGTCGAGTATGTGCAGAAATCGTACCGCTTG

CTGGAGGACACCCTAAAGGATGACTTTGTTGCTGGGTCGAAAATGACAATTGCCGACTTT

AGCTGCATTTCTACCATCTCTAGCATTATGGGCGTTGTTCCGCTGGAGCAATCGGAGCAT

CCACGTATCTATGAGTGGATCGATCGTTTGAAGCAGTTGCCATACTACGAGGAAGCTAAT

GGAGGCGGTGGAACTGACCTGGGCAAGTTTGTACTTGCCAAAAAGGAGGAAAATGCTAAA

GCTTGA

>BN22-1

ATGACCAAGCTAGTTCTGTACACGCTACACCTAAGCCCACCATGCCGGGCCGTCGAACTG

ACAGCCAAAGCGTTGGGATTGGAACTGGAACAGAAGAATATTAACCTTCTGGCGGGTGAT

CATTTGACGCCGGAGTTCATGAAGTTAAACCCCCAACATACGATCCCGGTGCTGGATGAT

GATGGTACGATCATTACCGAGAGTCATGCGATCATGATCTATCTGGTGACGAAGTATGGC

AAAGATGACACCCTGTACCCAAAAGATCCAGTCCAGCAGGCTCGCGTAAATGCTGCCCTA

CACTTTGAATCTGGTGTACTGTTTGCACGAATGCGTTTCATTTTTGAGCGTATTTTTTTC

TACGGAAAATCGGACATTCCCGAAGATCGAGTCGAGTATGTGCAGAAATCGTACCGCTTG

CTGGAGGACACCCTAAAGGATGACTTTGTTGCTGGGTCGAAAATGACAATTGCCGACTTT

AGCTGCATTTCTACCATCTCTAGCATTATGGGCGTTGTTCCGCTGGAGCAATCGGAGCAT

CCACGTATCTATGAGTGGATCGATCGTTTGAAGCAGTTGCCATACTACGAGGAAGCTAAT

GGAGGCGGTGGAACTGACCTGGGCAAGTTTGTACTTGCCAAAAAGGAGGAAAATGCTAAA

GCTTGA

>BN22-2

ATGACCAAGCTAGTTCTGTACACGCTACACCTAAGCCCACCATGCCGGGCCGTCGAACTG

ACAGCCAAAGCGTTGGGATTGGAACTGGAACAGAAGAATATTAACCTTCTGGCGGGTGAT

CATTTGACGCCGGAGTTCATGAAGTTAAACCCCCAACATACGATCCCGGTGCTGGATGAT

GATGGTACGATCATTACCGAGAGTCATGCGATCATGATCTATCTGGTGACGAAGTATGGC

AAAGATGACACCCTGTACCCAAAAGATCCAGTCCAGCAGGCTCGCGTAAATGCTGCCCTA

CACTTTGAATCTGGTGTACTGTTTGCACGAATGCGTTTCATTTTTGAGCGTATTTTTTTC

TACGGAAAATCGGACATTCCCGAAGATCGAGTCGAGTATGTGCAGAAATCGTACCGCTTG

CTGGAGGACACCCTAAAGGATGACTTTGTTGCTGGGTCGAAAATGACAATTGCCGACTTT

AGCTGCATTTCTACCATCTCTAGCATTATGGGCGTTGTTCCGCTGGAGCAATCGGAGCAT

CCACGTATCTATGAGTGGATCGATCGTTTGAAGCAGTTGCCATACTACGAGGAAGCTAAT

GGAGGCGGTGGAACTGACCTGGGCAAGTTTGTACTTGCCAAAAAGGAGGAAAATGCTAAA

GCTTGA

>BN25-1

ATGACCAAGCTAGTTCTGTACACGCTACACCTAAGCCCACCATGCCGGGCCGTCGAACTG

ACAGCCAAAGCGTTGGGATTGGAACTGGAACAGAAGAATATTAACCTTCTGGCGGGTGAT

CATTTGACGCCGGAGTTCATGAAGTTAAACCCCCAACATACGATCCCGGTGCTGGATGAT

GATGGTACGATCATTACCGAGAGTCATGCGATCATGATCTATCTGGTGACGAAGTATGGC

AAAGATGACACCCTGTACCCAAAAGATCCAGTCCAGCAGGCTCGCGTAAATGCTGCCCTA

CACTTTGAATCTGGTGTACTGTTTGCACGAATGCGTTTCATTTTTGAGCGTATTTTTTTC

TACGGAAAATCGGACATTCCCGAAGATCGAGTCGAGTATGTGCAGAAATCGTACCGCTTG

CTGGAGGACACCCTAAAGGATGACTTTGTTGCTGGGTCGAAAATGACAATTGCCGACTTT

AGCTGCATTTCTACCATCTCTAGCATTATGGGCGTTGTTCCGCTGGAGCAATCGGAGCAT

CCACGTATCTATGAGTGGATCGATCGTTTGAAGCAGTTGCCATACTACGAGGAAGCTAAT

GGAGGCGGTGGAACTGACCTGGGCAAGTTTGTACTTGCCAAAAAGGAGGAAAATGCTAAA

GCTTGA

>BN25-2

ATGACCAAGCTAGTTCTGTACACGCTACACCTAAGCCCACCATGCCGGGCCGTCGAACTG

ACAGCCAAAGCGTTGGGATTGGAACTGGAACAGAAGAATATTAAACTTCTGGCGGGTGAT

CATTTGACGCCGGAGTTCATGAAGTTAAACCCCCAACATACGATCCCGGTGCTGGATGAT

GATGGTACGATCATTACCGAGAGTCATGCGATCATGATCTATCTGGTGACGAAGTATGGC

AAAGATGACACCCTGTACCCAAAAGATCCAGTCCAGCAGGCTCGCGTAAATGCTGCCCTA

CACTTTGAATCTGGTGTACTGTTTGCACGAATGCGTTTCATTTTTGAGCGTATTTTTTTC

TACGGAAAATCGGACATTCCCGAAGATCGAGTCGAGTATGTGCAGAAATCGTACCGCTTG

CTGGAGGACACCCTAAAGGATGACTTTGTTGCTGGGTCGAAAATGACAATTGCCGACTTT

AGCTGCATTTCTACCATCTCTAGCATTATGGGCGTTGTTCCGCTGGAGCAATCGGAGCAT

CCACGTATCTATGAGTGGATCGATCGTTTGAAGCAGTTGCCATACTACGAGGAAGCTAAT

GGAGGCGGTGGAACTGACCTGGGCAAGTTTGTACTTGCCAAAAAGGAGGAAAATGCTAAA

GCTTGA

>BN26-1

ATGACCAAGCTAGTTCTGTACACGCTACACCTAAGCCCACCATGCCGGGCCGTCGAACTG

ACAGCCAAAGCGTTGGGATTGGAACTGGAACAGAAGAATATTAACCTTCTGGCGGGTGAT

CATTTGACGCCGGAGTTCATGAAGTTAAACCCCCAACATACGATCCCGGTGCTGGATGAT

GATGGTACGATCATTACCGAGAGTCATGCGATCATGATCTATCTGGTGACGAAGTATGGC

AAAGATGACACCCTGTACCCAAAAGATCCAGTCCAGCAGGCTCGCGTAAATGCTGCCCTA

CACTTTGAATCTGGTGTACTGTTTGCACGAATGCGTTTCATTTTTGAGCGTATTTTTTTC

TACGGAAAATCGGACATTCCCGAAGATCGAGTCGAGTATGTGCAGAAATCGTACCGCTTG

CTGGAGGACACCCTAAAGGATGACTTTGTTGCTGGGTCGAAAATGACAATTGCCGACTTT

AGCTGCATTTCTACCATCTCTAGCATTATGGGCGTTGTTCCGCTGGAGCAATCGGAGCAT

CCACGTATCTATGAGTGGATCGATCGTTTGAAGCAGTTGCCATACTACGAGGAAGCTAAT

GGAGGCGGTGGAACTGACCTGGGCAAGTTTGTACTTGCCAAAAAGGAGGAAAATGCTAAA

GCTTGA

>BN26-2

ATGACCAAGCTAGTTCTGTACACGCTACACCTAAGCCCACCATGCCGGGCCGTCGAACTG

ACAGCCAAAGCGTTGGGATTGGAACTGGAACAGAAGAATATTAACCTTCTGGCGGGTGAT

CATTTGACGCCGGAGTTCATGAAGTTAAACCCCCAACATACGATCCCGGTGCTGGATGAT

GATGGTACGATCATTACCGAGAGTCATGCGATCATGATCTATCTGGTGACGAAGTATGGC

AAAGATGACACCCTGTACCCAAAAGATCCAGTCCAGCAGGCTCGCGTAAATGCTGCCCTA

CACTTTGAATCTGGTGTACTGTTTGCACGAATGCGTTTCATTTTTGAGCGTATTTTTTTC

TACGGAAAATCGGACATTCCCGAAGATCGAGTCGAGTATGTGCAGAAATCGTACCGCTTG

CTGGAGGACACCCTAAAGGATGACTTTGTTGCTGGGTCGAAAATGACAATTGCCGACTTT

AGCTGCATTTCTACCATCTCTAGCATTATGGGCGTTGTTCCGCTGGAGCAATCGGAGCAT

CCACGTATCTATGAGTGGATCGATCGTTTGAAGCAGTTGCCATACTACGAGGAAGCTAAT

GGAGGCGGTGGAACTGACCTGGGCAAGTTTGTACTTGCCAAAAAGGAGGAAAATGCTAAA

GCTTGA

>CAM9-1

ATGACCAAGCTAGTTCTGTACACGCTACACCTAAGCCCACCATGCCGGGCCGTCGAACTG

ACAGCCAAAGCGTTGGGATTGGAACTGGAACAGAAGAATATTAACCTTCTGGCTGGTGAT

CATTTGACGCCGGAGTTCATGAAGTTAAACCCCCAACATACGATCCCGGTGCTGGATGAT

GATGGTACGATCATTACCGAGAGTCATGCGATCATGATCTATCTGGTGACGAAGTATGGC

AAAGATGACACCCTGTACCCAAAAGATCCAGTCCAGCAGGCTCGCGTAAATGCTGCCCTA

CACTTTGAATCTGGTGTACTGTTTGCACGAATGCGTTTCATTTTTGAGCGTATTCTTTTC

TACGGAAAATCGGACATTCCCGAAGATCGAGTCGAGTATGTGCAGAAATCGTACCGCTTG

CTGGAGGACACCCTAAAGGATGACTTTGTTGCTGGGTCGAAAATGACAATTGCCGACTTT

AGCTGCATTTCTACCATCTCTAGCATTATGGGCGTTGTTCCGCTGGAGCAATCGGAGCAT

CCACGTATCTATGAGTGGATCGATCGTTTGAAGCAGTTGCCATACTACGAGGAAGCTAAT

GGAGGCGGTGGAACTGACCTGGGCAAGTTTGTACTTGCCAAAAAGGAGGAAAATGCTAAA

GCTTGA

>CAM9-2

ATGACCAAGCTAGTTCTGTACACGCTACACCTAAGCCCACCATGCCGGGCCGTCGAACTG

ACAGCCAAAGCGTTGGGATTGGAACTGGAACAGAAGAATATTAACCTTCTGGCTGGTGAT

CATTTGACGCCGGAGTTCATGAAGTTAAACCCCCAACATACGATCCCGGTGCTGGATGAT

GATGGTACGATCATTACCGAGAGTCATGCGATCATGATCTATCTGGTGACGAAGTATGGC

AAAGATGACACCCTGTACCCAAAAGATCCAGTCCAGCAGGCTCGCGTAAATGCTGCCCTA

CACTTTGAATCTGGTGTACTGTTTGCACGAATGCGTTTCATTTTTGAGCGTATTCTTTTC

TACGGAAAATCGGACATTCCCGAAGATCGAGTCGAGTATGTGCAGAAATCGTACCGCTTG

CTGGAGGACACCCTAAAGGATGACTTTGTTGCTGGGTCGAAAATGACAATTGCCGACTTT

AGCTGCATTTCTACCATCTCTAGCATTATGGGCGTTGTTCCGCTGGAGCAATCGGAGCAT

CCACGTATCTATGAGTGGATCGATCGTTTGAAGCAGTTGCCATACTACGAGGAAGCTAAT

GGAGGCGGTGGAACTGACCTGGGCAAGTTTGTACTTGCCAAAAAGGAGGAAAATGCTAAA

GCTTGA

>CAM10-1

ATGACCAAGCTAGTTCTGTACACGCTACACCTAAGCCCACCATGCCGGGCCGTCGAACTG

ACAGCCAAAGCGTTGGGATTGGAACTGGAACAGAAGAATATTAACCTTCTGGCTGGTGAT

CATTTGACGCCGGAGTTCATGAAGTTAAACCCCCAACATACGATCCCGGTGCTGGATGAT

GATGGTACGATCATTACCGAGAGTCATGCGATCATGATCTATCTGGTGACGAAGTATGGC

AAAGATGACACCCTGTACCCAAAAGATCCAGTCCAGCAGGCTCGCGTAAATGCTGCCCTA

CACTTTGAATCTGGTGTACTGTTTGCACGAATGCGTTTCATTTTTGAGCGTATTCTTTTC

TACGGAAAATCGGACATTCCCGAAGATCGAGTCGAGTATGTGCAGAAATCGTACCGCTTG

CTGGAGGACACCCTAAAGGATGACTTTGTTGCTGGGTCGAAAATGACAATTGCCGACTTT

AGCTGCATTTCTACCATCTCTAGCATTATGGGCGTTGTTCCGCTGGAGCAATCGGAGCAT

CCACGTATCTATGAGTGGATCGATCGTTTGAAGCAGTTGCCATACTACGAGGAAGCTAAT

GGAGGCGGTGGAACTGACCTGGGCAAGTTTGTACTTGCCAAAAAGGAGGAAAATGCTAAA

GCTTGA

>CAM10-2

ATGACCAAGCTAGTTCTGTACACGCTACACCTAAGCCCACCATGCCGGGCCGTCGAACTG

ACAGCCAAAGCGTTGGGATTGGAACTGGAACAGAAGAATATTAACCTTCTGGCGGGTGAT

CATTTGACGCCGGAGTTCATGAAGTTAAACCCCCAACATACGATCCCGGTGCTGGATGAT

GATGGTACGATCATTACCGAGAGTCATGCGATCATGATCTATCTGGTGACGAAGTATGGC

AAAGATGACACCCTGTACCCAAAAGATCCAGTCCAGCAGGCTCGCGTAAATGCTGCCCTA

CACTTTGAATCTGGTGTACTGTTTGCACGAATGCGTTTCATTTTTGAGCGTATTTTTTTC

TACGGAAAATCGGACATTCCCGAAGATCGAGTCGAGTATGTGCAGAAATCGTACCGCTTG

CTGGAGGACACCCTAAAGGATGACTTTGTTGCTGGGTCGAAAATGACAATTGCCGACTTT

AGCTGCATTTCTACCATCTCTAGCATTATGGGCGTTGTTCCGCTGGAGCAATCGGAGCAT

CCACGTATCTATGAGTGGATCGATCGTTTGAAGCAGTTGCCATACTACGAGGAAGCTAAT

GGAGGCGGTGGAACTGACCTGGGCAAGTTTGTACTTGCCAAAAAGGAGGAAAATGCTAAA

GCTTGA

>CAM11-1

ATGACCAAGCTAGTTCTGTACACGCTACACCTAAGCCCACCATGCCGGGCCGTCGAACTG

ACAGCCAAAGCGTTGGGATTGGAACTGGAACAGAAGAATATTAACCTTCTGGCTGGTGAT

CATTTGACGCCGGAGTTCATGAAGTTAAACCCCCAACATACGATCCCGGTGCTGGATGAT

GATGGTACGATCATTACCGAGAGTCATGCGATCATGATCTATCTGGTGACGAAGTATGGC

AAAGATGACACCCTGTACCCAAAAGATCCAGTCCAGCAGGCTCGCGTAAATGCTGCCCTA

CACTTTGAATCTGGTGTACTGTTTGCACGAATGCGTTTCATTTTTGAGCGTATTCTTTTC

TACGGAAAATCGGACATTCCCGAAGATCGAGTCGAGTATGTGCAGAAATCGTACCGCTTG

CTGGAGGACACCCTAAAGGATGACTTTGTTGCTGGGTCGAAAATGACAATTGCCGACTTT

AGTTGCATTTCTACCATCTCTAGCATTATGGGCGTTGTTCCGCTGGAGCAATCGGAGCAT

CCACGTATCTATGAGTGGATCGATCGTTTGAAGCAGTTGCCATACTACGAGGAAGCTAAT

GGAGGCGGTGGAACTGACCTGGGCAAGTTTGTACTTGCCAAAAAGGAGGAAAATGCTAAA

GCTTGA

>CAM11-2

ATGACCAAGCTAGTTCTGTACACGCTACACCTAAGCCCACCATGCCGGGCCGTCGAACTG

ACAGCCAAAGCGTTGGGATTGGAACTGGAACAGAAGAATATTAACCTTCTGGCGGGTGAT

CATTTGACGCCGGAGTTCATGAAGTTAAACCCCCAACATACGATCCCGGTGCTGGATGAT

GATGGTACGATCATTACCGAGAGTCATGCGATCATGATCTATCTGGTGACGAAGTATGGC

AAAGATGACACCCTGTACCCAAAAGATCCAGTCCAGCAGGCTCGCGTAAATGCTGCCCTA

CACTTTGAATCTGGTGTACTGTTTGCACGAATGCGTTTCATTTTTGAGCGTATTTTTTTC

TACGGAAAATCGGACATTCCCGAAGATCGAGTCGAGTATGTGCAGAAATCGTACCGCTTG

CTGGAGGACACCCTAAAGGATGACTTTGTTGCTGGGTCGAAAATGACAATTGCCGACTTT

AGCTGCATTTCTACCATCTCTAGCATTATGGGCGTTGTTCCGCTGGAGCAATCGGAGCAT

CCACGTATCTATGAGTGGATCGATCGTTTGAAGCAGTTGCCATACTACGAGGAAGCTAAT

GGAGGCGGTGGAACTGACCTGGGCAAGTTTGTACTTGCCAAAAAGGAGGAAAATGCTAAA

GCTTGA

>CAM14-1

ATGACCAAGCTAGTTCTGTACACGCTACACCTAAGCCCACCATGCCGGGCCGTCGAACTG

ACAGCCAAAGCGTTGGGATTGGAACTGGAACAGAAGAATATTAACCTTCTGGCTGGTGAT

CATTTGACGCCGGAGTTCATGAAGTTAAACCCCCAACATACGATCCCGGTGCTGGATGAT

GATGGTACGATCATTACCGAGAGTCATGCGATCATGATCTATCTGGTGACGAAGTATGGC

AAAGATGACACCCTGTACCCAAAAGATCCAGTCCAGCAGGCTCGCGTAAATGCTGCCCTA

CACTTTGAATCTGGTGTACTGTTTGCACGAATGCGTTTCATTTTTGAGCGTATTCTTTTC

TACGGAAAATCGGACATTCCCGAAGATCGAGTCGAGTATGTGCAGAAATCGTACCGCTTG

CTGGAGGACACCCTAAAGGATGACTTTGTTGCTGGGTCGAAAATGACAATTGCCGACTTT

AGCTGCATTTCTACCATCTCTAGCATTATGGGCGTTGTTCCGCTGGAGCAATCGGAGCAT

CCACGTATCTATGAGTGGATCGATCGTTTGAAGCAGTTGCCATACTACGAGGAAGCTAAT

GGAGGCGGTGGAACTGACCTGGGCAAGTTTGTACTTGCCAAAAAGGAGGAAAATGCTAAA

GCTTGA

>CAM14-2

ATGACCAAGCTAGTTCTGTACACGCTACACCTAAGCCCACCATGCCGGGCCGTCGAACTG

ACAGCCAAAGCGTTGGGATTGGAACTGGAACAGAAGAATATTAACCTTCTGGCGGGTGAT

CATTTGACGCCGGAGTTCATGAAGTTAAACCCCCAACATACGATCCCGGTGCTGGATGAT

GATGGTACGATCATTACCGAGAGTCATGCGATCATGATCTATCTGGTGACGAAGTATGGC

AAAGATGACACCCTGTACCCAAAAGATCCAGTCCAGCAGGCTCGCGTAAATGCTGCCCTA

CACTTTGAATCTGGTGTACTGTTTGCACGAATGCGTTTCATTTTTGAGCGTATTCTTTTC

TACGGAAAATCGGACATTCCCGAAGATCGAGTCGAGTATGTGCAGAAATCGTACCGCTTG

CTGGAGGACACCCTAACGGATGACTTTGTTGCTGGGTCGAAAATGACAATTGCCGACTTT

AGCTGCATTTCTACCATCTCTAGCATTATGGGCGTTGTTCCGCTGGAGCAATCGGAGCAT

CCACGTATCTATGAGTGGATCGATCGTTTGAAGCAGTTGCCATACTACGAGGAAGCTAAT

GGAGGCGGTGGAACTGACCTGGGCAAGTTTGTACTTGCCAAAAAGGAGGAAAATGCTAAA

GCTTGA

>CAM37-1

ATGACCAAGCTAGTTCTGTACACGCTACACCTAAGCCCACCATGCCGGGCCGTCGAACTG

ACAGCCAAAGCGTTGGGATTGGAACTGGAACAGAAGAATATTAACCTTCTGGCGGGTGAT

CATTTGACGCCGGAGTTCATGAAGTTAAACCCCCAACATACGATCCCGGTGCTGGATGAT

GATGGTACGATCATTACCGAGAGTCATGCGATCATGATCTATCTGGTGACGAAGTATGGC

AAAGATGACACCCTGTACCCAAAAGATCCAGTCCAGCAGGCTCGCGTAAATGCTGCCCTA

CACTTTGAATCTGGTGTACTGTTTGCACGAATGCGTTTCATTTTTGAGCGTATTTTTTTC

TACGGAAAATCGGACATTCCCGAAGATCGAGTCGAGTATGTGCAGAAATCGTACCGCTTG

CTGGAGGACACCCTAAAGGATGACTTTGTTGCTGGGTCGAAAATGACAATTGCCGACTTT

AGCTGCATTTCTACCATCTCTAGCATTATGGGCGTTGTTCCGCTGGAGCAATCGGAGCAT

CCACGTATCTATGAGTGGATCGATCGTTTGAAGCAGTTGCCATACTACGAGGAAGCTAAT

GGAGGCGGTGGAACTGACCTGGGCAAGTTTGTACTTGCCAAAAAGGAGGAAAATGCTAAA

GCTTGA

>CAM37-2

ATGACCAAGCTAGTTCTGTACACGCTACACCTAAGCCCACCATGCCGGGCCGTCGAACTG

ACAGCCAAAGCGTTGGGATTGGAACTGGAACAGAAGAATATTAACCTTCTGGCGGGTGAT

CATTTGACGCCGGAGTTCATGAAGTTAAACCCCCAACATACGATCCCGGTGCTGGATGAT

GATGGTACGATCATTACCGAGAGTCATGCGATCATGATCTATCTGGTGACGAAGTATGGC

AAAGATGACACCCTGTACCCAAAAGATCCAGTCCAGCAGGCTCGCGTAAATGCTGCCCTA

CACTTTGAATCTGGTGTACTGTTTGCACGAATGCGTTTCATTTTTGAGCGTATTTTTTTC

TACGGAAAATCGGACATTCCCGAAGATCGAGTCGAGTATGTGCAGAAATCGTACCGCTTG

CTGGAGGACACCCTAAAGGATGACTTTGTTGCTGGGTCGAAAATGACAATTGCCGACTTT

AGCTGCATTTCTACCATCTCTAGCATTATGGGCGTTGTTCCGCTGGAGCAATCGGAGCAT

CCACGTATCTATGAGTGGATCGATCGTTTGAAGCAGTTGCCATACTACGAGGAAGCTAAT

GGAGGCGGTGGAACTGACCTGGGCAAGTTTGTACTTGCCAAAAAGGAGGAAAATGCTAAA

GCTTGA

>GH10-1

ATGACCAAGCTAGTTCTGTACACGCTACACCTAAGCCCACCATGCCGGGCCGTCGAACTG

ACAGCCAAAGCGTTGGGATTGGAACTGGAACAGAAGAATATTAACCTTCTGGCTGGTGAT

CATTTGACGCCGGAGTTCATGAAGTTAAACCCCCAACATACGATCCCGGTGCTGGATGAT

GATGGTACGATCATTACCGAGAGTCATGCGATCATGATCTATCTGGTGACGAAGTATGGC

AAAGATGACACCCTGTACCCAAAAGATCCAGTCCAGCAGGCTCGCGTAAATGCTGCCCTA

CACTTTGAATCTGGTGTACTGTTTGCACGAATGCGTTTCATTTTTGAGCGTATTTTTTTC

TACGGAAAATCGGACATTCCCGAAGATCGAGTCGAGTATGTGCAGAAATCGTACCGCTTG

CTGGAGGACACCCTAAAGGATGACTTTGTTGCTGGGTCGAAAATGACAATTGCCGACTTT

AGCTGCATTTCTACCATCTCTAGCATTATGGGCGTTGTTCCGCTGGAGCAATCGGAGCAT

CCACGTATCTATGAGTGGATCGATCGTTTGAAGCAGTTGCCATACTACGAGGAAGCTAAT

GGAGGCGGTGGAACTGACCTGGGCAAGTTTGTACTTGCCAAAAAGGAGGAAAATGCTAAA

GCTTGA

>GH10-2

ATGACCAAGCTAGTTCTGTACACGCTACACCTAAGCCCACCATGCCGGGCCGTCGAACTG

ACAGCCAAAGCGTTGGGATTGGAACTGGAACAGAAGAATATTAACCTTCTGGCGGGTGAT

CATTTGACGCCGGAGTTCATGAAGTTAAACCCCCAACATACGATCCCGGTGCTGGATGAT

GATGGTACGATCATTACCGAGAGTCATGCGATCATGATCTATCTGGTGACGAAGTATGGC

AAAGATGACACCCTGTACCCAAAAGATCCAGTCCAGCAGGCTCGCGTAAATGCTGCCCTA

CACTTTGAATCTGGTGTACTGTTTGCACGAATGCGTTTCATTTTTGAGCGTATTTTTTTC

TACGGAAAATCGGACATTCCCGAAGATCGAGTCGAGTATGTGCAGAAATCGTACCGCTTG

CTGGAGGACACCCTAAAGGATGACTTTGTTGCTGGGTCGAAAATGACAATTGCCGACTTT

AGCTGCATTTCTACCATCTCTAGCATTATGGGCGTTGTTCCGCTGGAGCAATCGGAGCAT

CCACGTATCTATGAGTGGATCGATCGTTTGAAGCAGTTGCCATACTACGAGGAAGCTAAT

GGAGGCGGTGGAACTGACCTGGGCAAGTTTGTACTTGCCAAAAAGGAGGAAAATGCTAAA

GCTTGA

>GH12-1

ATGACCAAGCTAGTTCTGTACACGCTACACCTAAGCCCACCATGCCGGGCCGTCGAACTG

ACAGCCAAAGCGTTGGGATTGGAACTGGAACAGAAGAATATTAACCTTCTGGCGGGTGAT

CATTTGACGCCGGAGTTCATGAAGTTAAACCCCCAACATACGATCCCGGTGCTGGATGAT

GATGGTACGATCATTACCGAGAGTCATGCGATCATGATCTATCTGGTGACGAAGTATGGC

AAAGATGACACCCTGTACCCAAAAGATCCAGTCCAGCAGGCTCGCGTAAATGCTGCCCTA

CACTTTGAATCTGGTGTACTGTTTGCACGAATGCGTTTCATTTTTGAGCGTATTTTTTTC

TACGGAAAATCGGACATTCCCGAAGATCGAGTCGAGTATGTGCAGAAATCGTACCGCTTG

CTGGAGGACACCCTAAAGGATGACTTTGTTGCTGGGTCGAAAATGACAATTGCCGACTTT

AGCTGCATTTCTACCATCTCTAGCATTATGGGCGTTGTTCCGCTGGAGCAATCGGAGCAT

CCACGTATCTATGAGTGGATCGATCGTTTGAAGCAGTTGCCATACTACGAGGAAGCTAAT

GGAGGCGGTGGAACTGACCTGGGCAAGTTTGTACTTGCCAAAAAGGAGGAAAATGCTAAA

GCTTGA

>GH12-2

ATGACCAAGCTAGTTCTGTACACGCTACACCTAAGCCCACCATGCCGGGCCGTCGAACTG

ACAGCCAAAGCGTTGGGATTGGAACTGGAACAGAAGAATATTAACCTTCTGGCGGGTGAT

CATTTGACGCCGGAGTTCATGAAGTTAAACCCCCAACATACGATCCCGGTGCTGGATGAT

GATGGTACGATCATTACCGAGAGTCATGCGATCATGATCTATCTGGTGACGAAGTATGGC

AAAGATGACACCCTGTACCCAAAAGATCCAGTCCAGCAGGCTCGCGTAAATGCTGCCCTA

CACTTTGAATCTGGTGTACTGTTTGCACGAATGCGTTTCATTTTTGAGCGTATTTTTTTC

TACGGAAAATCGGACATTCCCGAAGATCGAGTCGAGTATGTGCAGAAATCGTACCGCTTG

CTGGAGGACACCCTAAAGGATGACTTTGTTGCTGGGTCGAAAATGACAATTGCCGACTTT

AGCTGCATTTCTACCATCTCTAGCATTATGGGCGTTGTTCCGCTGGAGCAATCGGAGCAT

CCACGTATCTATGAGTGGATCGATCGTTTGAAGCAGTTGCCATACTACGAGGAAGCTAAT

GGAGGCGGTGGAACTGACCTGGGCAAGTTTGTACTTGCCAAAAAGGAGGAAAATGCTAAA

GCTTGA

>GH21-1

ATGACCAAGCTAGTTCTGTACACGCTACACCTAAGCCCACCATGCCGGGCCGTCGAACTG

ACAGCCAAAGCGTTGGGATTGGAACTGGAACAGAAGAATATTAACCTTCTGGCTGGTGAT

CATTTGACGCCGGAGTTCATGAAGTTAAACCCCCAACATACGATCCCGGTGCTGGATGAT

GATGGTACGATCATTACCGAGAGTCATGCGATCATGATCTATCTGGTGACGAAGTATGGC

AAAGATGACACCCTGTACCCAAAAGATCCAGTCCAGCAGGCTCGCGTAAATGCTGCCCTA

CACTTTGAATCTGGTGTACTGTTTGCACGAATGCGTTTCATTTTTGAGCGTATTCTTTTC

TACGGAAAATCGGACATTCCCGAAGATCGAGTCGAGTATGTGCAGAAATCGTACCGCTTG

CTGGAGGACACCCTAAAGGATGACTTTGTTGCTGGGTCGAAAATGACAATTGCCGACTTT

AGCTGCATTTCTACCATCTCTAGCATTATGGGCGTTGTTCCGCTGGAGCAATCGGAGCAT

CCACGTATCTATGAGTGGATCGATCGTTTGAAGCAGTTGCCATACTACGAGGAAGCTAAT

GGAGGCGGTGGAACTGACCTGGGCAAGTTTGTACTTGCCAAAAAGGAGGAAAATGCTAAA

GCTTGA

>GH21-2

ATGACCAAGCTAGTTCTGTACACGCTACACCTAAGCCCACCATGCCGGGCCGTCGAACTG

ACAGCCAAAGCGTTGGGATTGGAACTGGAACAGAAGAATATTAACCTTCTGGCTGGTGAT

CATTTGACGCCGGAGTTCATGAAGTTAAACCCCCAACATACGATCCCGGTGCTGGATGAT

GATGGTACGATCATTACCGAGAGTCATGCGATCATGATCTATCTGGTGACGAAGTATGGC

AAAGATGACACCCTGTACCCAAAAGATCCAGTCCAGCAGGCTCGCGTAAATGCTGCCCTA

CACTTTGAATCTGGTGTACTGTTTGCACGAATGCGTTTCATTTTTGAGCGTATTCTTTTC

TACGGAAAATCGGACATTCCCGAAGATCGAGTCGAGTATGTGCAGAAATCGTACCGCTTG

CTGGAGGACACCCTAAAGGATGACTTTGTTGCTGGGTCGAAAATGACAATTGCCGACTTT

AGCTGCATTTCTACCATCTCTAGCATTATGGGCGTTGTTCCGCTGGAGCAATCGGAGCAT

CCACGTATCTATGAGTGGATCGATCGTTTGAAGCAGTTGCCATACTACGAGGAAGCTAAT

GGAGGCGGTGGAACTGACCTGGGCAAGTTTGTACTTGCCAAAAAGGAGGAAAATGCTAAA

GCTTGA

>GH24-1

ATGACCAAGCTAGTTCTGTACACGCTACACCTAAGCCCACCATGCCGGGCCGTCGAACTG

ACAGCCAAAGCGTTGGGATTGGAACTGGAACAGAAGAATATTAACCTTCTGGCTGGTGAT

CATTTGACGCCGGAGTTCATGAAGTTAAACCCCCAACACACGATCCCGGTGCTGGATGAT

GATGGTACGATCATTACCGAGAGTCATGCGATCATGATCTATCTGGTGACGAAGTATGGC

AAAGATGACACCCTGTACCCAAAAGATCCAGTCCAGCAGGCTCGCGTAAATGCTGCCCTA

CACTTTGAATCTGGTGTACTGTTTGCACGAATGCGTTTCATTTTTGAGCGTATTCTTTTC

TACGGAAAATCGGACATTCCCGAAGATCGAGTCGAGTATGTGCAGAAATCGTACCGCTTG

CTGGAGGACACCCTAAAGGATGACTTTGTTGCTGGGTCGAAAATGACAATTGCCGACTTT

AGCTGCATTTCTACCATCTCTAGCATTATGGGCGTTGTTCCGCTGGAGCAATCGGAGCAT

CCACGTATCTATGAGTGGATCGATCGTTTGAAGCAGTTGCCATACTACGAGGAAGCTAAT

GGAGGCGGTGGAACTGACCTGGGCAAGTTTGTACTTGCCAAAAAGGAGGAAAATGCTAAA

GCTTGA

>GH24-2

ATGACCAAGCTAGTTCTGTACACGCTACACCTAAGCCCACCATGCCGGGCCGTCGAACTG

ACAGCCAAAGCGTTGGGATTGGAACTGGAACAGAAGAATATTAACCTTCTGGCTGGTGAT

CATTTGACGCCGGAGTTCATGAAGTTAAACCCCCAACACACGATCCCGGTGCTGGATGAT

GATGGTACGATCATTACCGAGAGTCATGCGATCATGATCTATCTGGTGACGAAGTATGGC

AAAGATGACACCCTGTACCCAAAAGATCCAGTCCAGCAGGCTCGCGTAAATGCTGCCCTA

CACTTTGAATCTGGTGTACTGTTTGCACGAATGCGTTTCATTTTTGAGCGTATTCTTTTC

TACGGAAAATCGGACATTCCCGAAGATCGAGTCGAGTATGTGCAGAAATCGTACCGCTTG

CTGGAGGACACCCTAAAGGATGACTTTGTTGCTGGGTCGAAAATGACAATTGCCGACTTT

AGCTGCATTTCTACCATCTCTAGCATTATGGGCGTTGTTCCGCTGGAGCAATCGGAGCAT

CCACGTATCTATGAGTGGATCGATCGTTTGAAGCAGCTGCCATACTACGAGGAAGCTAAT

GGAGGCGGTGGAACTGACCTGGGCAAGTTTGTACTTGCCAAAAAGGAGGAAAATGCTAAA

GCTTGA

>GH25-1

ATGACCAAGCTAGTTCTGTACACGCTACACCTAAGCCCACCATGCCGGGCCGTCGAACTG

ACAGCCAAAGCGTTGGGATTGGAACTGGAACAGAAGAATATTAACCTTCTGGCGGGTGAT

CATTTGACGCCGGAGTTCATGAAGTTAAACCCCCAACATACGATCCCGGTGCTGGATGAT

GATGGTACGATCATTACCGAGAGTCATGCGATCATGATCTATCTGGTGACGAAGTATGGC

AAAGATGACACCCTGTACCCAAAAGATCCAGTCCAGCAGGCTCGCGTAAATGCTGCCCTA

CACTTTGAATCTGGTGTACTGTTTGCACGAATGCGTTTCATTTTTGAGCGTATTTTTTTC

TACGGAAAATCGGACATTCCCGAAGATCGAGTCGAGTATGTGCAGAAATCGTACCGCTTG

CTGGAGGACACCCTAAAGGATGACTTTGTTGCTGGGTCGAAAATGACAATTGCCGACTTT

AGCTGCATTTCTACCATCTCTAGCATTATGGGCGTTGTTCCGCTGGAGCAATCGGAGCAT

CCACGTATCTATGAGTGGATCGATCGTTTGAAGCAGTTGCCATACTACGAGGAAGCTAAT

GGAGGCGGTGGAACTGACCTGGGCAAGTTTGTACTTGCCAAAAAGGAGGAAAATGCTAAA

GCTTGA

>GH25-2

ATGACCAAGCTAGTTCTGTACACGCTACACCTAAGCCCACCATGCCGGGCCGTCGAACTG

ACAGCCAAAGCGTTGGGATTGGAACTGGAACAGAAGAATATTAACCTTCTGGCGGGTGAT

CATTTGACGCCGGAGTTCATGAAGTTAAACCCCCAACATACGATCCCGGTGCTGGATGAT

GATGGTACGATCATTACCGAGAGTCATGCGATCATGATCTATCTGGTGACGAAGTATGGC

AAAGATGACACCCTGTACCCAAAAGATCCAGTCCAGCAGGCTCGCGTAAATGCTGCCCTA

CACTTTGAATCTGGTGTACTGTTTGCACGAATGCGTTTCATTTTTGAGCGTATTTTTTTC

TACGGAAAATCGGACATTCCCGAAGATCGAGTCGAGTATGTGCAGAAATCGTACCGCTTG

CTGGAGGACACCCTAAAGGATGACTTTGTTGCTGGGTCGAAAATGACAATTGCCGACTTT

AGCTGCATTTCTACCATCTCTAGCATTATGGGCGTTGTTCCGCTGGAGCAATCGGAGCAT

CCACGTATCTATGAGTGGATCGATCGTTTGAAGCAGTTGCCATACTACGAGGAAGCTAAT

GGAGGCGGTGGAACTGACCTGGGCAAGTTTGTACTTGCCAAAAAGGAGGAAAATGCTAAA

GCTTGA

>Refseq-1

ATGACCAAGCTAGTTCTGTACACGCTACACCTAAGCCCACCATGCCGGGCCGTCGAACTG

ACAGCCAAAGCGTTGGGATTGGAACTGGAACAGAAGAATATTAACCTTCTGGCTGGTGAT

CATTTGACGCCGGAGTTCATGAAGTTAAACCCCCAACATACGATCCCGGTGCTGGATGAT

GATGGTACGATCATTACCGAGAGTCATGCGATCATGATCTATCTGGTGACGAAGTATGGC

AAAGATGACACCCTGTACCCAAAAGATCCAGTCCAGCAGGCTCGCGTAAATGCTGCCCTA

CACTTTGAATCTGGTGTACTGTTTGCACGAATGCGTTTCATTTTTGAGCGTATTCTTTTC

TACGGAAAATCGGACATTCCCGAAGATCGAGTCGAGTATGTGCAGAAATCGTACCGCTTG

CTGGAGGACACCCTAAAGGATGACTTTGTTGCTGGGTCGAAAATGACAATTGCCGACTTT

AGCTGCATTTCTACCATCTCTAGCATTATGGGCGTTGTTCCGCTGGAGCAATCGGAGCAT

CCACGTATCTATGAGTGGATCGATCGTTTGAAGCAGTTGCCATACTACGAGGAAGCTAAT

GGAGGCGGTGGAACTGACCTGGGCAAGTTTGTACTTGCCAAAAAGGAGGAAAATGCTAAA

GCTTGA

>Refseq-2

ATGACCAAGCTAGTTCTGTACACGCTACACCTAAGCCCACCATGCCGGGCCGTCGAACTG

ACAGCCAAAGCGTTGGGATTGGAACTGGAACAGAAGAATATTAACCTTCTGGCTGGTGAT

CATTTGACGCCGGAGTTCATGAAGTTAAACCCCCAACATACGATCCCGGTGCTGGATGAT

GATGGTACGATCATTACCGAGAGTCATGCGATCATGATCTATCTGGTGACGAAGTATGGC

AAAGATGACACCCTGTACCCAAAAGATCCAGTCCAGCAGGCTCGCGTAAATGCTGCCCTA

CACTTTGAATCTGGTGTACTGTTTGCACGAATGCGTTTCATTTTTGAGCGTATTCTTTTC

TACGGAAAATCGGACATTCCCGAAGATCGAGTCGAGTATGTGCAGAAATCGTACCGCTTG

CTGGAGGACACCCTAAAGGATGACTTTGTTGCTGGGTCGAAAATGACAATTGCCGACTTT

AGCTGCATTTCTACCATCTCTAGCATTATGGGCGTTGTTCCGCTGGAGCAATCGGAGCAT

CCACGTATCTATGAGTGGATCGATCGTTTGAAGCAGTTGCCATACTACGAGGAAGCTAAT

GGAGGCGGTGGAACTGACCTGGGCAAGTTTGTACTTGCCAAAAAGGAGGAAAATGCTAAA

GCTTGA

>1K1_RR-1

ATGACCAAGCTAGTTCTGTACACGCTACACCTAAGCCCACCATGCCGGGCCGTCGAACTG

ACAGCCAAAGCGTTGGGATTGGAACTGGAGCAGAAGACCATTAATCTTCTGGCCGGTGAT

CATTTGACGCCGGAGTTCATGAAGCTAAACCCACAGCATACGATCCCAGTGCTGGATGAT

GATGGTACGATCATCACGGAGAGTCATGCGATCATGATCTATCTGGTAACGAAGTATGCC

AAGGATGACACCCTGTACCCAAAAGATCCAGTTCAGCAGGCACGCGTAAATGCTGCCCTA

CACTTTGAGTCGGGTGTGCTGTTTGCACGAATGCGTTTCATTTTTGAGCGTATCCTTTTC

TACGGCAAATCGGATATTCCCGAAGATCGAGTCGAGTACGTGCAGAAATCGTACCGCTTG

CTGGAGGACACACTGGTGGACGATTTTGTTGCTGGAGCGGAGATGACAATTGCCGACTTT

AGCTGCATTTCGACAATCTCCAGCATTATGGGCGTTGTGCCGCTGGACCAATCGGAATAT

CCACAGATTTATGGGTGGATCACTCGTTTGAAGCAGCTGCCATACTACGAGGAAGCCAAT

GGTGGCGGTGGCACCGACCTGGGCAAGTTTGTACTTGCCAAAAAGGAGGAAAATGCTAAA

GCTTGA

>1K1_RR-2

ATGACCAAGCTAGTTCTGTACACGCTACACCTAAGCCCACCATGCCGGGCCGTCGAACTG

ACAGCCAAAGCGTTGGGATTGGAACTAGAGCAGAAGACCATTAATCTTCTGGCCGGTGAT

CATTTGACGCCGGAGTTTATGAAGCTAAACCCACAGCATACGATCCCAGTGCTGGATGAT

GATGGTACGATCATCACGGAGAGTCATGCGATCATGATCTATCTGGTAACGAAGTATGCC

AAGGATGACACCCTGTACCCAAAAGATCCAGTTCAGCAGGCACGCGTGAATGCTGCCCTA

CACTTTGAGTCGGGTGTGCTGTTTGCACGAATGCGTTTCATTTTTGAGCGTATCCTTTTC

TACGGCAAATCGGATATTCCCGAAGATCGAGTCGAGTACGTGCAGAAATCGTACCGCTTG

CTGGAGGACACACTGGTGGACGATTTTGTTGCTGGAGCGGAGATGACAATTGCTGACTTT

AGCTGCATTTCGACAATCTCCAGCATTATGGGCGTTGTGCCGCTGGACCAATCGGAATAT

CCACAGATTTATGGGTGGATCACTCGTTTGAAGCAGCTGCCATACTACGAGGAAGCCAAT

GGTGGCGGTGGCACCGACCTGGGCAAGTTTGTACTTGCCAAAAAGGAGGAAAATGCTAAA

GCTTGA

>2K2_RR-1

ATGACCAAGCTAGTTCTGTACACGCTACACCTAAGCCCACCATGCCGGGCCGTCGAACTG

ACAGCCAAAGCGTTGGGATTGGAACTGGAGCAGAAGACCATTAATCTTCTGGCCGGTGAT

CATTTGACACCGGAGTTTATGAAGCTAAACCCACAGCATACGATCCCAGTGCTGGATGAT

GATGGTACGATCATCACGGAGAGTCATGCGATCATGATCTATCTGGTAACGAAGTATGCC

AAGGATGACACCCTGTACCCAAAAGATCCAGTTCAGCAGGCACGCGTAAATGCTGCCCTA

CACTTTGAGTCGGGTGTGCTGTTTGCACGAATGCGTTTCATTTTTGAGCGTATCCTTTTC

TACGGCAAATCGGATATTCCCGAAGATCGAGTCGAGTACGTGCAGAAATCGTACCGCTTG

CTGGAGGACACACTGGTGAACGATTTTGTTGCTGGAGCGAAGATGACAATTGCTGACTTT

AGCTGCATTTCGACAATCTCCAGCATTATGGGCGTTGTGCCGCTGGACCAATCGGAATAT

CCACAGATTTATGGGTGGATCAATCGTTTGAAGCAGCTGCCATACTACGAGGAAGCCAAT

GGTGGCGGTGGCACCGACCTGGGCAAGTTTGTACTTGCCAAAAAGGAGGAAAATGCTAAA

GCTTGA

>2K2_RR-2

ATGACCAAGCTAGTTCTGTACACGCTACACCTAAGCCCACCATGCCGGGCCGTCGAACTG

ACAGCCAAAGCGTTGGGATTGGAACTGGAGCAGAAGACCATTAATCTTCTGGCCGGTGAT

CATTTGACACCGGAGTTTATGAAGCTAAACCCACAGCATACGATCCCAGTGCTGGATGAT

GATGGTACAATCATCACGGAAAGTCATGCAATCATGATCTATCTGGTAACGAAGTATGCC

AAGGATGACACCCTGTACCCAAAAGATCCAGTTCAGCAGGCACGCGTAAATGCTGCCCTA

CACTTTGAGTCGGGTGTGCTGTTTGCACGAATGCGTTTCATTTTTGAGCGTATCCTTTTC

TACGGCAAATCGGATATTCCCGAAGATCGAGTCGAGTACGTGCAGAAATCGTACCGCTTG

CTGGAGGACACACTGGTGAACGATTTTGTTGCTGGAGCGAAGATGACAATTGCTGACTTT

AGCTGCATTTCGACAATCTCCAGCATTATGGGCGTTGTGCCGCTGGACCAATCGGAATAT

CCACAGATTTATGGATGGATCAATCGTTTGAAGCAGCTGCCATACTACAAGGAAGCCAAT

GGTGGCGGTGGCACCGACCTGGGCAAGTTTGTACTTGCCAAAAAGGAGGAAAATGCTAAA

GCTTGA

>5K14_RR-1

ATGACCAAGCTAGTTCTGTACACGCTACACCTAAGCCCACCATGCCGGGCCGTCGAACTG

ACAGCCAAAGCGTTGGGATTGGAACTGGAGCAGAAGACCATTAATCTTCTGGCCGGTGAT

CATTTGACGCCGGAGTTCATGAAGCTAAACCCACAGCATACGATCCCAGTGCTGGATGAT

GATGGTACGATCATCACGGAGAGTCATGCGATCATGATCTATCTGGTAACGAAGTATGCC

AAGGATGACACCCTGTACCCAAAAGATCCAGTTCAGCAGGCACGCGTAAATGCTGCCCTA

CACTTTGAGTCGGGTGTGCTGTTTGCACGAATGCGTTTCATTTTTGAGCGTATCCTTTTC

TACGGCAAATCGGATATTCCCGAAGATCGAGTCGAGTACGTGCAGAAATCGTACCGCTTG

CTGGAGGACACACTGGTGGACGATTTTGTTGCTGGAGCGAAGATGACAATTGCCGACTTT

AGCTGCATTTCGACAATCTCCAGCATTATGGGCGTTGTGCCGCTGGACCAATCGGAATAT

CCACAGATTTATGGGTGGATCAATCGTTTGAAGCAGCTGCCATACTACGAGGAAGCCAAT

GGTGGCGGTGGCACCGACCTGGGCAAGTTTGTACTTGCCAAAAAGGAGGAAAATGCTAAA

GCTTGA

>5K14_RR-2

ATGACCAAGCTAGTTCTGTACACGCTACACCTAAGCCCACCATGCCGGGCCGTCGAACTG

ACAGCCAAAGCGTTGGGATTGGAACTGGAGCAGAAGACCATTAATCTTCTGGCCGGTGAT

CATTTGACGCCGGAGTTCATGAAGCTAAACCCACAGCATACGATCCCAGTGCTGGATGAT

GATGGTACGATCATCACGGAGAGTCATGCGATCATGATCTATCTGGTAACGAAGTATGCC

AAGGATGACACCCTGTACCCAAAAGATCCAGTTCAGCAGGCACGCGTAAATGCTGCCCTA

CACTTTGAGTCGGGTGTGCTGTTTGCACGAATGCGTTTCATTTTTGAGCGTATCCTTTTC

TACGGCAAATCGGATATTCCCGAAGATCGAGTCGAGTACGTGCAGAAATCGTACCGCTTG

CTGGAGGACACACTGGTGAACGATTTTGTTGCTGGAGCGAAGATGACAATTGCTGACTTT

AGCTGCATTTCGACAATCTCCAGCATTATGGGCGTTGTGCCGCTGGACCAATCGGAATAT

CCACAGATTTATGGGTGGATCAATCGTTTGAAGCAGCTGCCATACTACGAGGAAGCCAAT

GGTGGCGGTGGCACCGACCTGGGCAAGTTTGTACTTGCCAAAAAGGAGGAAAATGCTAAA

GCTTGA

>6K41_RR-1

ATGACCAAGCTAGTTCTGTACACGCTACACCTAAGCCCACCATGCCGGGCCGTCGAACTG

ACAGCCAAAGCGTTGGGATTGGAACTGGAACAGAAGAATATTAACCTTCTGGCTGGTGAT

CATTTGACGCCGGAGTTCATGAAGTTAAACCCCCAACATACGATCCCGGTGCTGGATGAT

GATGGTACGATCATTACCGAGAGTCATGCGATCATGATCTATCTGGTGACGAAGTATGGC

AAAGATGACACCCTGTACCCAAAAGATCCAGTCCAGCAGGCTCGCGTAAATGCTGCCCTA

CACTTTGAATCTGGTGTACTGTTTGCACGAATGCGTTTCATTTTTGAGCGTATTCTTTTC

TACGGAAAATCGGACATTCCCGAAGATCGAGTCGAGTATGTGCAGAAATCGTACCGCTTG

CTGGAGGACACCCTAAAGGATGACTTTGTTGCTGGGTCGAAAATGACAATTGCCGACTTT

AGCTGCATTTCTACCATCTCTAGCATTATGGGCGTTGTTCCGCTGGAGCAATCGGAGCAT

CCACGTATCTATGAGTGGATCGATCGTTTGAAGCAGTTGCCATACTACGAGGAAGCTAAT

GGAGGCGGTGGAACTGACCTGGGCAAGTTTGTACTTGCCAAAAAGGAGGAAAATGCTAAA

GCTTGA

>6K41_RR-2

ATGACCAAGCTAGTTCTGTACACGCTACACCTAAGCCCACCATGCCGGGCCGTCGAACTG

ACAGCCAAAGCGTTGGGATTGGAACTGGAACAGAAGAATATTAACCTTCTGGCTGGTGAT

CATTTGACGCCGGAGTTCATGAAGTTAAACCCCCAACATACAATCCCGGTGCTGGATGAT

GATGGTACGATCATTACCGAGAGTCATGCGATCATGATCTATCTGGTGACGAAGTATGGC

AAAGATGACACCCTGTACCCAAAAGATCCAGTCCAGCAGGCTCGCGTAAATGCTGCCCTA

CACTTTGAATCTGGTGTACTGTTTGCACGAATGCGTTTCATTTTTGAGCGTATTCTTTTC

TACGGAAAATCGGACATTCCCGAAGATCGAGTCGAGTATGTGCAGAAATCGTACCGCTTG

CTGGAGGACACCCTAAAGGATGACTTTGTTGCTGGGTCGAAAATGACAATTGCCGACTTT

AGCTGCATTTCTACCATCTCTAGCATTATGGGCGTTGTTCCGCTGGAGCAATCGGAGCAT

CCACGTATCTATGAGTGGATCGATCGTTTGAAGCAGTTGCCATACTACGAGGAAGCTAAT

GGAGGCGGTGGAACTGACCTGGGCAAGTTTGTACTTGCCAAAAAGGAGGAAAATGCTAAA

GCTTGA

>7K106_RR-1

ATGACCAAGCTAGTTCTGTACACGCTACACCTAAGCCCACCATGCCGGGCCGTCGAACTG

ACAGCCAAAGCGTTGGGATTGGAACTGGAGCAGAAGACCATTAATCTTCTGGCCGGTGAT

CATTTGACGCCGGAGTTCATGAAGCTAAACCCACAGCATACGATCCCAGTGCTGGATGAT

GATGGTACGATCATCACGGAGAGTCATGCGATCATGATCTATCTGGTAACGAAGTATGCC

AAGGATGACACCCTGTACCCAAAAGATCCAGTTCAGCAGGCACGCGTAAATGCTGCCCTA

CACTTTGAGTCGGGTGTGCTGTTTGCACGAATGCGTTTCATTTTTGAGCGTATCCTTTTC

TACGGCAAATCGGATATTCCCGAAGATCGAGTCGAGTACGTGCAGAAATCGTACCGCTTG

CTGGAGGACACACTGGTGGACGATTTTGTTGCTGGAGCGAAGATGACAATTGCTGACTTT

AGCTGCATTTCGACAATCTCCAGCATTATGGGCGTTGTGCCGCTGGACCAATCGGAATAT

CCACAGATTTATGGGTGGATCAATCGTTTGAAGCAGCTGCCATACTACGAGGAAGCCAAT

GGTGGCGGTGGCACCGACCTGGGCAAGTTTGTACTTGCCAAAAAGGAGGAAAATGCTAAA

GCTTGA

>7K106_RR-2

ATGACCAAGCTAGTTCTGTACACGCTACACCTAAGCCCACCATGCCGGGCCGTCGAACTG

ACAGCCAAAGCGTTGGGATTGGAACTGGAGCAGAAGACCATTAATCTTCTGGCCGGTGAT

CATTTGACGCCGGAGTTCATGAAGCTAAACCCACAGCATACGATCCCAGTGCTGGATGAT

GATGGTACGATCATCACGGAGAGTCATGCGATTATGATCTATCTGGTAACGAAGTATGCC

AAGGATGACACCCTGTACCCAAAAGATCCAGTTCAGCAGGCACGCGTAAATGCTGCCCTA

CACTTTGAGTCGGGTGTGCTGTTTGCACGAATGCGTTTCATTTTTGAGCGTATCCTTTTC

TACGGCAAATCGGATATTCCCGAAGATCGAGTCGAGTACGTGCAGAAATCGTACCGCTTG

CTGGAGGACACACTGGTGAACGATTTTGTTGCTGGAGCGAAGATGACAATTGCTGACTTT

AGCTGCATTTCGACAATCTCCAGCATTATGGGCGTTGTGCCGCTGGACCAATCGGAATAT

CCACAGATTTATGGGTGGATCAATCGTTTGAAGCAGCTGCCATACTACGAGGAAGCCAAT

GGTGGCGGTGGCACCGACCTGGGCAAGTTTGTACTTGCCAAAAAGGAGGAAAATGCTAAA

GCTTGA

>8K18_RR-1

ATGACCAAGCTAGTTCTGTACACGCTACACCTAAGCCCACCATGCCGGGCCGTCGAACTG

ACAGCCAAAGCGTTGGGATTGGAACTGGAGCAGAAGACCATTAATCTTCTGGCCGGTGAT

CATTTGACGCCGGAGTTCATGAAGCTAAACCCACAGCATACGATCCCAGTGCTGGATGAT

GATGGTACGATCATCACGGAGAGTCATGCGATCATGATCTATCTGGTAACGAAGTATGCC

AAGGATGACACCCTGTACCCAAAAGATCCAGTTCAGCAGGCACGCGTAAATGCTGCCCTA

CACTTTGAGTCGGGTGTGCTGTTTGCACGAATGCGTTTCATTTTTGAGCGTATCCTTTTC

TACGGCAAATCGGATATTCCCGAAGATCGAGTCGAGTACGTGCAGAAATCGTACCGCTTG

CTGGAGGACACACTGGTGAACGATTTTGTTGCTGGAGCGAAGATGACAATTGCTGACTTT

AGCTGCATTTCGACAATCTCCAGCATTATGGGCGTTGTGCCGCTGGACCAATCGGAATAT

CCACAGATTTATGGGTGGATCAATCGTTTGAAGCAGCTGCCATACTACGAGGAAGCCAAT

GGTGGCGGTGGCACCGACCTGGGCAAGTTTGTACTTGCCAAAAAGGAGGAAAATGCTAAA

GCTTGA

>8K18_RR-2

ATGACCAAGCTAGTTCTGTACACGCTACACCTAAGCCCACCATGCCGGGCCGTCGAACTG

ACAGCCAAAGCGTTGGGATTGGAACTGGAGCAGAAGACCATTAATCTTCTGGCCGGTGAT

CATTTGACGCCGGAGTTCATGAAGCTAAACCCACAGCATACGATCCCAGTGCTGGATGAT

GATGGTACGATCATCACGGAGAGTCATGCGATCATGATCTATCTGGTAACGAAGTATGCC

AAGGATGACACCCTGTACCCAAAAGATCCAGTTCAGCAGGCACGCGTAAATGCTGCCCTA

CACTTTGAGTCGGGTGTGCTGTTTGCACGAATGCGTTTCATTTTTGAGCGTATCCTTTTC

TACGGCAAATCGGATATTCCCGAAGATCGAGTCGAGTACGTGCAGAAATCGTACCGCTTG

CTGGAGGACACACTGGTGAACGATTTTGTTGCTGGAGCGAAGATGACAATTGCTGACTTT

AGCTGCATTTCGACAATCTCCAGCATTATGGGCGTTGTGCCGCTGGACCAATCGGAATAT

CCACAGATTTATGGGTGGATCAATCGTTTGAAGCAGCTGCCATACTACGAGGAAGCCAAT

GGTGGCGGTGGCACCGACCTGGGCAAGTTTGTACTTGCCAAAAAGGAGGAAAATGCTAAA

GCTTGA

>10K57_SS-1

ATGACCAAGCTAGTTCTGTACACGCTACACCTAAGCCCACCATGCCGGGCCGTCGAACTG

ACAGCCAAAGCGTTGGGATTGGAACTGGAACAGAAGAATATTAACCTTCTGGCTGGTGAT

CATTTGACGCCGGAGTTCATGAAGTTAAACCCCCAACATACGATCCCGGTGCTGGATGAT

GATGGTACGATCATTACCGAGAGTCATGCGATCATGATCTATCTGGTGACGAAGTATGGC

AAAGATGACACCCTGTACCCAAAAGATCCAGTCCAGCAGGCTCGCGTAAATGCTGCCCTA

CACTTTGAATCTGGTGTACTGTTTGCACGAATGCGTTTCATTTTTGAGCGTATTCTTTTC

TACGGAAAATCGGACATTCCCGAAGATCGAGTCGAGTATGTGCAGAAATCGTACCGCTTG

CTGGAGGACACCCTAAAGGATGACTTTGTTGCTGGGTCGAAAATGACAATTGCCGACTTT

AGCTGCATTTCTACCATCTCTAGCATTATGGGCGTTGTTCCGCTGGAGCAATCGGAGCAT

CCACGTATCTATGAGTGGATCGATCGTTTGAAGCAGTTGCCATACTACGAGGAAGCTAAT

GGAGGCGGTGGAACTGACCTGGGCAAGTTTGTACTTGCCAAAAAGGAGGAAAATGCTAAA

GCTTGA

>10K57_SS-2

ATGACCAAGCTAGTTCTGTACACGCTACACCTAAGCCCACCATGCCGGGCCGTCGAACTG

ACAGCCAAAGCGTTGGGATTGGAACTGGAACAGAAGAATATTAACCTTCTGGCTGGTGAT

CATTTGACGCCGGAGTTCATGAAGTTAAACCCCCAACATACAATCCCGGTGCTGGATGAT

GATGGTACGATCATTACCGAGAGTCATGCGATCATGATCTATCTGGTGACGAAGTATGGC

AAAGATGACACCCTGTACCCAAAAGATCCAGTCCAGCAGGCTCGCGTAAATGCTGCCCTA

CACTTTGAATCTGGTGTACTGTTTGCACGAATGCGTTTCATTTTTGAGCGTATTCTTTTC

TACGGAAAATCGGACATTCCCGAAGATCGAGTCGAGTATGTGCAGAAATCGTACCGCTTG

CTGGAGGACACCCTAAAGGATGACTTTGTTGCTGGGTCGAAAATGACAATTGCCGACTTT

AGCTGCATTTCTACCATCTCTAGCATTATGGGCGTTGTTCCGCTGGAGCAATCGGAGCAT

CCACGTATCTATGAGTGGATCGATCGTTTGAAGCAGTTGCCATACTACGAGGAAGCTAAT

GGAGGCGGTGGAACTGACCTGGGCAAGTTTGTACTTGCCAAAAAGGAGGAAAATGCTAAA

GCTTGA

>11Ma3_RR-1

ATGACCAAGCTAGTTCTGTACACGCTACACCTAAGCCCACCATGCCGGGCCGTCGAACTG

ACAGCCAAAGCGTTGGGATTGGAACTGGAGCAGAAGACCATTAATCTTCTGGCCGGTGAT

CATTTGACGCCGGAGTTCATGAAGCTAAACCCACAGCATACGATCCCAGTGCTGGATGAT

GATGGTACGATCATCACGGAGAGTCATGCGATCATGATCTATCTGGTAACGAAGTATGCC

AAGGATGACACCCTGTACCCAAAAGATCCAGTTCAGCAGGCACGCGTAAATGCTGCCCTA

CACTTTGAGTCGGGTGTGCTGTTTGCACGAATGCGTTTCATTTTTGAGCGTATCCTTTTC

TACGGCAAATCGGATATTCCCGAAGATCGAGTCGAGTACGTGCAGAAATCGTACCGCTTG

CTGGAGGACACACTGGTGGACGATTTTGTTGCTGGAGCGGAGATGACAATTGCCGACTTT

AGCTGCATTTCGACAATCTCCAGCATTATGGGCGTTGTGCCGCTGGACCAATCGGAATAT

CCACAGATTTATGGGTGGATCAATCGTTTGAAGCAACTGCCATACTACGAGGAAGCCAAT

GGTGGCGGTGGCACCGACCTGGGCAAGTTTGTACTTGCCAAAAAGGAGGAAAATGCTAAA

GCTTGA

>11Ma3_RR-2

ATGACCAAGCTAGTTCTGTACACGCTACACCTAAGCCCACCATGCCGGGCCGTCGAACTG

ACAGCCAAAGCGTTGGGATTGGAACTGGAGCAGAAGACCATTAATCTTCTGGCCGGTGAT

CATTTGACACCGGAGTTTATGAAGCTAAACCCACAGCATACGATCCCAGTGCTGGATGAT

GATGGTACGATCATCACGGAGAGTCATGCGATCATGATCTATCTGGTAACGAAGTATGCC

AAGGATGACACCCTGTACCCAAAAGATCCAGTTCAGCAGGCACGCGTAAATGCTGCCCTA

CACTTTGAGTCGGGTGTGCTGTTTGCACGAATGCGTTTCATTTTTGAGCGTATCCTTTTC

TACGGCAAATCGGATATTCCCGAAGATCGAGTCGAGTACGTGCAGAAATCGTACCGCTTG

CTGGAGGACACACTGGTGAACGATTTTGTTGCTGGAGCGAAGATGACAATTGCTGACTTT

AGCTGCATTTCGACAATCTCCAGCATTATGGGCGTTGTGCCGCTGGACCAATCGGAATAT

CCACAGATTTATGGGTGGATCAATCGTTTGAAGCAGCTGCCATACTACGAGGAAGCCAAT

GGTGGCGGTGGCACCGACCTGGGCAAGTTTGTACTTGCCAAAAAGGAGGAAAATGCTAAA

GCTTGA

>12Ma10_RR-1

ATGACCAAGCTAGTTCTGTACACGCTACACCTAAGCCCACCATGCCGGGCCGTCGAACTG

ACAGCCAAAGCGTTGGGATTGGAACTGGAGCAGAAGACCATTAATCTTCTGGCCGGTGAT

CATTTGACGCCGGAGTTCATGAAGCTAAACCCACAGCATACGATCCCAGTGCTGGATGAT

GATGGTACGATCATCACGGAGAGTCATGCGATTATGATCTATCTGGTAACGAAGTATGCC

AAGGATGACACCCTGTACCCAAAAGATCCAGTTCAGCAGGCACGCGTAAATGCTGCCCTA

CACTTTGAGTCGGGTGTGCTGTTTGCACGAATGCGTTTCATTTTTGAGCGTATCCTTTTC

TACGGCAAATCGGATATTCCCGAAGATCGAGTCGAGTACGTGCAGAAATCGTACCGCTTG

CTGGAGGACACACTGGTGAACGATTTTGTTGCTGGAGCGAAGATGACAATTGCTGACTTT

AGCTGCATTTCGACAATCTCCAGCATTATGGGCGTTGTGCCGCTGGACCAATCGGAATAT

CCACAGATTTATGGGTGGATCAATCGTTTGAAGCAGCTGCCATACTACGAGGAAGCCAAT

GGTGGCGGTGGCACCGACCTGGGCAAGTTTGTACTTGCCAAAAAGGAGGAAAATGCTAAA

GCTTGA

>12Ma10_RR-2

ATGACCAAGCTAGTTCTGTACACGCTACACCTAAGCCCACCATGCCGGGCCGTCGAACTG

ACAGCCAAAGCGTTGGGATTGGAACTGGAGCAGAAGACCATTAATCTTCTGGCCGGTGAT

CATTTGACACCGGAGTTTATGAAGCTAAACCCACAGCATACGATCCCAGTGCTGGATGAT

GATGGTACGATCATCACGGAGAGTCATGCGATCATGATCTATCTGGTAACGAAGTATGCC

AAGGATGACACCCTGTACCCAAAAGATCCAGTTCAGCAGGCACGCGTAAATGCTGCCCTA

CACTTTGAGTCGGGTGTGCTGTTTGCACGAATGCGTTTCATTTTTGAGCGTATCCTTTTC

TACGGCAAATCGGATATTCCCGAAGATCGAGTCGAGTACGTGCAGAAATCGTACCGCTTG

CTGGAGGACACACTGGTGAACGATTTTGTTGCTGGAGCGAAGATGACAATTGCTGACTTT

AGCTGCATTTCGACAATCTCCAGCATTATGGGCGTTGTGCCGCTGGACCAATCGGAATAT

CCACAGATTTATGGGTGGATCAATCGTTTGAAGCAGCTGCCATACTACGAGGAAGCCAAT

GGTGGCGGTGGCACCGACCTGGGCAAGTTTGTACTTGCCAAAAAGGAGGAAAATGCTAAA

GCTTGA

>14Ma20_RR-1

ATGACCAAGCTAGTTCTGTACACGCTACACCTAAGCCCACCATGCCGGGCCGTCGAACTG

ACAGCCAAAGCGTTGGGATTGGAACTGGAGCAGAAGACCATTAATCTTCTGGCCGGTGAT

CATTTGACGCCGGAGTTCATGAAGCTAAACCCACAGCATACGATCCCAGTGCTGGATGAT

GATGGTACGATCATCACGGAGAGTCATGCGATCATGATCTATCTGGTAACGAAGTATGCC

AAGGATGACACCCTGTACCCAAAAGATCCAGTTCAGCAGGCACGCGTAAATGCTGCCCTA

CACTTTGAGTCGGGTGTGCTGTTTGCACGAATGCGTTTCATTTTTGAGCGTATCCTTTTC

TACGGCAAATCGGATATTCCCGAAGATCGAGTCGAGTACGTGCAGAAATCGTACCGCTTG

CTGGAGGACACACTGGTGGACGATTTTGTTGCTGGAGCGAAGATGACAATTGCCGACTTT

AGCTGCATTTCGACAATCTCCAGCATTATGGGCGTTGTGCCGCTGGACCAATCGGAATAT

CCACAGATTTATGGGTGGATCAATCGTTTGAAGCAGCTGTCATACTACGAGGAAGCCAAT

GGTGGCGGTGGCACCGACCTGGGCAAGTTTGTACTTGCCAAAAAGGAGGAAAATGCTAAA

GCTTGA

>14Ma20_RR-2

ATGACCAAGCTAGTTCTGTACACGCTACACCTAAGCCCACCATGCCGGGCCGTCGAACTG

ACAGCCAAAGCGTTGGGATTGGAACTGGAGCAGAAGACCATTAATCTTCTGGCCGGTGAT

CATTTGACACCGGAGTTTATGAAGCTAAACCCACAGCATACGATCCCAGTGCTGGATGAT

GATGGTACGATCATCACGGAGAGTCATGCGATCATGATCTATCTGGTAACGAAGTATGCC

AAGGATGACACCCTGTACCCAAAAGATCCAGTTCAGCAGGCACGCGTAAATGCTGCCCTA

CACTTTGAGTCGGGTGTGCTGTTTGCACGAATGCGTTTCATTTTTGAGCGTATCCTTTTC

TACGGCAAATCGGATATTCCCGAAGATCGAGTCGAGTACGTGCAGAAATCGTACCGCTTG

CTGGAGGACACACTGGTGAACGATTTTGTTGCTGGAGCGAAGATGACAATTGCTGACTTT

AGCTGCATTTCGACAATCTCCAGCATTATGGGCGTTGTGCCGCTGGACCAATCGGAATAT

CCACAGATTTATGGGTGGATCAATCGTTTGAAGCAGCTGCCATACTACGAGGAAGCCAAT

GGTGGCGGTGGCACCGACCTGGGCAAGTTTGTACTTGCCAAAAAGGAGGAAAATGCTAAA

GCTTGA

>16Ma25_RR-1

ATGACCAAGCTAGTTCTGTACACGCTACACCTAAGCCCACCATGCCGGGCCGTCGAACTG

ACAGCCAAAGCGTTGGGATTGGAACTGGAGCAGAAGACCATTAATCTTCTGGCCGGTGAT

CATTTGACGCCGGAGTTCATGAAGCTAAACCCACAGCATACGATCCCAGTGCTGGATGAT

GATGGTACGATCATCACGGAGAGTCATGCGATCATGATCTATCTGGTAACGAAGTATGCC

AAGGATGACACCCTGTACCCAAAAGATCCAGTTCAGCAGGCACGCGTAAATGCTGCCCTA

CACTTTGAGTCGGGTGTGCTGTTTGCACGAATGCGTTTCATTTTTGAGCGTATCCTTTTC

TACGGCAAATCGGATATTCCCGAAGATCGAGTCGAGTACGTGCAGAAATCGTACCGCTTG

CTGGAGGACACACTGGTGGACGATTTTGTTGCTGGAGCGGAGATGACAATTGCCGACTTT

AGCTGCATTTCGACAATCTCCAGCATTATGGGCGTTGTGCCGCTGGACCAATCGGAATAT

CCACAGATTTATGGGTGGATCAATCGTTTGAAGCAGCTGCCATACTACGAGGAAGCCAAT

GGTGGCGGTGGCACCGACCTGGGCAAGTTTGTACTTGCCAAAAAGGAGGAAAATGCTAAA

GCTTGA

>16Ma25_RR-2

ATGACCAAGCTAGTTCTGTACACGCTACACCTAAGCCCACCATGCCGGGCCGTCGAACTG

ACAGCCAAAGCGTTGGGATTGGAACTGGAGCAGAAGACCATTAATCTTCTGGCCGGTGAT

CATTTGACGCCGGAGTTCATGAAGCTAAACCCACAGCATACGATCCCAGTGCTGGATGAT

GATGGTACGATCATCACGGAGAGTCATGCGATCATGATCTATCTGGTAACGAAGTATGCC

AAGGATGACACCCTGTACCCAAAAGATCCAGTTCAGCAGGCACGCGTAAATGCTGCCCTA

CACTTTGAGTCGGGTGTGCTGTTTGCACGAATGCGTTTCATTTTTGAGCGTATCCTTTTC

TACGGCAAATCGGATATTCCCGAAGATCGAGTCGAGTACGTGCAGAAATCGTACCGCTTG

CTGGAGGACACACTGGTGGACGATTTTGTTGCTGGAGCGGAGATGACAATTGCTGACTTT

AGCTGCATTTCGACAATCTCCAGCATTATGGGCGTTGTGCCGCTGGACCAATCGGAATAT

CCACAGATTTATGGGTGGATCAATCGTTTGAAGCAGCTGCCATACTACGAGGAAGCCAAT

GGTGGTGGTGGCACCGACCTGGGCAAGTTTGTACTTGCCAAAAAGGAGGAAAATGCTAAA

GCTTGA

>17Ma27_RR-1

ATGACCAAGCTAGTTCTGTACACGCTACACCTAAGCCCACCATGCCGGGCCGTCGAACTG

ACAGCCAAAGCGTTGGGATTGGAACTGGAGCAGAAGACCATTAATCTTCTGGCCGGTGAT

CATTTGACGCCGGAGTTCATGAAGCTAAACCCACAGCATACGATCCCAGTGCTGGATGAT

GATGGTACGATCATCACGGAGAGTCATGCGATCATGATCTATCTGGTAACGAAGTATGCC

AAGGATGACACCCTGTACCCAAAAGATCCAGTTCAGCAGGCACGCGTAAATGCTGCCCTA

CACTTTGAGTCGGGTGTGCTGTTTGCACGAATGCGTTTCATTTTTGAGCGTATCCTTTTC

TACGGCAAATCGGATATTCCCGAAGATCGAGTCGAGTACGTGCAGAAATCGTACCGCTTG

CTGGAGGACACACTGGTGGACGATTTTGTTGCTGGAGCGGAGATGACAATTGCCGACTTT

AGCTGCATTTCGACAATCTCCAGCATTATGGGCGTTGTGCCGCTGGACCAATCGGAATAT

CCACAGATTTATGGGTGGATCAATCGTTTGAAGCAGCTGTCATACTACGAGGAAGCCAAT

GGTGGCGGTGGCACCGACCTGGGCAAGTTTGTACTTGCCAAAAAGGAGGAAAATGCTAAA

GCTTGA

>17Ma27_RR-2

ATGACCAAGCTAGTTCTGTACACGCTACACCTAAGCCCACCATGCCGGGCCGTCGAACTG

ACAGCCAAAGCGTTGGGATTGGAACTGGAGCAGAAGACCATTAATCTTCTGGCCGGTGAT

CATTTGACGCCGGAGTTCATGAAGCTAAACCCACAGCATACGATCCCAGTGCTGGATGAT

GATGGTACGATCATCACGGAGAGTCATGCGATCATGATCTATCTGGTAACGAAGTATGCC

AAGGATGACACCCTGTACCCAAAAGATCCAGTTCAGCAGGCACGCGTAAATGCTGCCCTA

CACTTTGAGTCGGGTGTGCTGTTTGCACGAATGCGTTTCATTTTTGAGCGTATCCTTTTC

TACGGCAAATCGGATATTCCCGAAGATCGAGTCGAGTACGTGCAGAAATCGTACCGCTTG

CTGGAGGACACACTGGTGAACGATTTTGTTGCTGGAGCGAAGATGACAATTGCTGACTTT

AGCTGCATTTCGACAATCTCCAGCATTATGGGCGTTGTGCCGCTGGACCAATCGGAATAT

CCACAGATTTATGGGTGGATCAATCGTTTGAAGCAGCTGCCATACTACGAGGAAGCCAAT

GGTGGCGGTGGCACCGACCTGGGCAAGTTTGTACTTGCCAAAAAGGAGGAAAATGCTAAA

GCTTGA

>18Ma32_RR-1

ATGACCAAGCTAGTTCTGTACACGCTACACCTAAGCCCACCATGCCGGGCCGTCGAACTG

ACAGCCAAAGCGTTGGGATTGGAACTGGAGCAGAAGACCATTAATCTTCTGGCCGGTGAT

CATTTGACGCCGGAGTTCATGAAGCTAAACCCACAGCATACGATCCCAGTGCTGGATGAT

GATGGTACGATCATCACGGAGAGTCATGCGATCATGATCTATCTGGTAACGAAGTATGCC

AAGGATGACACCCTGTACCCAAAAGATCCAGTTCAGCAGGCACGCGTAAATGCTGCCCTA

CACTTTGAGTCGGGTGTGCTGTTTGCACGAATGCGTTTCATTTTTGAGCGTATCCTTTTC

TACGGCAAATCGGATATTCCCGAAGATCGAGTCGAGTACGTGCAGAAATCGTACCGCTTG

CTGGAGGACACACTGGTGAACGATTTTGTTGCTGGAGCGAAGATGACAATTGCTGACTTT

AGCTGCATTTCGACAATCTCCAGCATTATGGGCGTTGTGCCGCTGGACCAATCGGAATAT

CCACAGATTTATGGGTGGATCAATCGTTTGAAGCAGCTGCCATACTACGAGGAAGCCAAT

GGTGGCGGTGGCACCGACCTGGGCAAGTTTGTACTTGCCAAAAAGGAGGAAAATGCTAAA

GCTTGA

>18Ma32_RR-2

ATGACCAAGCTAGTTCTGTACACGCTACACCTAAGCCCACCATGCCGGGCCGTCGAACTG

ACAGCCAAAGCGTTGGGATTGGAACTGGAGCAGAAGACCATTAATCTTCTGGCCGGTGAT

CATTTGACGCCAGAGTTCATGAAGCTAAACCCCCAGCATACGATCCCAGTGCTGGATGAT

GATGGTACGATCATCACGGAGAGTCATGCGATCATGATCTATCTGGTAACGAAGTATGCC

AAGGATGACACCCTGTACCCAAAAGATCCAGTTCAGCAGGCACGCGTAAATGCTGCCCTA

CACTTTGAGTCGGGTGTGCTGTTTGCACGAATGCGTTTCATTTTTGAGCGTATCCTTTTC

TACGGCAAATCGGATATTCCCGAAGATCGAGTCGAGTACGTGCAGAAATCGTACCGCTTG

CTGGAGGACACACTGGTGGACGATTTTGTTGCTGGAGCGAAGATGACAATTGCTGACTTT

AGCTGCATTTCGACAATCTCCAGCATTATGGGCGTTGTGCCGCTGGACCAATCGGAATAT

CCACAGATTTATGGGTGGATCAATCGTTTGAAGCAGCTGCCATACTACGAGGAAGCCAAT

GGTGGTGGTGGCACCGACCTGGGCAAGTTTGTACTTGCCAAAAAGGAGGAAAATGCTAAA

GCTTGA

>19Ma49_RR-1

ATGACCAAGCTAGTTCTGTACACGCTACACCTAAGCCCACCATGCCGGGCCGTCGAACTG

ACAGCCAAAGCGTTGGGATTGGAACTGGAGCAGAAGACCATTAATCTTCTGGCCGGTGAT

CATTTGACGCCGGAGTTCATGAAGCTAAACCCCCAGCATACGATCCCAGTGCTGGATGAT

GATGGTACGATCATCACGGAGAGTCATGCGATCATGATCTATCTGGTAACGAAGTATGCC

AAGGATGACACCCTGTACCCAAAAGATCCAGTTCAGCAGGCACGCGTAAATGCTGCACTA

CACTTTGAGTCGGGTGTGCTGTTTGCACGAATGCGTTTCATTTTTGAGCGTATCCTTTTC

TACGGCAAATCGGATATTCCCGAAGATCGAATCGAGTACGTGCAGAAATCGTACCGCTTG

CTGGAGGACACACTGGTGAACGATTTTGTTGCTGGAGCGAAGATGACAATTGCTGACTTT

AGCTGCATTTCGACAATCTCCAGCATTATGGGCGTTGTGCCGCTGGACCAATCGGAATAT

CCACAGATTTATGGGTGGATCAATCGTTTGAAGCAGCTGCCATACTACGAGGAAGCCAAT

GGTGGTGGTGGCACCGACCTGGGCAAGTTTGTACTTGCCAAAAAGGAGGAAAATGCTAAA

GCTTGA

>19Ma49_RR-2

ATGACCAAGCTAGTTCTGTACACGCTACACCTAAGCCCACCATGCCGGGCCGTCGAACTG

ACAGCCAAAGCGTTGGGATTGGAACTGGAGCAGAAGACCATTAATCTTCTGGCCGGTGAT

CATTTGACGCCGGAGTTCATGAAGCTAAACCCACAGCATACGATCCCAGTGCTGGATGAT

GATGGTACGATCATCACGGAGAGTCATGCGATCATGATCTATCTGGTAACGAAGTATGCC

AAGGATGACACCCTGTACCCAAAAGATCCAGTTCAGCAGGCACGCGTAAATGCTGCCCTA

CACTTTGAGTCGGGTGTGCTGTTTGCACGAATGCGTTTCATTTTTGAGCGTATCCTTTTC

TACGGCAAATCGGATATTCCCGAAGATCGAGTCGAGTACGTGCAGAAATCGTACCGCTTG

CTGGAGGACACACTGGTGGACGATTTTGTTGCTGGAGCGAAGATGACAATTGCCGACTTT

AGCTGCATTTCGACAATCTCCAGCATTATGGGCGTTGTGCCGCTGGACCAATCGGAATAT

CCACAGATTTATGGGTGGATCAATCGTTTGAAGCAGCTGCCATACTACGAGGAAGCCAAT

GGTGGCGGTGGCACCGACCTGGGCAAGTTTGTACTTGCCAAAAAGGAGGAAAATGCTAAA

GCTTGA

>23KK14_RS-1

ATGACCAAGCTAGTTCTGTACACGCTACACCTAAGCCCACCATGCCGGGCCGTCGAACTG

ACAGCCAAAGCGTTGGGATTGGAACTGGAACAGAAGAATATTAACCTTCTGGCTGGTGAT

CATTTGACGCCGGAGTTCATGAAGTTAAACCCCCAACATACGATCCCGGTGCTGGATGAT

GATGGTACGATCATTACCGAGAGTCATGCGATCATGATCTATCTGGTGACGAAGTATGGC

AAAGATGACACCCTGTACCCAAAAGATCCAGTCCAGCAGGCTCGCGTAAATGCTGCCCTA

CACTTTGAATCTGGTGTACTGTTTGCACGAATGCGTTTCATTTTTGAGCGTATTCTTTTC

TACGGAAAATCGGACATTCCCGAAGATCGAGTCGAGTATGTGCAGAAATCGTACCGCTTG

CTGGAGGACACCCTAAAGGATGACTTTGTTGCTGGGTCGAAAATGACAATTGCCGACTTT

AGCTGCATTTCTACCATCTCTAGCATTATGGGCGTTGTTCCGCTGGAGCAATCGGAGCAT

CCACGTATCTATGAGTGGATCGATCGTTTGAAGCAGCTGCCATACTACGAGGAAGCTAAT

GGAGGCGGTGGAACTGACCTGGGCAAGTTTGTACTTGCCAAAAAGGAGGAAAATGCTAAA

GCTTGA

>23KK14_RS-2

ATGACCAAGCTAGTTCTGTACACGCTACACCTAAGCCCACCATGCCGGGCCGTCGAACTG

ACAGCCAAAGCGTTGGGATTGGAACTGGAACAGAAGAATATTAACCTTCTGGCTGGTGAT

CATTTGACGCCGGAGTTCATGAAGTTAAACCCCCAACATACGATCCCGGTGCTGGATGAT

GATGGTACGATCATTACCGAGAGTCATGCGATCATGATCTATCTGGTGACGAAGTATGGC

AAAGATGACACCCTGTACCCAAAAGATCCAGTCCAGCAGGCTCGCGTAAATGCTGCCCTA

CACTTTGAATCTGGTGTACTGTTTGCACGAATGCGTTTCATTTTTGAGCGTATTCTTTTC

TACGGAAAATCGGACATTCCCGAAGATCGAGTCGAGTATGTGCAGAAATCGTACCGCTTG

CTGGAGGACACCCTAACGGATGACTTTGTTGCTGGGTCGAAAATGACAATTGCCGACTTT

AGCTGCATTTCTACCATCTCTAGCATTATGGGCGTTGTTCCGCTGGAGCAATCGGAGCAT

CCACGTATCTATGAGTGGATCGATCGTTTGAAGCAGCTGCCATACTACGAGGAAGCTAAT

GGAGGCGGTGGAACTGACCTGGGCAAGTTTGTACTTGCCAAAAAGGAGGAAAATGCTAAA

GCTTGA

>27KK24_RS-1

ATGACCAAGCTAGTTCTGTACACGCTACACCTAAGCCCACCATGCCGGGCCGTCGAACTG

ACAGCCAAAGCGTTGGGATTGGAACTGGAACAGAAGAATATTAACCTTCTGGCTGGTGAT

CATTTGACGCCGGAGTTCATGAAGTTAAACCCCCAACATACGATCCCGGTGCTGGATGAT

GATGGTACGATCATTACCGAGAGTCATGCGATCATGATCTATCTGGTGACGAAGTATGGC

AAAGATGACACCCTGTACCCAAAAGATCCAGTCCAGCAGGCTCGCGTAAATGCTGCCCTA

CACTTTGAATCTGGTGTACTGTTTGCACGAATGCGTTTCATTTTTGAGCGTATTCTTTTC

TACGGAAAATCGGACATTCCCGAAGATCGAGTCGAGTATGTGCAGAAATCGTACCGCTTG

CTGGAGGACACCCTAACGGATGACTTTGTTGCTGGGTCGAAAATGACAATTGCCGACTTT

AGCTGCATTTCTACCATCTCTAGCATTATGGGCGTTGTTCCGCTGGAGCAATCGGAGCAT

CCACGTATCTATGAGTGGATCGATCGTTTGAAGCAGCTGCCATACTACGAGGAAGCTAAT

GGAGGCGGTGGAACTGACCTGGGCAAGTTTGTACTTGCCAAAAAGGAGGAAAATGCTAAA

GCTTGA

>27KK24_RS-2

ATGACCAAGCTAGTTCTGTACACGCTACACCTAAGCCCACCATGCCGGGCCGTCGAACTG

ACAGCCAAAGCGTTGGGATTGGAACTGGAACAGAAGAATATTAACCTTCTGGCTGGTGAT

CATTTGACGCCGGAGTTCATGAAGTTAAACCCCCAACATACGATCCCGGTGCTGGATGAT

GATGGTACGATCATTACCGAGAGTCATGCGATCATGATCTATCTGGTGACGAAGTATGGC

AAAGATGACACCCTGTACCCAAAAGATCCAGTCCAGCAGGCTCGCGTAAATGCTGCCCTA

CACTTTGAATCTGGTGTACTGTTTGCACGAATGCGTTTCATTTTTGAGCGTATTCTTTTC

TACGGAAAATCGGACATTCCCGAAGATCGAGTCGAGTATGTGCAGAAATCGTACCGCTTG

CTGGAGGACACCCTAACGGATGACTTTGTTGCTGGGTCGAAAATGACAATTGCCGACTTT

AGCTGCATTTCTACCATCTCTAGCATTATGGGCGTTGTTCCGCTGGAGCAATCGGAGCAT

CCACGTATCTATGAGTGGATCGATCGTTTGAAGCAGCTGCCATACTACGAGGAAGCTAAT

GGAGGCGGTGGAACTGACCTGGGCAAGTTTGTACTTGCCAAAAAGGAGGAAAATGCTAAA

GCTTGA

>28KK25_RS-1

ATGACCAAGCTAGTTCTGTACACGCTACACCTAAGCCCACCATGCCGGGCCGTCGAACTG

ACAGCCAAAGCGTTGGGATTGGAACTGGAACAGAAGAATATTAACCTTCTGGCTGGTGAT

CATTTGACGCCGGAGTTCATGAAGTTAAACCCCCAACATACGATCCCGGTGCTGGATGAT

GATGGTACGATCATTACCGAGAGTCATGCGATCATGATCTATCTGGTGACGAAGTATGGC

AAAGATGACACCCTGTACCCAAAAGATCCAGTCCAGCAGGCTCGCGTAAATGCTGCCCTA

CACTTTGAATCTGGTGTACTGTTTGCACGAATGCGTTTCATTTTTGAGCGTATTCTTTTC

TACGGAAAATCGGACATTCCCGAAGATCGAGTCGAGTATGTGCAGAAATCGTACCGCTTG

CTGGAGGACACCCTAAAGGATGACTTTGTTGCTGGGTCGAAAATGACAATTGCCGACTTT

AGCTGCATTTCTACCATCTCTAGCATTATGGGCGTTGTTCCGCTGGAGCAATCGGAGCAT

CCACGTATCTATGAGTGGATCGATCGTTTGAAGCAGTTGCCATACTACGAGGAAGCTAAT

GGAGGCGGTGGAACTGACCTGGGCAAGTTTGTACTTGCCAAAAAGGAGGAAAATGCTAAA

GCTTGA

>28KK25_RS-2

ATGACCAAGCTAGTTCTGTACACGCTACACCTAAGCCCACCATGCCGGGCCGTCGAACTG

ACAGCCAAAGCGTTGGGATTGGAACTGGAACAGAAGAATATTAACCTTCTGGCTGGTGAT

CATTTGACGCCGGAGTTCATGAAGTTAAACCCCCAACATACGATCCCGGTGCTGGATGAT

GATGGTACGATCATTACCGAGAGTCATGCGATCATGATCTATCTGGTGACGAAGTATGGC

AAAGATGACACCCTGTACCCAAAAGATCCAGTCCAGCAGGCTCGCGTAAATGCTGCCCTA

CACTTTGAATCTGGTGTACTGTTTGCACGGATGCGTTTCATTTTTGAGCGTATTTTTTTC

TACGGAAAATCGGACATTCCCGAAGATCGAGTCGAGTATGTGCAGAAATCGTACCGCTTG

CTGGAGGACACCCTAAAGGATGACTTTGTTGCTGGGTCGAAAATGACAATTGCCGACTTT

AGCTGCATTTCTACCATCTCTAGCATTATGGGCGTTGTTCCGCTGGAGCAATCGGAGCAT

CCACGTATCTATGAGTGGATCGATCGTTTGAAGCAGTTGCCATACTACGAGGAAGCTAAT

GGAGGCGGTGGAACTGACCTGGGCAAGTTTGTACTTGCCAAAAAGGAGGAAAATGCTAAA

GCTTGA

>29KK28_SS-1

ATGACCAAGCTAGTTCTGTACACGCTACACCTAAGCCCACCATGCCGGGCCGTCGAACTG

ACAGCCAAAGCGTTGGGATTGGAACTGGAACAGAAGAATATTAACCTTCTGGCTGGTGAG

CATTTGACGCCGGAGTTCATGAAGTTAAACCCCCAACATACGATCCCGGTGCTGGATGAT

GATGGTACGATCATTACCGAGAGTCATGCGATCATGATCTATCTGGTGACGAAGTATGGC

AAAGATGACACCCTGTACCCAAAAGATCCAGTCCAGCAGGCTCGCGTAAATGCTGCCCTA

CACTTTGAATCTGGTGTACTGTTTGCACGAATGCGTTTCATTTTTGAGCGTATTCTTTTC

TACGGAAAATCGGACATTCCCGAAGATCGAGTCGAGTATGTGCAGAAATCGTACCGCTTG

CTGGAGGACACCCTAAAGGATGACTTTGTTGCTGGGTCGAAAATGACAATTGCCGACTTT

AGCTGCATTTCTACCATCTCTAGCATTATGGGCGTTGTTCCGCTGGAGCAATCGGAGCAT

CCACGTATCTATGAGTGGATCGATCGTTTGAAGCAGTTGCCATACTACGAGGAAGCTAAT

GGAGGCGGTGGAACTGACCTGGGCAAGTTTGTACTTGCCAAAAAGGAGGAAAATGCTAAA

GCTTGA

>29KK28_SS-2

ATGACCAAGCTAGTTCTGTACACGCTACACCTAAGCCCACCATGCCGGGCCGTCGAACTG

ACAGCCAAAGCGTTGGGATTGGAACTGGAACAGAAGAATATTAACCTTCTGGCTGGTGAG

CATTTGACGCCGGAGTTCATGAAGTTAAACCCCCAACATACGATCCCGGTGCTGGATGAT

GATGGTACGATCATTACCGAGAGTCATGCGATCATGATCTATCTGGTGACGAAGTATGGC

AAAGATGACACCCTGTACCCAAAAGATCCAGTCCAGCAGGCTCGCGTAAATGCTGCCCTA

CACTTTGAATCTGGTGTACTGTTTGCACGAATGCGTTTCATTTTTGAGCGTATTCTTTTC

TACGGAAAATCGGACATTCCCGAAGATCGAGTCGAGTATGTGCAGAAATCGTACCGCTTG

CTGGAGGACACCCTAAAGGATGACTTTGTTGCTGGGTCGAAAATGACAATTGCCGACTTT

AGCTGCATTTCTACCATCTCTAGCATTATGGGCGTTGTTCCGCCGGAGCAATCGGAGCAT

CCACGTATCTATGAGTGGATCGATCGTTTGAAGCAGTTGCCATACTACGAGGAAGCTAAT

GGAGGCGGTGGAACTGACCTGGGCAAGTTTGTACTTGCCAAAAAGGAGGAAAATGCTAAA

GCTTGA

>30KK29_SS-1

ATGACCAAGCTAGTTCTGTACACGCTACACCTAAGCCCACCATGCCGGGCCGTCGAACTG

ACAGCCAAAGCGTTGGGATTGGAACTGGAACAGAAGAATATTAACCTTCTGGCTGGTGAG

CATTTGACGCCGGAGTTCATGAAGTTAAACCCCCAACATACGATCCCGGTGCTGGATGAT

GATGGTACGATCATTACCGAGAGTCATGCGATCATGATCTATCTGGTGACGAAGTATGGC

AAAGATGACACCCTGTACCCAAAAGATCCAGTCCAGCAGGCTCGCGTAAATGCTGCCCTA

CACTTTGAATCTGGTGTACTGTTTGCACGAATGCGTTTCATTTTTGAGCGTATTCTTTTC

TACGGAAAATCGGACATTCCCGAAGATCGAGTCGAGTATGTGCAGAAATCGTACCGCTTG

CTGGAGGACACCCTAAAGGATGACTTTGTTGCTGGGTCGAAAATGACAATTGCCGACTTT

AGCTGCATTTCTACCATCTCTAGCATTATGGGCGTTGTTCCGCCGGAGCAATCGGAGCAT

CCACGTATCTATGAGTGGATCGATCGTTTGAAGCAGTTGCCATACTACGAGGAAGCTAAT

GGAGGCGGTGGAACTGACCTGGGCAAGTTTGTACTTGCCAAAAAGGAGGAAAATGCTAAA

GCTTGA

>30KK29_SS-2

ATGACCAAGCTAGTTCTGTACACGCTACACCTAAGCCCACCATGCCGGGCCGTCGAACTG

ACAGCCAAAGCGTTGGGATTGGAACTGGAACAGAAGAATATTAACCTTCTGGCTGGTGAG

CATTTGACGCCGGAGTTCATGAAGTTAAACCCCCAACATACGATCCCGGTGCTGGATGAT

GATGGTACGATCATTACCGAGAGTCATGCGATCATGATCTATCTGGTGACGAAGTATGGC

AAAGATGACACCCTGTACCCAAAAGATCCAGTCCAGCAGGCTCGCGTAAATGCTGCCCTA

CACTTTGAATCTGGTGTACTGTTTGCACGAATGCGTTTCATTTTTGAGCGTATTCTTTTC

TACGGAAAATCGGACATTCCCGAAGATCGAGTCGAGTATGTGCAGAAATCGTACCGCTTG

CTGGAGGACACCCTAAAGGATGACTTTGTTGCTGGGTCGAAAATGACAATTGCCGACTTT

AGCTGCATTTCTACCATCTCTAGCATTATGGGCGTTGTTCCGCCGGAGCAATCGGAGCAT

CCACGTATCTATGAGTGGATCGATCGTTTGAAGCAGTTGCCATACTACGAGGAAGCTAAT

GGAGGCGGTGGAACTGACCTGGGCAAGTTTGTACTTGCCAAAAAGGAGGAAAATGCTAAA

GCTTGA
